# Phytochemical Characterization and Anti-Biofilm Activity of *Primula veris* L. Roots

**DOI:** 10.3390/molecules30081702

**Published:** 2025-04-10

**Authors:** Antoaneta Trendafilova, Desislava Raykova, Viktoria Ivanova, Miroslav Novakovic, Paraskev Nedialkov, Tsvetelina Paunova-Krasteva, Ralitsa Veleva, Tanya Topouzova-Hristova

**Affiliations:** 1 Institute of Organic Chemistry with Centre of Phytochemistry, Bulgarian Academy of Sciences, 1113 Sofia, Bulgaria; desislava.raykova@orgchm.bas.bg (D.R.); viktoria.genova@orgchm.bas.bg (V.I.); 2 Institute of Chemistry, Technology and Metallurgy, National Institute of the Republic of Serbia, University of Belgrade, 11000 Belgrade, Serbia; mironov@chem.bg.ac.rs; 3 Pharmacognosy Department, Faculty of Pharmacy, Medical University of Sofia, 1000 Sofia, Bulgaria; pnedialkov@pharmfac.mu-sofia.bg; 4 Stephan Angeloff Institute of Microbiology, Bulgarian Academy of Sciences, 1113 Sofia, Bulgaria; pauny@abv.bg; 5 Department of Cellular and Developmental Biology, Faculty of Biology, Sofia University St Kliment Ohridski, 1164 Sofia, Bulgaria; ralitsa_veleva@biofac.uni-sofia.bg (R.V.); topouzova@biofac.uni-sofia.bg (T.T.-H.)

**Keywords:** *Primula veris* L., phenolic compounds, primulasaponin I, new primulasaponins, NMR, antibiofilm activity, cytotoxicity

## Abstract

In this study, three new undescribed triterpene saponins named primulasaponin III–V (**19**–**21**) were isolated from the roots of *Primula veris* L. of Bulgarian origin together with the known primulasaponin I. Their structures were elucidated via 1D and 2D NMR spectroscopy and HR-ESI-MS. In addition, 17 known phenolic compounds (six flavones, three acetophenones, four bisbibenzyls, and four phenolic glycosides) were identified in the chloroform and methanol extracts. Among them, flavone, 2′-methoxyflavone, 3′-methoxyflavone, 3′-hydroxy-4′,5′-dimethoxyflavone, 2′,5′-dimethoxyflavone, 3′-methoxy-4′,5′-methylendioxyflavone, paeonol, 2-primeverosyl-5-methoxy-acetophenone, and paeonolide were detected for the first time in the roots of *P. veris.* The minimum inhibitory and minimum bactericidal concentrations of the chloroform and methanol extracts of *P. veris* roots and the saponin-enriched fraction were determined, with MIC values ranging between 0.5 and 1 mg/mL. Additionally, the tested samples were evaluated for their ability to inhibit biofilm formation in the presence of sub-MICs. All tested samples showed better biofilm inhibition of Gram-negative strains compared to Gram-positive strains. The strongest effect was observed for the chloroform extract against the biofilm formation of *Pseudomonas aeruginosa*, while the saponin-enriched fraction showed the highest percentage of biofilm inhibition of *Escherichia coli*, *Staphylococcus aureus*, and *Staphylococcus mutans*. At the same time, chloroform extract showed lower cytotoxicity against human keranocyte cell line HaCaT, as compared with methanol extract and the saponin-enriched fraction.

## 1. Introduction

Genus *Primula* is the largest one in the Primulaceae family and is distributed in the temperate and cold regions of the Northern Hemisphere [1]. *Primula veris* L. (syn. *P. officinalis* (L.) Hill), commonly known as cowslip, is an herbaceous perennial plant native to almost all of Europe, with its area of inhabitance extending from the northern parts of the Mediterranean to Scandinavia, as well as large parts of Asia [2,3]. In folk medicine, the leaf and root extracts (infusion, decoction, or tincture) of *P. veris* and other *Primula* species are used to treat various respiratory diseases, such as coughs, bronchitis and colds, skin problems, wound healing, ulcers; for toothaches; and against snake poison [1,4,5,6,7]. *Primulae* radix and *Primulae* flos are listed in the European pharmacopeia, and their extracts are incorporated in various expectorant and diuretic drugs [8]. Additionally, primrose is a valuable horticultural plant often used as an ornamental species [9].

*Primula* species are known to produce various polyphenolic compounds such as flavonoids; mono-, di-, and tri-glycosides; phenolic acids; phenolic glycosides (primulaverin and primeverin); and epicuticular flavonoids [1,5,10]. The latter are accumulated on the external surface of the plant and are mainly aglycones substituted with hydroxy, methoxy, methylenedioxy, and acetyl groups. These compounds exhibit anti-asthmatic, anti-inflammatory, antiviral, antioxidant, and antigenotoxic activities [11,12,13,14]. Epicuticular flavonoids are considered to be chemotaxonomic markers for the genus [15,16,17,18,19]. Aside from polyphenols, cowslip is also known for its species-specific saponins. So far, primula acids (primulasaponin) I and II, priverosaponin B, priverosaponin B-22-acetate, and primacrosaponin have been isolated from *P. veris* [9,10,20,21,22,23]. The secretolytic and expectorant activity of *P. veris* is attributed to the presence of saponins [24]. Other compounds found in the *Primula* genus are bisbibenzyls, which were initially considered as chemical markers of liverworts but were identified for the first time in higher plants, specifically in *P. veris* subsp. *macrocalyx* from Mt. Altay in Russia [25]. Recently, riccardin C and other bisbibenzyls were found in Russian, Armenian, and Iranian *P. veris* subsp. *macrocalyx* [26] and Serbian *P. veris* subsp. *columnae* [27]. Bisbibenzyl derivatives exhibited diverse biological activities such as antitumor and anti-leukemia [28] and antibacterial and antifungal activities [29].

The increasing prevalence of biofilm-associated infections, which often lead to chronic conditions, has drawn significant attention from the scientific community in the search for suitable inhibitors of bacterial biofilms. According to data from the U.S. National Institutes of Health, approximately 65% of all bacterial infections and 80% of chronic infections are associated with biofilm formation in patients [30]. The main reason for this is that antimicrobial agents can be up to 1000 times less effective against biofilms compared to free-living bacterial cells, making biofilm eradication particularly challenging [31,32]. Furthermore, biofilm tolerance is attributed to both the composition of the exopolysaccharide matrix and the phenotypic heterogeneity within the biofilm, which consists of metabolically active and inactive cells [33,34]. Despite the diverse bioactivities of *Primula* species and their metabolites, there is a lack of information regarding their antibiofilm potential. To the best of our knowledge, there is only one report on the antibiofilm activity of *P. vulgaris* root extract against *Candida albicans* [35]. Literature data on the antibacterial properties of *Primula* species are also scarce and focused mainly on extracts obtained from the aerial parts of *P. veris* [36,37,38], *P. officinalis* [39], *P. vulgaris* [35,40,41,42], and some non-European *Primula* species [43,44,45]. There are only two reports on the antibacterial activity of *P*. *vulgaris* and *P*. *vulgaris* subsp. *rubra* roots [35,42].

In Bulgarian flora, the genus *Primula* is represented by eight species, one of which is *Primula veris* L. [46,47]. A literature survey revealed only three reports on Bulgarian populations of *P. veris*: a study on the reproductive potential (embryological features and pollen and seed viability) and genetic diversity of *P. veris* [48], the establishment of ex situ collection of *P. veris* [49], and the presence of exudate flavonoids in the leaves of *P. veris* and their inhibitory activity on *L. perrene* seed germination [50].

The insufficient data for the chemical content of the Bulgarian *Primula* populations prompted us to conduct in-depth phytochemical investigations, starting with the roots of *Primula veris* L. The second goal of this work was to expand the knowledge of the biological activity of cowslip by studying its antibiofilm properties.

## 2. Results and Discussion

### 2.1. Phytochemical Characterization of Primula veris

**Figure 1:**
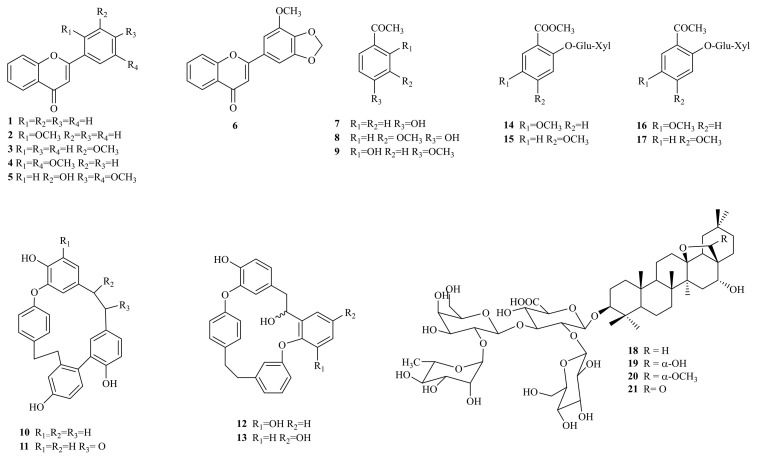
Structures of isolated compounds from *P. veris* roots.

The air-dried and ground roots of *P. veris* were successively extracted with chloroform and methanol to obtain the corresponding extracts. Further purification of the chloroform extract led to the isolation of 13 compounds belonging to three different classes, namely flavones (**1**–**6**), acetophenones (**7**–**9**), and bisbibenzyls (**10**–**13**) (Figure 1). A comparison of their ^1^H NMR data (Supplementary Part I) led to the identification of flavones (**1**) [51], 2′-methoxyflavone (**2**) [51], 3′-methoxyflavone (**3**) [51], 2′,5′-dimethoxyflavone (**4**) [51], 3′-hydroxy-4′,5′-dimethoxyflavone (**5**) [51], 3′-methoxy-4′,5′-methylendioxyflavone (**6**) [51], 4-hydroxyacetophenone (**7**) [27], 4-hydroxy-3-methoxyacetophenone (**8**) [27], 2-hydroxy-4-methoxyacetophenone (paeonol) (**9**) [52], riccardin C (**10**) [27], 8′-oxoriccardin C (**11**) [27], 8′-hydroxyisomarchantin C (**12**) [27], and 8′-hydroxydihydroptyhantol A (**13**) [27]. To the best of our knowledge, flavones **1**–**6** are detected for the first time in the roots of *P. veris*, although they have been previously reported as constituents of the leaves of *P. veris* and other *Primula* species [1,50,51]. Paeonol (**9**) is also described for the first time in the roots of *P. veris*. It has been recently detected as one of the major volatile components in *P. vulgaris* Huds. subsp. *vulgaris* [53]. Acetophenones **7** and **8** and bisbibenzyls **10**–**13** have been recently isolated from Serbian *P. veris* subsp. *columnae* [27].

The methanol extract was subjected to polyamide column chromatography to give three main fractions, A–C. The subsequent separation of fraction B led to the isolation of two pairs of closely related compounds. A comparison of their ^1^H NMR data (Supplementary Part I.) with those published in the literature allowed for the identification of the phenolic glycosides primulaverin (**14**) [10], primeverin (**15**) [10], 2-primeverosyl-5-methoxy-acetophenone (**16**) [54], and paeonolide (paeonol 2-O-β-xylopyranosyl (1→6)-β-glucopyranoside, **17**) [54,55]. Compounds **14**/**15** and **16**/**17** were detected in ratios of 1:0.07 and 1:0.2, respectively. Primulaverin (**14**) and primeverin (**15**) are characteristic compounds of *P. veris* roots, and the content of primulaverin is usually higher than that of primeverin [8,9,10,56]. These compounds degrade over storage time through the activity of the enzyme primverase and produce a typical odor which may serve as an indicator of the age of the plant material [56]. It is worth mentioning that 2-primeverosyl-5-methoxy-acetophenone (**16**) and paeonolide (**17**) are reported here for the first time in *P. veris*. These compounds have been previously reported as constituents of *P. maximowiczii* of Chinese origin [54].

**Figure 2:**
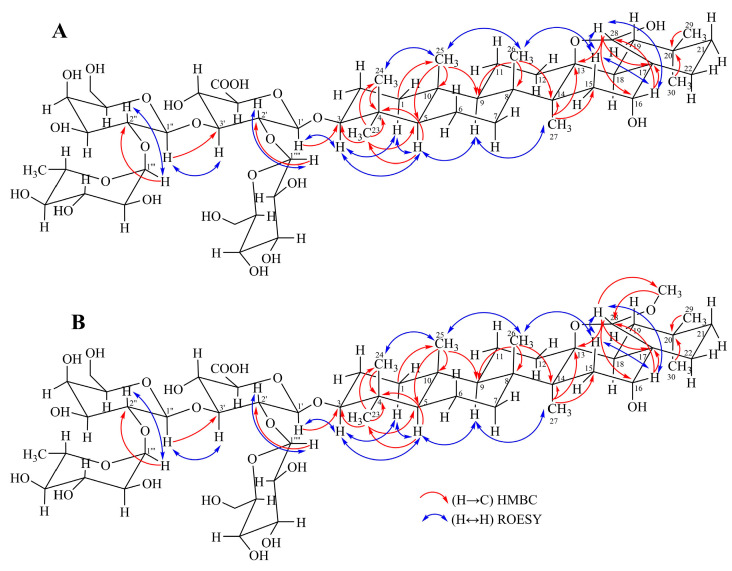
Important HMBC and ROESY correlations for compounds **19** (**A**) and **20** (**B**).

Column chromatography of fraction C and further purification resulted in the isolation of the known primulasaponin I (primulic acid I) (**18**) [20] and three undescribed previously compounds, named primulasaponins III–V (**19**–**21**). Their structures were determined using NMR techniques (^1^H, ^13^C, COSY, HSQC, HMBC, and ROESY) and mass spectrometry (HR-ESI-MS) (Supplementary Part II). The NMR data of compound **19** (Table 1) showed the presence of seven signals corresponding to the tertiary methyls [δ_H/C_ 0.87/16.8 (H/C-24), 0.90/16.8 (H/C-25), 0.92/25.0 (H/C-30), 0.93/33.9 (H/C-29), 1.06/28.3 (H/C-23), 1.18/18.4 (H/C-26), and 1.22/19.9 (H/C-27)], ten signals corresponding to the methylene groups [δ_H_ 0.99 (m) and 1.74 (m)/δ_C_ 40.3 (C-1), δ_H_ 1.75 (m) and 2.02 (m)/δ_C_ 27.1 (C-2), δ_H_ 1.44 (m) and 1.50 (m)/δ_C_ 18.7 (C-6), δ_H_ 1.20 (m) and 1.56 (m)/δ_C_ 35.2 (C-7), δ_H_ 1.45 (m) and 1.66 (m)/δ_C_ 19.9 (C-11), δ_H_ 1.28 (m) and 2.03 (m)/δ_C_ 33.6 (C-12), δ_H_ 1.20 (m) and 1.99 (m)/δ_C_ 37.0 (C-15), δ_H_ 1.19 (m) and 2.37 (dd, 12.1 and 14.5 Hz)/δ_C_ 39.8 (C-19), δ_H_ 1.15 (m) and 2.06 (m)/δ_C_ 37.5 (C-21), δ_H_ 1.42 (m) and 1.94 (m)/δ_C_ 27.2 (C-22)], three methine signals [δ_H_ 0.72 (dd, 1.8 and 11.5 Hz/δ_C_ 56.8 (C-5), δ_H_ 1.22 (m)/δ_C_ 51.4 (C-9) and δ_H_ 1.67 (m)/δ_C_ 47.6 (C-18)], and three oxygenated methine signals [δ_H_ 3.20 (dd, 4.0 and 11.3 Hz)/δ_C_ 92.1 (C-3), δ_H_ 3.78 (m)/δ_C_ 77.3 (C-16) and δ_H_ 4.60 (s)/δ_C_ 99.7 (C-28)]. In addition, the positions of seven quaternary carbons were determined from the observed HMBC correlations (Figure 2) as follows: H-5, H-23, and H-24 with C-4 (δ_C_ 40.7); H-5 and H-25 with C-10 (δ_C_ 37.8); H-26 with C-8 (δ_C_ 43.34); H-26 and H-27 with C-14 (δ_C_ 43.9); H-27 and H-28 with C-13 (δ_C_ 88.9); H-16 and H-28 with C-17 (δ_C_ 48.7); and H-29 and H-20 with C-20 (δ_C_ 32.4). Furthermore, four glycosyl units were determined as glucuronyl [δ_H_ 4.45 (d, 7.7 Hz)/δ_C_ 105.8 (C-1′)], galactosyl [δ_H_ 5.20 (d, 7.8 Hz)/δ_C_ 100.8 (C-1″)], rhamnosyl [δ_H_ 5.28 (d, 1.2 Hz)/δ_C_ 102.0 (C-1‴)], and glucosyl [δ_H_ 4.88/δ_C_ 102.5 (C-1⁗)]. The coupling constants (Table 1) revealed β-configuration for glucuronic acid, galactose, and glucose and α-configuration for rhamnose. In addition, the HMBC correlation between H-3 and an anomeric carbon at δ_C_ 105.75 (C-1′) indicated that the glycosyl part is attached at C-3 (Figure 2), while HMBC correlations - H-1″/C-3′ (δ_C_ 81.2), H-1‴/C-2″ (δ_C_ 76.2), and H-1⁗/C-2′ (δ_C_ 79.3) determined the linkage of the glycosyl units. The comparison of these data with those for primulasaponin I (**18**) showed the same tetrasaccharide moiety at C-3 and almost the same triterpene framework. The only difference was the presence of an additional hydroxy group at C-28, which was confirmed by the HMBC correlations H-28/C-13, H-28/C-16, and H-28/C-17. Additionally, the HR-ESI-MS of **19** displayed a deprotonated molecular ion peak [M−H]^−^ at *m*/*z* 1119.55784 (calcd. 1119.55928), which corresponds to a compound with a molecular formula of C_54_H_88_O_24_, and differed from that of **18** with 16 Da ([M−H]^−^ at *m*/*z* 1103.56257, Figure SII-9). The relative stereochemistry at C-3, C-16, and C-28 was determined using ROESY experiment (Figure 2A). Thus, the observed correlations H-3/H-5 and H-5/H-9 revealed their α-axial orientation (i.e., β-orientation of the oxygen function at C-3), while the correlations H-25/H-26 and H-26/H-15 (δ_H_ 1.99) indicated that they were β-orientated. In addition, the correlations H-15 (δ_H_ 1.99)/H-28 and H-16/H-28 confirmed the α-position of the hydroxyl groups at C-16 and C-28. Therefore, compound **19** was identified as 3-O-β-(β-D-glucopyranosyl-(1→2)-[α-L-rhamnopyranosyl-(1→2)-β-D-galactopyranosyl]-(1→3)-β-D-glucuronopyranosyl)-16α,28α-dihydroxy-13β,28-epoxy-oleanane, named primulasaponin III.

Compound **20** had a molecular formula of C_55_H_90_O_24_, determined based on its deprotonated molecular ion peak [M−H]^–^ (*m*/*z* 1133.57382, calcd. 1133.57493). Extensive 1D and 2D NMR analysis showed that compound **20** differed from **19** only in the aglycone part. Thus, the signal for H-28 in **19** was shifted upfield at δ_H_ 4.17 (s) and that for C-28 was shifteddownfield at δ_C_ 105.80 (Table 1). Furthermore, an additional signal for the methoxy group appeared at δ_H/C_ 3.31 (s)/55.36. The location of the latter at C-28 was deduced from the HMBC correlation between the C-28 and the methyl of a methoxy group at δ_H_ 3.31 as well as between H-28 and the carbon of the methoxy group at δ_C_ 55.4 (Figure 2B). Furthermore, the observed correlations H-25/H-26 and H-26/H-15 (δ_H_ 1.99) in the ROESY experiment indicated their β-orientation, while the correlations H-15 (δ_H_ 1.99)/H-28 and H-16/H-28 confirmed the α-position of the C-16 hydroxy and C-28 methoxy groups. Therefore, compound **20** was identified as 3-O-β-(β-D-glucopyranosyl-(1→2)-[α-L-rhamnopyranosyl-(1→2)-β-D-galactopyranosyl]-(1→3)-β-D-glucuronopyranosyl)-16α-hydroxy-28-methoxy-13β,28-epoxy-oleanane, named primulasaponin IV.

The HR-ESI-MS of compound **21** displayed a deprotonated molecular ion peak [M−H]^−^ at *m*/*z* 1117.54210 (calcd. 1117.54363), which corresponds to a compound with a molecular formula of C_54_H_86_O_24_. The NMR data (Table 1) of **21** were very similar to those of compound **19** and showed a difference only in the aglycone part, namely at C-28. Thus, an additional signal at δ_C_ 182.02 appeared instead of the signals for an oxymethine group at δ_H_ 4.60 (s)/δ_C_ 99.7 (C-28). The presence of a carbonyl group was also confirmed by the downfield shift of C-13 (δ_C_ 94.9). Thus, the structure of compound **21** was elucidated as 3-O-β-(β-D-glucopyranosyl-(1→2)-[α-L-rhamnopyranosyl-(1→2)-β-D-galactopyranosyl]-(1→3)-β-D-glucuronopyranosyl)-16α-hydroxy-oleanan-28,13β-olide, named primulasaponin V.

Finally, the monosaccharides of compounds **18**–**21** obtained by acidic hydrolysis of a portion of the saponin-containing fraction C were identified as D-glucose, D-galactose, D-glucuronic acid, and L-rhamnose via TLC comparison with authentic standards.

The new saponins **19**–**21** could be described as oxygenated derivatives of primulasaponin I (**18**), differing in the nature of the substituent at C-28. It seems that oleanane triterpenoids with the 16α-hydroxy and 13β, 28-epoxy ring are characteristic for *Primula* species [57], while C-28 carbonyl derivatives (28→13 lactones) like compound **21** have not been found in Primulaceae species so far. The studied population of *P. veris* showed a different chemical profile from those reported previously for *P. veris*, *P. veris* subsp. *macrocalyx*, *P. veris* subsp. *veris*, and commercially available root extracts of *Primula* sp. [9,10,20,21,22,23]. Primulasaponin I (**18**) was found in the roots of all studied plants excluding *P. veris* subsp. *macrocalyx* [22]. It would be speculative to believe that intraspecific differences lead to different compositions, especially since some of the studies so far lack precise data on the taxonomic identity of the species. Moreover, environmental and ecological factors could also contribute to the differences in the main chemical components of plants.

### 2.2. Minimum Inhibitory Concentration (MIC) and Minimum Bactericidal Concentration (MBC) of Primula veris Extracts and the Saponin-Enriched Fraction

In our study, we used four bacterial strains responsible for infections in the respiratory tract, the urogenital tract, skin, and oral cavity, as well as wound infections and implant-associated infections [34,58]. To determine the MIC of the methanol (ME) and chloroform (CE) extracts from *Primula veris* roots, as well as the saponin-enriched fraction (SF), the oxidation–reduction indicator resazurin (7-hydroxy-3H-phenoxazin-3-one 10-oxide) was used. This is a blue dye that transforms into resorufin upon changes in the metabolic activity of bacterial cells. It is known that this indicator dye is suitable for antibacterial screening of plant extracts and phytochemicals [59,60], essential oils [61], biosurfactants, and others. Data for the determination of the MIC and MBC are summarized in Table 2. The microdilution test showed that for both Gram-negative strains, MIC varied from 1 to 0.5 mg/mL for all three tested extracts. The lowest antimicrobial activity was recorded against *P. aeruginosa* after treatment with the methanol extract and the saponin fraction. For *E. coli*, all three tested extracts showed a MIC of 0.5 mg/mL. Among the Gram-positive strains, the lowest MIC of 0.5 mg/mL was observed for the saponin-enriched fraction. In comparison with the literature data, some differences in MIC values were observed in extracts from other *Primula* species.

Studies on the methanol extract from *P. auriculata* leaves reported antimicrobial activity at concentrations of 1 mg/mL against *S. aureus* and lower values of 0.5 mg/mL against *E. coli* [43]. After a broader study on five *Primula* species, namely *P. denticulata*, *P. elliptica*, *P. macrophylla*, *P. rosea*, and *P. stuarti*, it was found that MIC values of methanol extracts with a range from 0.5 mg/mL to 0.125 mg/mL depending on the species inhibited *S. aureus* [62]. A study on plant extracts from the roots of *P. vulgaris* subspecies *rubra* reported identical MIC values of 1.25 mg/mL for all three tested strains—*S. aureus*, *E. coli*, and *P. aeruginosa*. A difference was observed only in *P. spina-christi* for root extracts, where MIC values for *S. aureus* were determined at 0.625 mg/mL, while for the two Gram-negative strains, they remained within the same range as *P. vulgaris* at 1.25 mg/mL [35].

### 2.3. Inhibitory Effects of Sub-MICs of Primula veris Extracts and the Saponin-Enriched Fraction on Biofilm Formation

The inhibitory effects of the sub-MICs of ME, CE, and SF on biofilm formation were compared. The crystal violet staining method, which is widely used for biofilm analysis, was applied. The concentration-dependent antibiofilm effects were examined over a 24 h treatment period during biofilm formation and compared against an untreated control sample.

**Figure 3:**
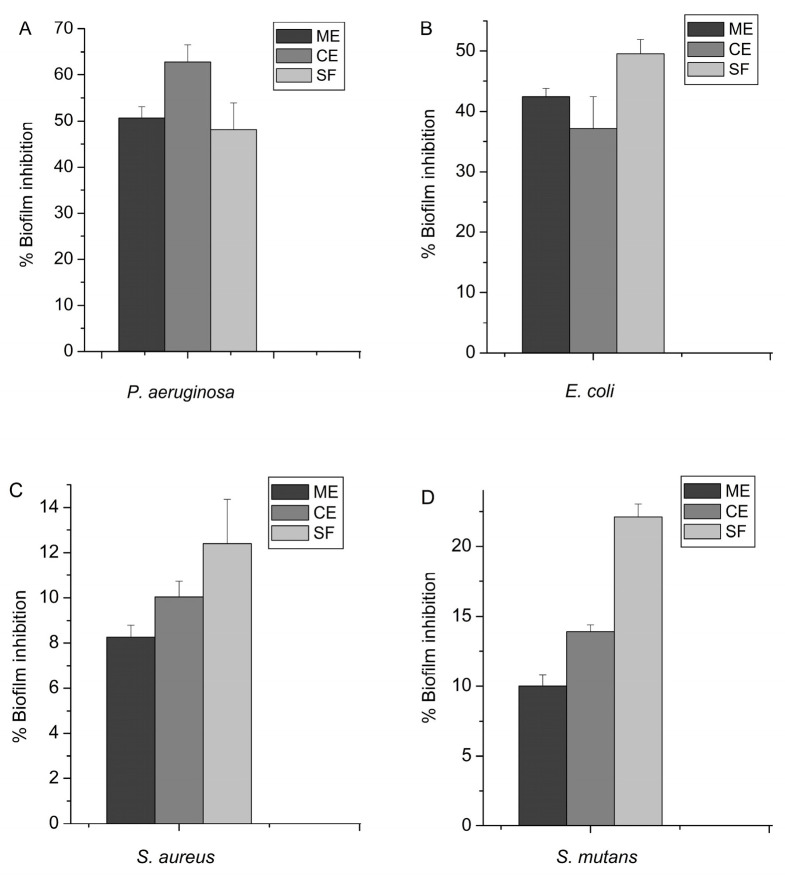
Inhibition of biofilm formation under the influence of methanol (ME) and chloroform (CE) extracts of *P. veris* roots and the saponin-enriched fraction (SF). The antibiofilm effects were examined after a 24 h treatment period and calculated as a percentage of the biofilm in the untreated control sample. (**A**) *P. aeruginosa*; (**B**) *E. coli*; (**C**) *S. aureus*; (**D**) *S. mutans*.

The results, illustrating the percentage of biofilm inhibition by the applied samples, are shown in Figure 3. The data revealed a clear trend of higher inhibition rates in Gram-negative strains compared to Gram-positive strains, where the highest inhibition by the saponin-enriched fraction barely reached 22%. As can be seen from Figure 3, all three tested 1/2 sub-MICs showed a significant inhibition percentage of *P. aeruginosa*‘s biofilm, with the highest effect recorded for the chloroform extract (62.8%), followed by the methanol extract (50.6%) and the saponin-enriched fraction (48.1%). In contrast, the SF (49.6%) and ME (42.4%) demonstrated better antibiofilm effects on *E. coli* than that of CE (37.1%). The observed effect for the two Gram-positive strains confirmed a general trend, with the highest inhibition percentage found in the saponin-enriched fraction and the lowest in the methanol extract. Nonetheless, an inhibition of over 10% was recorded in four of the tested 1/2 MICs for both Gram-positive strains.

Data on the effects of sub-minimal inhibitory concentrations of plant extracts on biofilm formation have also been reported by other authors [63,64,65]. But in comparison with our findings, we identified only one study reporting the antibiofilm activity of *P. vulgaris* root extract against *C. albicans*, an opportunistic pathogen responsible for vaginal candidiasis, with an MIC of 156 µg/mL [35]. The observed differences in the activity of the different extracts are not surprising as the extracts contained different types of compounds, which in turn influences their ability to inhibit biofilm formation. Similar results have been already reported in the literature for *Myrsine umbellata* (Primulaceae) [66], *Cyclamen hederiflolium* (Primulaceae) [67], *Etlingera elatior* [68], *Inula* sp. [69], etc. It is known that some phytochemicals, besides acting as potential biofilm inhibitors, can also suppress quorum-sensing signaling pathways, bioluminescence, and pigment production [69,70]. In certain cases, biofilm formation may have a protective role [71]. Regarding the flavonoid group, there is evidence of the group’s antibiofilm potential against pathogens such as *S. aureus*, a causative agent of infections in implantable devices, where targeted inhibition of Bap-mediated biofilm formation leads to a reduction in catheter colonization [72]. The antibiofilm and anti-amyloid activity of various flavonoids has also been demonstrated in *S. mutans* and *E. coli* [73,74]. Saponin-rich extracts and pure saponins are also reported to possess good antibiofilm activity against *P. aeruginosa*, *E. coli*, and *S. aureus* [67,75,76,77,78]. There are several hypotheses regarding the inhibitory properties of plant phytochemicals on biofilms. First, they penetrate the biofilm matrix by forming micropores and channels, allowing interaction with bacterial cell wall proteins and leading to its disruption, increased permeability, and the accumulation of the treatment compound in the cytoplasm. In some cases, cell lysis may occur, resulting in the leakage of intracellular components and subsequent cell death. Simultaneously, the adhesion potential of the cell is also disrupted. Phytochemicals can damage DNA and RNA structures and inhibit various enzymes involved in replication, transcription, and translation processes. As a result, gene expression, intracellular metabolism, and cell proliferation are inhibited. Their impact on translation leads to impaired synthesis of EPS, QS molecules, virulence factors, and motility structures. Moreover, efflux pumps are affected, and proton gradients are disrupted. Along with the formation of micropores, this facilitates the penetration of various antibiofilm agents [79,80]. Considering the above, we assume that the higher antibiofilm effectiveness of the plant extracts tested against Gram-negative bacteria may be due to two main reasons. On the one hand, their impact on biofilm cells is influenced by the composition of the bacterial cell wall. In Gram-positive bacteria, the cell wall consists mainly of a thick peptidoglycan layer, which may hinder the action of plant molecules and their access to the cell. In contrast, in Gram-negative bacteria, we assume that the penetration of treatment compounds into the cell is facilitated by the thinner peptidoglycan layer. Additionally, it is possible that a mechanism is activated to specifically inhibit certain pathways involved in exopolysaccharide matrix production, leading to its suppression and, consequently, the inability to form a biofilm, a phenomenon also confirmed by other authors [58,81]. In summary, the strategy of biofilm suppression using plant extracts appears promising. It could reduce reliance on conventional antibiotics and consequently limit antibiotic resistance.

### 2.4. Cytotoxicity Against Human Keratinocyte Cell Line HaCaT

**Figure 4:**
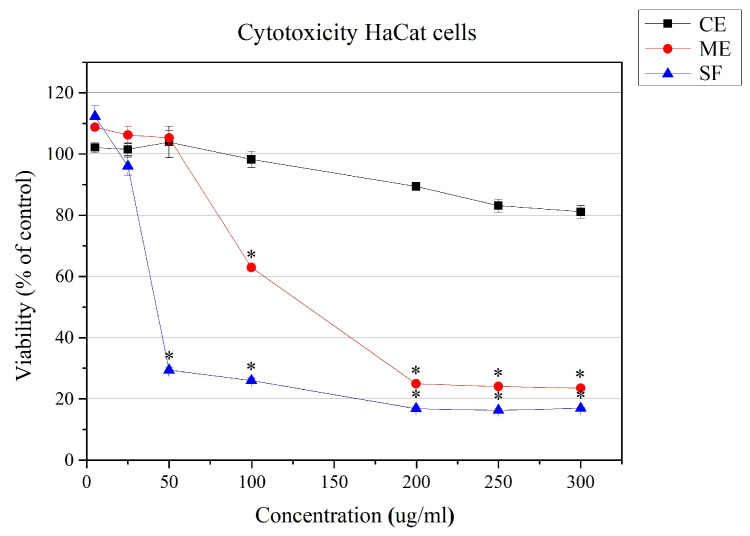
Inhibition of cellular viability under the influence of the methanol (ME) and chloroform (CE) extracts of *P. veris* roots and the saponin-enriched fraction (SF). Cytotoxicity was examined at after a 24 h treatment period and calculated as a percentage of the untreated control cells (* *p* < 0.05).

The cytotoxicity assessment of the methanol (ME) and chloroform (CE) extracts of *P. veris* roots and the saponin-enriched fraction (SF) on a human keratinocyte cell line was performed using the CV test (crystal violet staining) to avoid the influence of enzyme-based cytotoxicity assays due to a reaction with polyphenols [82]. Our results showed the expected high cytotoxicity of the saponin-enriched fraction (SF), which is caused by the interaction of saponins with cell membranes [83]. The methanol extract also showed relatively high activity, while the chloroform extract was not toxic in the tested concentration range up to 300 μg/mL, and cell survival was above 80% (Figure 4). Combined with the high antibiofilm activity, this makes the chloroform extract of *P. veris* roots a promising subject for future research.

The effect of saponins in ethnomedicine is well known, and their action at the organism level has been studied to some extent [84]. Little is known about their action in vitro at the cellular level. There is evidence that saponins interact with cell membranes, causing temporary or permanent micropores in tumor and actively dividing cells, thus causing cell cycle arrest, disturbances in ionic balance and/or apoptosis in actively dividing cells, and they are potential candidates for application in antitumor therapies. There is evidence of specific effects on specific stress-associated signaling pathways and the activation of apoptosis in myeloid leukemia and other tumor cells by triterpenoid saponins and saikosaponin [85]. When applied externally, their contact with highly differentiated and non-dividing keratinocytes will not have a negative effect, while the development of melanomas or other skin diseases associated with increased proliferation and inflammation, such as psoriasis, for example, as well as wound healing, would be well influenced [4].

## 3. Materials and Methods

### 3.1. General Experimental Procedures

Specific optical rotation values were measured on a Jasco P-2000 polarimeter (Jasco, Tokyo, Japan) at the D line of a sodium lamp at 20 °C by using a 0.5 dm quartz cell. The [α]$\begin{array}{c} 20\\ D \end{array}$ is given in deg·cm^3^·g^−1^·dm^−1^ and concentration (c) in g·cm^−3^. The 1D and 2D NMR (^1^H and ^13^C NMR, DEPT, COSY, HSQC, HMBC, and ROESY) spectra were recorded on a Bruker Avance NEO 600 spectrometer (Biospin GmbH, Rheinstetten, Germany) with the operating frequencies at 600 MHz (^1^H) and 150 MHz (^13^C) using the residual solvent’s signals (δ_H_ 7.26 in ^1^H and δ_C_ 77.00 ppm in ^13^C for CDCl_3_ and δ_H_ 3.31 in ^1^H and δ_C_ 49.3 ppm in ^13^C for CD_3_OD) as a reference. The chemical shifts (δ) are expressed in ppm and coupling constants (*J*) in Hz. HRESIMS spectra were acquired in negative mode on the Q Exactive Plus (Thermo Fisher Scientific, Inc., Bremen, Germany) mass spectrometer equipped with a heated HESI-II source. IR spectra were recorded on a Shimadzu IR Spirit FT-IR spectrometer using QATR-S as a single-reflection ATR measurement attachment.

Polyamide (Carl Roth GmbH + Co. KG, Karlsruhe, Germany), Silica gel 60 (70-230 mesh ASTM) (Merck KGaA, Darmstadt, Germany), and LiChroprep^®^ RP-18 (40–63 µm) (Merck KGaA, Darmstadt, Germany) were used as adsorbents for column chromatography (CC). MPLC was performed on LiChroprep^®^ RP-8 (Merck, Darmstadt, Germany). Thin-layer chromatography (TLC) on Silica gel 60 F_254_ (Merck, Darmstadt, Germany) and Silica gel RP-18 (Merck, Darmstadt, Germany) plates was used to monitor the separation of the extracts and for preparative TLC. The spots were visualized by spraying with concentrated H_2_SO_4_or with the NP reagent (1% diphenylboronic acid 2-aminoethyl ester in ethyl acetate), followed by heating at 105 °C. All solvents used were of HPLC grade.

### 3.2. Plant Material

The roots of *Primula veris* L. were collected in April 2023 in the vicinity of Assenovgrad town, Bulgaria (GPS 42.014051; 24.855882), at an altitude of 276 m. A voucher specimen (SOM 179 373) was deposited in the herbarium of the Institute of Biodiversity and Ecosystem research, Bulgarian Academy of Sciences, Sofia, Bulgaria. The plant was identified by Assoc. Prof. Dr Vladimir Vladimirov from the Institute of Biodiversity and Ecosystem Research, Bulgarian Academy of Sciences, Sofia, Bulgaria.

### 3.3. Extraction and Isolation of Individual Compounds

The air-dried and finely powdered roots of *P. veris* (52 g) were sequentially extracted with CHCl_3_ and MeOH (3 × 500 mL) at room temperature for 24 h each. The extracts were filtered and concentrated under vacuum using a rotary evaporator to obtain the corresponding CHCl_3_ (0.328 g) and MeOH (3.98 g) extracts.

A portion of the CHCl_3_ extract (0.200 g) was subjected to CC on the silica gel using the *n*-hexane/ethyl acetate mixture with increasing polarity (from 10:1 to 0:1). Fourteen fractions (F_1_–F_14_) were collected via TLC monitoring (Silica gel, n-hexane/ethyl acetate, 3:1 and RP-18 MeOH/H_2_O 8:2). Prep. TLC (Silica gel, n-hexane/diethyl ether, 2:1, twice development) of fr. F_3_ (12 mg) afforded compound **9** (1.2 mg). Prep. TLC (Silica gel, n-hexane/diethyl ether, 2:1) of fr. F_5_ (8.0 mg) and F_7_ (5.0 mg) yielded compounds **1** (5.3 mg) and **3** (3.0 mg). Prep. TLC (RP-18, H_2_O/MeOH, 2:8) of fr. F_9_ (6 mg) gave compound **2** (1.3 mg) and a mixture of compounds **7** and **8** (0.9 mg and a ratio of 0.3:1, as deduced from ^1^H NMR). Prep. TLC (RP-18, H_2_O/MeOH, 2:8) of fr. F_11_ (13 mg) afforded 4.6 mg of compound **10**, 1.4 mg of compound **12**, and a mixture of compounds **4** and **6** (0.7 mg and a ratio of 1:0.5, as deduced from ^1^H NMR). Prep. TLC (RP-18, H_2_O/MeOH, 2:8) of fr. F_13_ (10 mg) led to the isolation of compounds **11** (3.8 mg), **5** (1.1 mg), and **13** (0.9 mg).

A portion of the methanolic extract (3.0 g) was separated by CC on Polyamide using H_2_O/MeOH mixtures (from 0:1 to 1:0) to give 3 fractions A-C. MPLC on LiChroprep RP-8 of a portion of fr. B (60 mg) with H_2_O/MeOH (0:1 to 0:1) afforded a mixture of compounds **14** and **15** (23.6 mg in a ratio of 1:0.07, as deduced from ^1^H NMR) and afforded a mixture of compounds **16** and **17** (12.6 mg in a ratio of 1:0.2, as deduced from ^1^H NMR). Fr. C (360 mg) was subjected to MPLC on LiChroprep RP-8 (H_2_O/MeOH, 1:1 to 0:1) to give 7 subfractions C_1_–C_7_. MPLC on LiChroprep RP-8 (H_2_O/MeOH, 4:6 to 0:1) of fr. C_2_ afforded 8.12 mg of **19**. Prep. TLC (RP-18, H_2_O/MeOH, 4:6) of fr. C_3_ afforded 5.2 mg of **21**. MPLC on LiChroprep RP-8 (H_2_O/MeOH, 4:6 to 0:1) of fr. C_5_ yielded 25.2 mg of **18**. MPLC on LiChroprep RP-8 (H_2_O/MeOH, 4:6 to 0:1) of fr. C_6_ gave 5.2 mg of **18** and 7.6 mg of **20**.

***Primulasaponin III* (19)**: Amorphous white powder, [α]$\begin{array}{c} 20\\ D \end{array}$ −0.09 (c 0.6, MeOH); FT-IR (ATR): ν_max_ 3352, 2947, 1604, and 1037 cm^−1^; ^1^H (600 MHz, CD_3_OD) and ^13^C (150 MHz, CD_3_OD): Table 1; HR-ESI-MS *m*/*z*: 1119.55784 [M−H]^−^ (calcd. for C_54_H_87_O_24_ 1119.55928).

***Primulasaponin IV* (20)**: Amorphous white powder, [α]$\begin{array}{c} 20\\ D \end{array}$ −0.01 (c 0.154, MeOH); FT-IR (ATR): ν_max_ 3330, 2921, 1609, and 1043 cm^−1^; ^1^H (600 MHz, CD_3_OD) and ^13^C (150 MHz, CD_3_OD): Table 1; HR-ESI-MS *m*/*z*: 1133.57382 [M−H]^−^ (calcd. for C_55_H_89_O_24_ 1133.57493).

***Primulasaponin V* (21)**: Amorphous white powder, [α]$\begin{array}{c} 20\\ D \end{array}$ −0.07 (c 0.375, MeOH); FT-IR (ATR): ν_max_ 3350, 2924, 1744, 1604, and 1040 cm^−1^; ^1^H (600 MHz, CD_3_OD) and ^13^C (150 MHz, CD_3_OD): Table 1; HR-ESI-MS *m*/*z*: 1117.54210 [M−H]^–^ (calcd. for C_54_H_85_O_24_ 1117.54363).

### 3.4. Hydrolysis of Saponin-Enriched Fraction

A portion of fraction C (SF) (10 mg) that was dissolved in 1 mL of MeOH was mixed with 5% HCl (5 mL) and refluxed for 4 h. The reaction mixture was concentrated under reduced pressure to remove MeOH. After extraction with CH_2_Cl_2_, the aqueous solution was concentrated under vacuum to obtain the sugar residue and compared with authentic standards (D-glucose, D-galactose, D-glucuronic acid, and L-rhamnose) by TLC (Silica gel, CH_3_CN/H_2_O, 85:15).

### 3.5. Bacterial Strains, Growth Medium, and Cultural Conditions

This study included two Gram-negative and two Gram-positive bacterial strains. *Pseudomonas aeruginosa* 15692 (ATCC) is a strain from the International Reference Panel [86] isolated from wounds, *Escherichia coli* 25922 (ATCC), *Staphylococcus aureus* 29213 (ATCC), and *Streptococcus mutans* 35668 (ATCC). For long-term storage, the strains were frozen at −80 °C with the addition of the cryoprotectant 8% DMSO. Before experimental work, each strain was inoculated in a growth medium according to its specific requirements: *P. aeruginosa* and *S. aureus* in tryptic soy broth (TSA, Sigma, Burlington, MA, USA), *E. coli* in nutrient broth (HiMedia, Bedford, PA, USA), and *S. mutans* in brain heart infusion (HiMedia, Bedford, PA, USA).

### 3.6. Evaluation of the Minimum Inhibitory Concentration (MIC) and the Minimum Bactericidal Concentration (MBC)

For the evaluation of MIC and MBC, standard culture media made of Mueller–Hinton broth (MHB) and Mueller–Hinton agar (MHA) (HiMedia, Bedford, PA, USA) were used. MIC was tested using the widely applied microdilution method, which relies on the oxidation–reduction indicator resazurin to assess changes in the metabolic activity of bacterial cells [59]. For the experiment, the test substances were diluted over a wide concentration range, from 2 mg/mL to 0.0156 mg/mL, with the addition of DMSO at a final concentration of 2% to avoid toxicity to bacterial cells. The initial concentration of bacterial strains was applied after densitometric measurement following the McFarland standard to a final cell concentration of 1 × 10^6^ CFU/mL. Aliquots of 100 µL of the tested extracts (chloroform, methanol, and saponin fraction) at concentrations of 2 mg/mL were added to the first column of the plate, followed by serial two-fold dilutions in the MHB medium to the final test concentration. To each well, 10 µL of a 0.01% sterile aqueous resazurin solution (Sigma, New York, NY, USA) and 10 µL of bacterial suspension were added. The plates were incubated for 24 h at 37 °C under static conditions. As a positive control, 10 µL of ciprofloxacin (Sigma, New York, NY, USA) was added, while a sterile nutrient medium served as the negative control. After the incubation period, the lowest concentration of the test substance where a visual color change from blue to pink was observed was recorded as the minimum inhibitory concentration (MIC). To determine the MBC, samples were taken from the wells of the plate using a sterile swab and plated onto an MHA medium. The lowest concentration of the substances where no bacterial growth was observed was considered the MBC.

### 3.7. Biofilm Formation Experiments

To determine the antibiofilm effect of the applied plant extracts, the bacterial strains were cultivated in minimal salt medium M63 with the following composition: KH_2_PO_4_ (0.02 M), K_2_HPO_4_ (0.02 M), (NH_4_)_2_SO_4_ (0.02 M), MgSO_4_ (0.1 mM), and glucose (0.04 M), with a pH of 7.5. As a source of bacterial inoculum, the strains were incubated in the media described above for 18 h at 37 °C. The cultures were then diluted at a ratio of 1:100 in M63, and the plant extracts were applied in the presence of sub-MICs (1/2 MIC). The prepared suspensions were pipetted into 96-well U-bottomed polystyrene microtiter plates (Corning, Corning, NY, USA) with a final volume of 150 μL per well in six replicates per sample. The control sample contained only bacterial inoculum diluted in M63 with the addition of 2% DMSO (untreated probe). The prepared plates were incubated for 24 h at 37 °C under static conditions. After incubation, the non-adherent planktonic cells were removed by washing the wells with phosphate-buffered saline (PBS, pH 7.2), and the attached cells were stained with a 0.1% aqueous solution of crystal violet for 15 min. The final step involved extracting the dye with 70% ethanol for Gram-negative bacteria and a 95% ethanol–acetone (4:1) dilution for Gram-positive bacteria. Absorbance was measured at a wavelength of 595 nm using an ELISA plate reader (LTEK INNO, Gyeonggi-do, Republic of Korea). The quantitative data were analyzed as the means ± standard deviation (SD) using OriginPro 6.1 software.

### 3.8. Cytotoxicity Assessment

The cytotoxicity assessment of methanol (ME) and chloroform (CE) extracts from *P. veris* roots and the saponin-enriched fraction (SF) on a human keratinocyte cell line was performed via the CV assay (crystal violet staining) as previously described [87]. All experiments were performed in triplicate. The optical density of the samples was measured at 570 nm using an Epoch Microplate Spectrophotometer (BioTek^®^ Instruments Inc., Winooski, VT, USA) with Gen5™ Data Analysis software, version 1.11.5. The results are presented as a percentage of cell viability compared to the control of untreated cells. The data were analyzed with OriginPro 9.0 and presented as a mean value ± SE. Statistical significance is according to one-way ANOVA at the 0.05 level (* *p* < 0.05).

## 4. Conclusions

In summary, the current study showed the presence of various phenolic compounds (flavones, acetophenones, phenolic glycosides, and bisbibenzyls) and saponins closely related to primulasaponin I in the roots of *Primula veris* of Bulgarian origin. Among them, three triterpene saponins were found to be new compounds undescribed so far. This investigation contributes to the phytochemical characterization of cowslip in Europe, but detailed phytochemical analyses of more populations of *P. veris* are needed to understand the intraspecific and interspecific variability of this well-known medicinal plant. Additionally, the antibiofilm properties of the chloroform and methanol extracts and the saponin-enriched fraction obtained from the roots of *P. veris* were studied for the first time. The obtained results revealed better biofilm inhibition of the tested samples of Gram-negative bacterial strains (*P. aeruginosa* and *E. coli*) than Gram-positive ones (*S. aureus* and *S. mutans*). Expanding knowledge on biofilm inhibition mechanisms, particularly the modulation of bacterial virulence traits by plant extracts, provides advantages for discovering other agents with similar effects and potential applicability in medical practice for treating biofilm-related infections.

## Figures and Tables

**Table 1:** ^1^H (600 MHz) and ^13^C (150 MHz) NMR data of compounds **19**–**21** in CD_3_OD (δ in ppm, multiplicity, and J in Hz).

Position	19		20		21	
	δ_C_	δ_H_	δ_C_	δ_H_	δ_C_	δ_H_
1	40.3	0.99 m ^1^	40.3	0.98 m	40.2	1.02 m
		1.74 m		1.75 m		1.75 m
2	27.1	1.75 m	27.1	1.75 m	27.0	1.75 m
		2.02 m		1.95 m		2.01 m
3	92.1	3.20 dd (4.0, 11.3)	92.3	3.19 dd (4.0, 11.3)	91.9	3.21 dd (4.0, 11.3)
4	40.7	-	40.7	-	40.7	-
5	56.8	0.72 dd (1.8, 11.5)	56.8	0.73 dd (1.8, 11.5)	56.8	0.76 dd (1.8, 11.5)
6	18.7	1.44 m	18.7	1.43 m	18.7	1.43 m
		1.50 m		1.50 m		1.55 m
7	35.2	1.20 m	35.2	1.22 m	34.9	1.24 m
		1.56 m		1.55 m		1.59 m
8	43.3	-	43.4	-	43.0	-
9	51.4	1.22 m	51.4	1.23 m	50.8	1.34 m
10	37.8	-	37.8	-	37.8	-
11	19.9	1.45 m	19.9	1.45 m	19.4	1.55 m
		1.66 m		1.65 m		1.65 m
12	33.6	1.28 m	33.7	1.28 m	32.2	1.56 m
		2.03 m		2.00 m		2.15 m
13	88.9	-	89.0	-	94.9	-
14	43.9	-	44.0	-	43.0	-
15	37.0	1.20 m	37.4	1.20 m	37.4	1.20 m
		1.99 m		1.99 m		1.99 m
16	77.3	3.78 m	77.2	3.82 m	76.2	3.79 m
17	48.7	-	48.9		49.1	-
18	47.6	1.67 m	48.0	1.68 m	52.6	1.92 m
19	39.8	1.19 m	39.8	1.17 m	39.2	1.30 m
		2.37 dd (12.1, 14.5)		2.28 dd (12.1, 14.5)		2.49 dd (12.1, 14.5)
20	32.4	-	32.4	-	32.2	-
21	37.5	1.15 m	37.1	1.15 m	36.7	1.21 m
		2.06 m		2.01 m		2.09 m
22	27.2	1.42 m	26.5	1.42 m	28.7	1.63 m
		1.94 m		1.61 m		1.83 m
23	28.3	1.06 s	28.2	1.06 s	28.2	1.07 s
24	16.8	0.87 s	16.8	0.87 s	16.7	0.88 s
25	16.8	0.90 s	16.8	0.91 s	16.8	0.91 s
26	18.8	1.18 s	18.9	1.18 s	18.3	1.08 s
27	19.9	1.22 s	19.8	1.21 s	19.7	1.38 s
28	99.7	4.60 brs	106.9	4.17 brs	182.0	-
29	33.9	0.93 s	33.9	0.92 s	33.5	0.96
30	25.0	0.92 s	24.9	0.88 s	24.9	0.92
OCH_3_			55.4	3.31 s		
1′	105.8	4.45 d (7.7)	105.8	4.48 d (7.7)	105.8	4.46 d (7.7
2′	79.3	3.92 dd (7.7, 8.8)	79.1	3.93 dd (7.7, 8.8)	79.3	3.92 dd (7.7, 8.8)
3′	81.2	4.06 t (8.8)	81.0	4.05 t (8.8)	81.2	4.07 t (8.8)
4′	72.2	3.59 m	72.2	3.61 m	72.3	3.59 m
5′	75.9	3.79 m	75.9	3.78 m	75.9	3.79 m
6′	176.6	-		-		
1″	100.8	5.20 d (7.8)	100.9	5.20 d (7.8)	100.8	5.20 d (7.8)
2″	76.2	3.79 m	76.1	3.79 m	76.2	3.79 m
3″	75.9	3.72 m	76.0	3.74 m	76.0	3.74 m
4″	71.9	3.72 m	71.8	3.72 m	71.8	3.72 m
5″	76.9	3.53 m	76.9	3.53 m	76.9	3.53 m
6″	62.7	3.65 dd (4.1, 11.5)	62.8	3.65 dd (4.1, 11.5)	62.7	3.67 dd (4.1, 11.5)
		3.81 m		3.81 m		3.81 m
1‴	102.0	5.28 d (1.2)	102.1	5.28 d (1.2)	102.0	5.29 d (1.2)
2‴	72.6	3.97 dd (1.2, 3.5)	72.6	3.96 dd (1.2, 3.5)	72.6	3.96 dd (1.2, 3.5)
3‴	72.3	3.71 dd (3.5, 9.5)	72.3	3.71 dd (3.5, 9.5)	72.2	3.71 dd (3.5, 9.5)
4‴	73.7	3.44 t (9.5)	73.8	3.45 t (9.5)	73.7	3.43 t (9.5)
5‴	70.3	4.10 dq (9.5, 6.5)	70.2	4.11 dq (9.5, 6.5)	70.2	4.11 dq (9.5, 6.5)
6‴	17.9	1.27 d (6.5)	17.9	1.27 d (6.5)	17.9	1.27 d (6.5)
1⁗	102.5	4.88	102.6	4.88	102.6	4.88
2⁗	76.2	3.22 dd (7.2, 9.5)	76.1	3.22 dd (7.2, 9.5)	76.1	3.22 dd (7.2, 9.5)
Glu-3	77.9	3.35 m	77.9	3.35 m	77.9	3.35 m
4⁗	72.4	3.05 t (9.5)	72.6	3.05 t (9.5)	72.6	3.05 t (9.5)
5⁗	78.1	3.40 m	78.1	3.40 m	78.1	3.40 m
6⁗	63.5	3.54 dd (8.0, 12.5)	63.5	3.54 dd (8.0, 12.5)	63.5	3.55 dd (8.0, 12.5)
		3.87 dd (2.5, 12.5)		3.87 dd (2.5, 12.5)		3.88 dd (2.5, 12.5)

^1^ Multiplicity: m—multiplet; d—doublet; dd—doublet of doublets; t—triplet; s—singlet; brs—broad singlets.

**Table 2:** Determination of the MIC and MBC (mg/mL) of *P. veris* extracts and the saponin-enriched fraction.

Sample ^1^	*P. aeruginosa*		*E. coli*		*S. aureus*		*S. mutans*	
	MIC	MBC	MIC	MBC	MIC	MBC	MIC	MBC
CE	1.0	2.0	0.5	0.5	1.0	2.0	1.0	2.0
ME	0.5	1.0	0.5	1.0	1.0	2.0	1.0	2.0
SF	0.5	1.0	0.5	1.0	0.5	2.0	0.5	2.0

^1^ Chloroform extract (CE), methanol extract (ME), and saponin-enriched fraction (SF).

## Data Availability

Data are contained within the article and Supplementary Materials.

## Supplementary Materials

The following supporting information can be downloaded at https://www.mdpi.com/article/10.3390/molecules30081702/s1, Supplementary Part I. Figures SI-1–SI-13: ^1^H NMR spectra of the known compounds **1**–**17**; Supplementary Part II. Figures SII-1–SII-36: Spectral data (^1^H and ^13^C NMR, DEPT, COSY, HSQC, HMBC, ROESY, HR-ESI-MS, and IR) of primulasaponin I (**18**) and new saponins **19**–**21**.

## References

[1] Colombo P.S., Flamini G., Rodondi G., Giuliani C., Santagostini L., Fico G. (2017). Phytochemistry of European *Primula* species. Phytochemistry.

[2] The Plant List *Primula veris* L.. http://www.theplantlist.org/tpl1.1/record/kew-2563733.

[3] Plants of the World Online Kew Science *Primula veris* L.. https://powo.science.kew.org/taxon/urn:lsid:ipni.org:names:702751-1.

[4] Kahraman C., Sari S., Küpeli Akkol E., Tatli Cankaya I. (2022). Bioactive saponins of *Primula vulgaris* Huds. promote wound healing through inhibition of collagenase and elastase enzymes: In vivo, in vitro and in silico evaluations. Chem. Biodivers..

[5] Alam F., Din K.M., Sarfraz M., Qudoos A., Malik S. (2024). Genus *Primula* and its role in phytomedicine; a systematic review. Phytomedicine Plus.

[6] Kemmerich B. (2007). Evaluation of efficacy and tolerability of a fixed combination of dry extracts of *Thyme* herb and *Primrose* root in adults suffering from acute bronchitis with productive cough: A prospective, double-blind, placebo-controlled multicentre clinical trial. Arzneim. Forsch. Drug Res..

[7] Yasar B., Kutlu G., Tornuk F. (2022). Edible Flowers as Sources of Bioactive Compounds: Determination of phenolic extraction conditions. Int. J. Gastron. Food Sci..

[8] Sarropoulou V., Sarrou E., Angeli A., Martens S., Maloupa E., Grigoriadou K. (2023). Species-specific secondary metabolites from *Primula veris* subsp. *veris* obtained in vitro adventitious root cultures: An alternative for sustainable production. Sustainability.

[9] Stefanis I., Chatzopoulou P., Krigas N., Karioti A. (2023). Exploring the chemical content of *Primula veris* L. subsp. *veris* wild-growing populations along a climate gradient: An HPLC-PDA-MS quality assessment of flowers, leaves and roots for sustainable exploitation. Horticulturae.

[10] Müller A., Ganzera M., Stuppner H. (2006). Analysis of phenolic glycosides and saponins in *Primula elatior* and *Primula veris* (*Primula* Root) by liquid chromatography, evaporative light scattering detection and mass spectrometry. J. Chromatogr. A.

[11] Ozkan M.T., Aliyazicioglu R., Demir S., Misir S., Turan I., Yildirmis S., Aliyazicioglu Y. (2017). Phenolic characterisation and antioxidant activity of *Primula vulgaris* and its antigenotoxic effect on fibroblast cells. Jundishapur. J. Nat. Pharm. Prod..

[12] Latypova G.M., Bychenkova M.A., Katayev V.A., Perfilova V.N., Tyurenkov I.N., Mokrousov I.S., Prokofiev I.I., Salikhov S.M., Iksanova G.R. (2019). Composition and cardioprotective effects of *Primula veris* L. solid herbal extract in experimental chronic heart failure. Phytomedicine.

[13] Tünde J., Eleonora M., Laura V., Neagu O., Annamaria P. (2015). Bioactive compounds and antioxidant capacity of *Primula veris L*. flower extracts. Analele Univ. Din Oradea Fasc. Ecotoxicologie Zooteh. De Ind..

[14] Tokalov S.V., Kind B., Wollenweber E., Gutzeit H.O. (2003). Biological effects of epicuticular flavonoids from *Primula denticulata* on human leukemia cells. J. Agric. Food. Chem..

[15] Bhutia T.D., Valant-Vetschera K.M. (2012). Diversification of exudate flavonoid profiles in further *Primula* spp. Nat. Prod. Commun..

[16] Valant-Vetschera K.M., Bhutia T.D., Wollenweber E. (2010). Chemodiversity of exudate flavonoids in *Dionysia* (*Primulaceae*): A comparative study. Phytochemistry.

[17] Valant-Vetschera K.M., Bhutia T.D., Wollenweber E. (2009). Exudate flavonoids of *Primula* spp: Structural and biogenetic chemodiversity. Nat. Prod. Commun..

[18] Bhutia T.D., Valant-Vetschera K.M., Brecker L. (2013). Orphan flavonoids and dihydrochalcones from *Primula* exudates. Nat. Prod. Commun..

[19] Bhutia T.D., Valant-Vetschera K.M., Adlassnig W., Brecker L. (2012). Flavonoids in selected *Primula* spp.: Bridging micromorphology with chemodiversity. Nat. Prod. Commun..

[20] Siems K., Jaensch M., Jakupovic J. (1998). Structures of the two saponins isolated from commercially available root extract of *Primula* sp. Planta Med..

[21] Apel L., Kammerer D.R., Stintzing F.C., Spring O. (2017). Comparative metabolite profiling of triterpenoid saponins and flavonoids in flower color mutations of *Primula veris* L.. Int. J. Mol. Sci..

[22] Çaliş İ., Yürüker A., Rüegger H., Wright A.D., Sticker O. (1992). Triterpene saponins from *Primula veris* subsp. *macrocalyx* and *Primula elatior* subsp. *meyeri*. J. Nat. Prod..

[23] Włodarczyk M., Pasikowski P., Osiewała K., Frankiewicz A., Dryś A., Gleńsk M. (2020). In search of high-yielding and single-compound-yielding plants: New sources of pharmaceutically important saponins from the *Primulaceae* family. Biomolecules.

[24] Herbal Medicinal Product European Medicines Agency (EMA) Primulae Radix. https://www.ema.europa.eu/en/medicines/herbal/primulae-radix.

[25] Kosenkova Y.S., Polovinka M.P., Komarova N.I., Korchagina D.V., Kurochkina N.Y., Cheremushkina V.A., Salakhutdinov N.F. (2007). Riccardin C, a bisbibenzyl compound from *Primula Macrocalyx*. Chem. Nat. Compd..

[26] Bukvicki D., Kovtonyuk N.K., Legin A.A., Keppler B.K., Brecker L., Asakawa Y., Valant-Vetschera K. (2021). Hunting for bis-bibenzyls in *Primula veris* subsp. *macrocalyx* (Bunge) Lüdi: Organ-specific accumulation and cytotoxic activity. Phytochem. Lett..

[27] Novakovic M., Ilic-Tomic T., Djordjevic I., Andjelkovic B., Tesevic V., Milosavljevic S., Asakawa Y. (2023). Bisbibenzyls from Serbian *Primula veris* subsp. *columnae* (Ten.) Lȕdi and *P. acaulis* (L.) L.. Phytochemistry.

[28] Novakovic M., Bukvicki D., Andjelkovic B., Ilic-Tomic T., Veljic M., Tesevic V., Asakawa Y. (2019). Cytotoxic activity of riccardin and perrottetin derivatives from the liverwort *Lunularia cruciata*. J. Nat. Prod..

[29] Xie C.F., Qu J.B., Wu X.Z., Liu N., Ji M., Lou H.X. (2010). Antifungal macrocyclic bis(bibenzyls) from the Chinese liverwort *Ptagiochasm intermedlum* L.. Nat. Prod. Res..

[30] Zafer M.M., Mohamed G.A., Ibrahim S.R.M., Ghosh S., Bornman C., Elfaky M.A. (2024). Biofilm-mediated infections by multidrug-resistant microbes: A comprehensive exploration and forward perspectives. Arch. Microbiol..

[31] Olsen I. (2015). Biofilm-specific antibiotic tolerance and resistance. Eur. J. Clin. Microbiol. Infect. Dis..

[32] Abebe G.M. (2020). The role of bacterial biofilm in antibiotic resistance and food contamination. Int. J. Microbiol..

[33] Ciofu O., Moser C., Jensen P.Ø., Høiby N. (2022). Tolerance and resistance of microbial biofilms. Nat. Rev. Microbiol..

[34] Damyanova T., Paunova-Krasteva T. (2025). What we still don’t know about biofilms—current overview and key research information. Microbiol. Res..

[35] Oyardi O., Hacioglu M., Özdemir E., Erbay M.Ş., Kültür Ş., Bozkurt Güzel C. (2023). Screening of antimicrobial, antibiofilm and cytotoxic activities of some medicinal plants from Balıkesir Province, Türkiye: Pointing to the potential effects of *Allium paniculatum* flower. Turk. J. Pharm. Sci..

[36] Başbülbül G., Özmen A., Biyik H.H., Şen Ö. (2008). Antimitotic and antibacterial effects of the *Primula veris* L. flower extracts. Caryologia.

[37] Karapınar Ç., Öz M. (2023). Chemical content of volatile oil of *Primula veris* subsp. *columnae*, obtaining the methanol extracts and their biological activities. Bioresources.

[38] Ivanišová E., Horňák M., Čech M., Harangozo Ľ., Kačániová M., Grygorieva O., Kowalczewski P.Ł. (2023). Polyphenol content, mineral compounds composition, antimicrobial and antioxidant activities of selected medicinal herbs from Slovak Republic. Appl. Sci..

[39] Chilku E., Ivic Kolevska S., Kadifkova Panovska T. (2017). Antioxidant and antibacterial properties of some commercial plants from Macedonia. World J. Pharm. Pharm. Sci..

[40] Tosun F., Kizilay Ç.A., Şener B., Vural M. (2005). The evaluation of plants from Turkey for in vitro antimycobacterial activity. Pharm. Biol..

[41] Yayli N., Tosun G., Yayli B., Gündoǧan Z., Coşkunçelebi K., Karaoǧlu Ş.A. (2016). Altitude variation in the composition of essential oils, fatty acid methyl esters, and antimicrobial activities of two subspecies of *Primula vulgaris* grown in Turkey. Nat. Prod. Commun..

[42] Majid A., Hassan S., Hussain W., Khan A., Hassan A., Khan A., Khan T., Ahmad T., Rehman M.U. (2014). In vitro approaches of *Primula vulgaris* leaves and roots extraction against human pathogenic bacterial strains. World Appl. Sci. J..

[43] Jaberian H., Piri K., Nazari J. (2013). Phytochemical composition and in vitro antimicrobial and antioxidant activities of some medicinal plants. Food Chem..

[44] Khan S., Shaheen H., Aziz S., Nasar S. (2021). Diversity and distribution of genus *Primula* in Kashmir Region: An indicator genus of the western Himalayan Mountain wetlands and glacial forelands. Biodivers. Conserv..

[45] Najmus-Saqib Q., Alam F., Ahmad M. (2009). Antimicrobial and cytotoxicity activities of the medicinal plant *Primula macrophylla*. J. Enzyme. Inhib. Med. Chem..

[46] Peev D., Velchev V. (1982). Flora of the Republic of Bulgaria.

[47] Assyov B., Petrova A., Dimitrov D., Vassilev P., Assyov B., Petrova A. (2012). Conspectus of the Bulgarian Vascular Flora. Distribution Maps and Floristic Elements.

[48] Yankova-Tsvetkova E., Yurukova-Grancharova P., Aneva I., Zhelev P. (2021). On the reproductive potential in *Primula veris* L. (*Primulaceae*): Embryological features, pollen and seed viability, genetic diversity. Plants.

[49] Yankova-Tsvetkova E., Petrova M., Grigorova I., Traykova B., Stanilova M. (2022). The establishment of an ex situ collection of *Primula veris* in Bulgaria. Plants.

[50] Nikolova M., Yankova-Tsvetkova E., Stefanova T., Berkov S. (2023). Exudate flavonoids of *Primula veris* leaves and their inhibitory activity on *Lolium perrene* seed germination. Proc. Bulg. Acad. Sci..

[51] Budzianowski J., Morozowska M., Wesołowska M. (2005). Lipophilic Flavones of *Primula veris* L. from field cultivation and in vitro cultures. Phytochemistry.

[52] Matsumoto M. (1994). 2′-Hydroxy-4′-Methoxyacetophenone (Paeonol) in *Exacum affine* Cv. Biosci. Biotechnol. Biochem..

[53] Has M., Kucuk S., Kurkcuoglu M. (2021). Anatomical and Volatile Components Investigations on *Primula vulgaris* Huds. subsp. *vulgaris* (*Primulaceae*). Ann. Phytomedicine Int. J..

[54] Qu G.L., Zhang H.M., Deng Z.W., Kong D.Y., Geng Z.F., Du S.S. (2011). Study on chemical constituents of *Primula maximowiczii* Regel: Part III. Chin. Pharm. J..

[55] Xu S.J., Yang L., Zeng X., Zhang M., Wang Z.T. (2006). Characterization of compounds in the Chinese herbal drug mu-dan-pi by liquid chromatography coupled to electrospray ionization mass spectrometry. Rapid Commun. Mass Spectrom..

[56] Baczek K., Przybył J.L., Mirgos M., Kosakowska O., Szymborska-Sandhu I., Wȩglarz Z. (2017). Phenolics in *Primula veris* L. and *P. elatior* (L.) Hill raw materials. Int. J. Anal. Chem..

[57] Foubert K., Theunis M., Apers S., Vlietinck A.J., Pieters L. (2008). Chemistry, distribution and biological activities of 13,28-epoxy-oleanane saponins from the plant families *Myrsinaceae* and *Primulaceae*. Curr. Org. Chem..

[58] Damyanova T., Dimitrova P.D., Borisova D., Topouzova-Hristova T., Haladjova E., Paunova-Krasteva T. (2024). An overview of biofilm-associated infections and the role of phytochemicals and nanomaterials in their control and prevention. Pharmaceutics.

[59] Sarker S.D., Nahar L., Kumarasamy Y. (2007). Microtitre plate-based antibacterial assay incorporating resazurin as an indicator of cell growth, and its application in the in vitro antibacterial screening of phytochemicals. Methods.

[60] Święciło A., Rybczyńska-Tkaczyk K. (2020). Resazurin method for evaluation of bioactive compounds from cranberry extracts using the metabolic activity of a ΔSOD1 mutant of saccharomyces cerevisiae yeast under severe osmotic stress. J. AOAC Int..

[61] Hussain A.I., Anwar F., Nigam P.S., Sarker S.D., Moore J.E., Rao J.R., Mazumdar A. (2011). Antibacterial activity of some *Lamiaceae* essential oils using resazurin as an indicator of cell growth. LWT Food Sci. Technol..

[62] Khan S., Shaheen H., Mehmood A., Nasar S., Khan T. (2022). Ethnobotanical and antibacterial study of *Primula* plants traditionally used in the indigenous communities of Western Himalaya, Pakistan. Saudi J. Biol. Sci..

[63] El-Sayed N.R., Samir R., Abdel-Hafez L.J.M., Ramadan M.A. (2020). Olive leaf extract modulates quorum sensing genes and biofilm formation in multi-drug resistant *Pseudomonas aeruginosa*. Antibiotics.

[64] Paunova-Krasteva T., Hemdan B.A., Dimitrova P.D., Damyanova T., El-Feky A.M., Elbatanony M.M., Stoitsova S., El-Liethy M.A., El-Taweel G.E., El Nahrawy A.M. (2023). Hybrid Chitosan/CaO-based nanocomposites doped with plant extracts from *Azadirachta indica* and *Melia azedarach*: Evaluation of antibacterial and antibiofilm activities. Bionanoscience.

[65] Ansari F.A., Husain F.M., Pichtel J., Meena R.P., Khan M.H., Khan A.S., Alam N. (2025). *Withania somnifera* (L.) Dunal: A medicinally important plant inhibits pathogenic biofilms. Microbe.

[66] Laskoski L.V., Bandeira D.M., Batista J.M., da Costa W.F., Baeza L.C., Kuo L.H., Pinto F.G.d.S. (2022). Phytochemical prospection and evaluation of antimicrobial, antioxidant and antibiofilm activities of extracts and essential oil from leaves of *Myrsine umbellata* Mart. (*Primulaceae*). Braz. J. Biol..

[67] Ozay C., Temel A., Turel S., Akgul I. (2023). Investigation of anti-inflammatory, antibiofilm, antioxidant and cytotoxic activities of *Cyclamen hederifolium* (*Primulaceae*). Farmacia.

[68] Hamdan H.F., Ross E.E.R., Jalil M.T.M., Hashim M.A., Yahya M.F.Z.R. (2024). Antibiofilm efficacy and mode of action of *Etlingera elatior* extracts against *Staphylococcus aureus*. Malays. Appl. Biol..

[69] Dimitrova P.D., Ivanova V., Trendafilova A., Paunova-Krasteva T. (2024). Anti-biofilm and anti-quorum-sensing activity of *Inula* extracts: A strategy for modulating *Chromobacterium violaceum* virulence factors. Pharmaceuticals.

[70] Dimitrova P.D., Damyanova T., Paunova-Krasteva T. (2023). *Chromobacterium violaceum*: A model for evaluating the anti-quorum sensing activities of plant substances. Sci. Pharm..

[71] Yu Y., Kang X., Liu T., Wang Y., Tang J., Peng W., Martin F.M., Tan H. (2024). Inoculation of the *Morchella importuna* mycosphere with *Pseudomonas chlororaphis* alleviated a soil-borne disease caused by *Paecilomyces penicillatus*. Biol. Fertil. Soils..

[72] Matilla-Cuenca L., Gil C., Cuesta S., Rapún-Araiz B., Žiemytė M., Mira A., Lasa I., Valle J. (2020). Antibiofilm activity of flavonoids on *Staphylococcal* biofilms through targeting BAP amyloids. Sci. Rep..

[73] Zeng Y., Nikitkova A., Abdelsalam H., Li J., Xiao J. (2019). Activity of quercetin and kaemferol against *Streptococcus mutans* biofilm. Arch. Oral. Biol..

[74] Pruteanu M., Hernández Lobato J.I., Stach T., Hengge R. (2020). Common plant flavonoids prevent the assembly of amyloid curli fibres and can interfere with bacterial biofilm formation. Environ. Microbiol..

[75] Parai D., Islam E., Mitra J., Mukherjee S.K. (2016). Effect of *Bacoside* a on growth and biofilm formation by *Staphylococcus aureus* and *Pseudomonas aeruginosa*. Can. J. Microbiol..

[76] Stanković J., Godevac D., Tešević V., Dajić-Stevanović Z., Ćirić A., Soković M., Novaković M. (2019). Antibacterial and antibiofilm activity of flavonoid and saponin derivatives from *Atriplex tatarica* against *Pseudomonas aeruginosa*. J. Nat. Prod..

[77] Chen Y., Gao Y., Li Y., Yin J. (2024). Anti-biofilm activity of assamsaponin A, theasaponin E1, and theasaponin E2 against *Candida albicans*. Int. J. Mol. Sci..

[78] Araújo N.J.S., Silva A.R.P., Costa M.S., Freitas T.S., Filho J.M.B., Matos Y.M.L.S., Morais-Braga M.F.B., Pereira Junior F.N., Silva C.A.P., Souza E.O. (2022). Chemical characterization UPLC-ESI-QToF-MSE, antibacterial and antibiofilm potential of *Sarcomphalus joazeiro* (MART.) Hauenschild. Food Biosci..

[79] Choudhary M., Verma V., Saran R., Bhagyawant S.S., Srivastava N. (2022). Natural biosurfactant as antimicrobial agent: Strategy to action against fungal and bacterial activities. Cell. Biochem. Biophys..

[80] Yadav H., Mahalvar A., Pradhan M., Yadav K., Kumar Sahu K., Yadav R. (2023). Exploring the potential of phytochemicals and nanomaterial: A boon to antimicrobial treatment. Med. Drug Discov..

[81] Silva E., Teixeira J.A., Pereira M.O., Rocha C.M.R., Sousa A.M. (2023). Evolving biofilm inhibition and eradication in clinical settings through plant-based antibiofilm agents. Phytomedicine.

[82] Wang P., Henning S.M., Heber D. (2010). Limitations of MTT and MTS-based assays for measurement of antiproliferative activity of green tea polyphenols. PLoS ONE.

[83] Koczurkiewicz P., Czyz J., Podolak I., Wójcik K., Galanty A., Janeczko Z., Michalik M. (2015). Multidirectional effects of triterpene saponins on cancer cells—mini-review of in vitro studies. Acta Biochim. Pol.

[84] Francis G., Kerem Z., Makkar H.P.S., Becker K. (2002). The biological action of saponins in animal systems: A review. Br. J. Nutr..

[85] Sun X.H., Chai Y.H., Bai X.T., Li H.X., Yang P.P., Xi Y.M. (2025). Saikosaponin A mediates the anti-acute myeloid leukemia effect via the P-JNK signaling pathway induced by endoplasmic reticulum stress. Drug Des. Devel. Ther..

[86] De Soyza A., Hall A.J., Mahenthiralingam E., Drevinek P., Kaca W., Drulis-Kawa Z., Stoitsova S.R., Toth V., Coenye T., Zlosnik J.E.A. (2013). Developing an international *Pseudomonas aeruginosa* reference panel. Microbiologyopen.

[87] Trendafilova A., Staleva P., Petkova Z., Ivanova V., Evstatieva Y., Nikolova D., Rasheva I., Atanasov N., Topouzova-Hristova T., Veleva R. (2023). Phytochemical profile, antioxidant potential, antimicrobial activity, and cytotoxicity of dry extract from *Rosa damascena* Mill. Molecules.

