# Supplementary material for: Phytochemical Characterization and Anti-Biofilm Activity of Primula veris L. Roots
Source: Molecules. 2025 Apr 10;30(8):1702. doi: 10.3390/molecules30081702 (PMC12029431; doi:10.3390/molecules30081702)
Supplement: Supplementary file 1 [file molecules-30-01702-s001.zip › molecules-3525829-supplementary/Supplementary_I.pdf]

Phytochemical Characterization and Anti-Biofilm Activity of *Primula veris* L. Roots

Antoaneta Trendafilova [1,*], Desislava Raykova [1], Viktoria Ivanova [1], Miroslav Novakovic [2], Paraskev Nedialkov [3], Tsvetelina Paunova-Krasteva [4], Ralitsa Veleva [5] and Tanya Topouzova-Hristova [5]

1 Institute of Organic Chemistry with Centre of Phytochemistry, Bulgarian Academy of Sciences, 1113 Sofia, Bulgaria; desislava.raykova@orgchm.bas.bg (D.R.); viktoria.genova@orgchm.bas.bg (V.I.)
2 Institute of Chemistry, Technology and Metallurgy, National Institute of the Republic of Serbia, University of Belgrade, 11000 Belgrade, Serbia; mironov@chem.bg.ac.rs
3 Pharmacognosy Department, Faculty of Pharmacy, Medical University of Sofia, 1000 Sofia, Bulgaria; pnedialkov@pharmfac.mu-sofia.bg
4 Stephan Angeloff Institute of Microbiology, Bulgarian Academy of Sciences, 1113 Sofia, Bulgaria; pauny@abv.bg
5 Department of Cellular and Developmental Biology, Faculty of Biology, Sofia University St Kliment Ohridski, 1164 Sofia, Bulgaria; ralitsa_veleva@biofac.uni-sofia.bg (R.V.); topouzova@biofac.uni-sofia.bg (T.T.-H.)
* Correspondence: antoaneta.trendafilova@orgchm.bas.bg

SUPPLEMENTARY PART I

^{1}H NMR data of the known compounds 1-17

Content:

Figure SI-1. ^{1}H NMR of flavone (**1**) in $CDCl_3$

Figure SI-2. ^{1}H NMR of 2'-methoxyflavone (**2**) in $CDCl_3$

Figure SI-3. ^{1}H NMR of 3'-methoxyflavone (**3**) in $CDCl_3$

Figure SI-4. ^{1}H NMR of a mixture of 2',5'-dimethoxyflavone (**4**) and 3'-methoxy-4',5'-methylendioxyflavone (**6**) in $CDCl_3$

Figure SI-5. ^{1}H NMR of 3'-hydroxy- 4',5'-dimethoxyflavone (**5**) in $CDCl_3$

Figure SI-6. ^{1}H NMR of a mixture of 4-hydroxyacetophenone (**7**) and 4-hydroxy-3-methoxyacetophenone (**8**) in CD_3OD

Figure SI-7. ^{1}H NMR of 2-hydroxy-4-methoxyacetophenone (paeonol) (**9**) in $CDCl_3$

Figure SI-8. ^{1}H NMR of riccardin C (**10**) in CD_3OD

Figure SI-9. ^{1}H NMR of 8'-oxoriccardin C (**11**) in CD_3OD

Figure SI-10. ^{1}H NMR of 8'-hydroxyisomarchantin C (**12**) in CD_3OD

Figure SI-11. ^{1}H NMR of 8'-hydroxydihydroptyhantol A (**13**) in $CDCl_3$

Figure SI-12. ^{1}H NMR of a mixture of primulaverin (**14**) and primeverin (**15**) in CD_3OD

Figure SI-13. ^{1}H NMR of a mixture of 2-primeverosyl-5-methoxy-acetophenone (**16**) and paeonolide (**17**) in CD_3OD

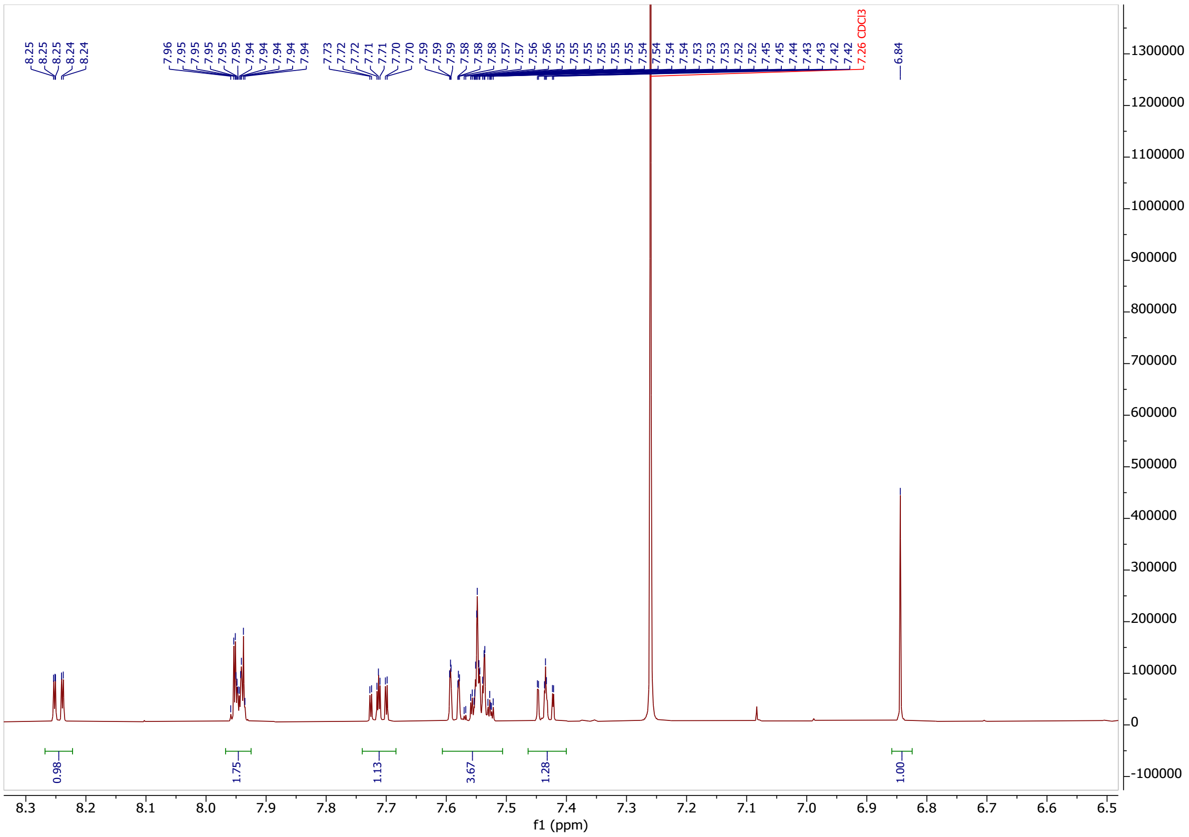

Figure SI-1. ^{1}H NMR of flavone (**1**) in $CDCl_3$

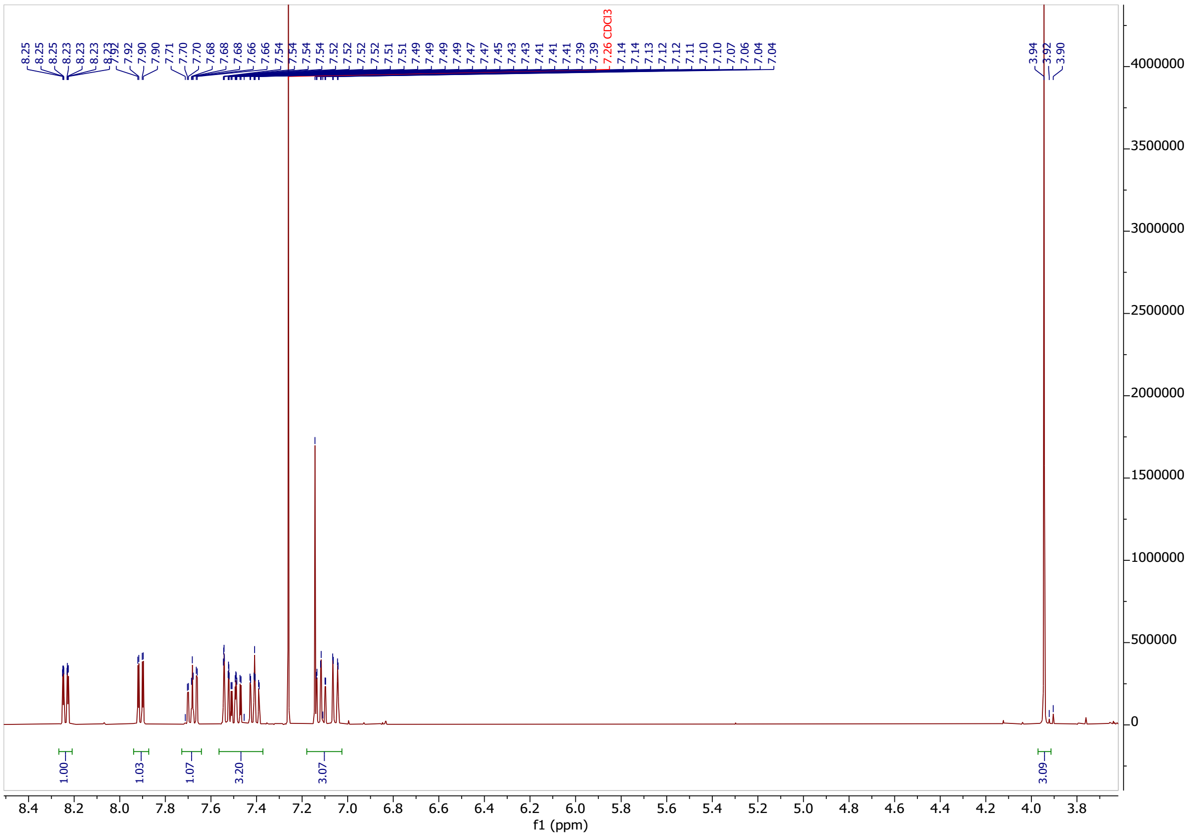

Figure SI-2. ^{1}H NMR of 2'-methoxyflavone (**2**) in $CDCl_3$

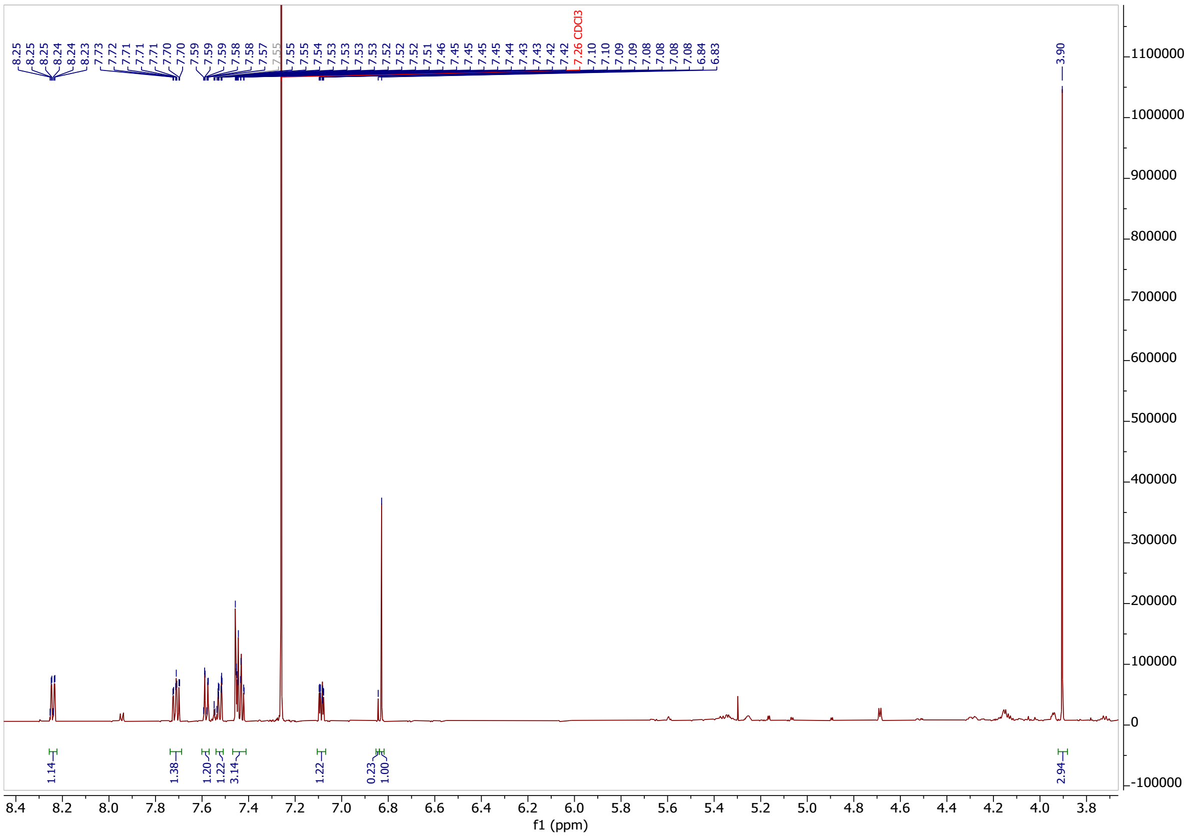

Figure SI-3. ^{1}H NMR of 3'-methoxyflavone (**3**) in $CDCl_3$

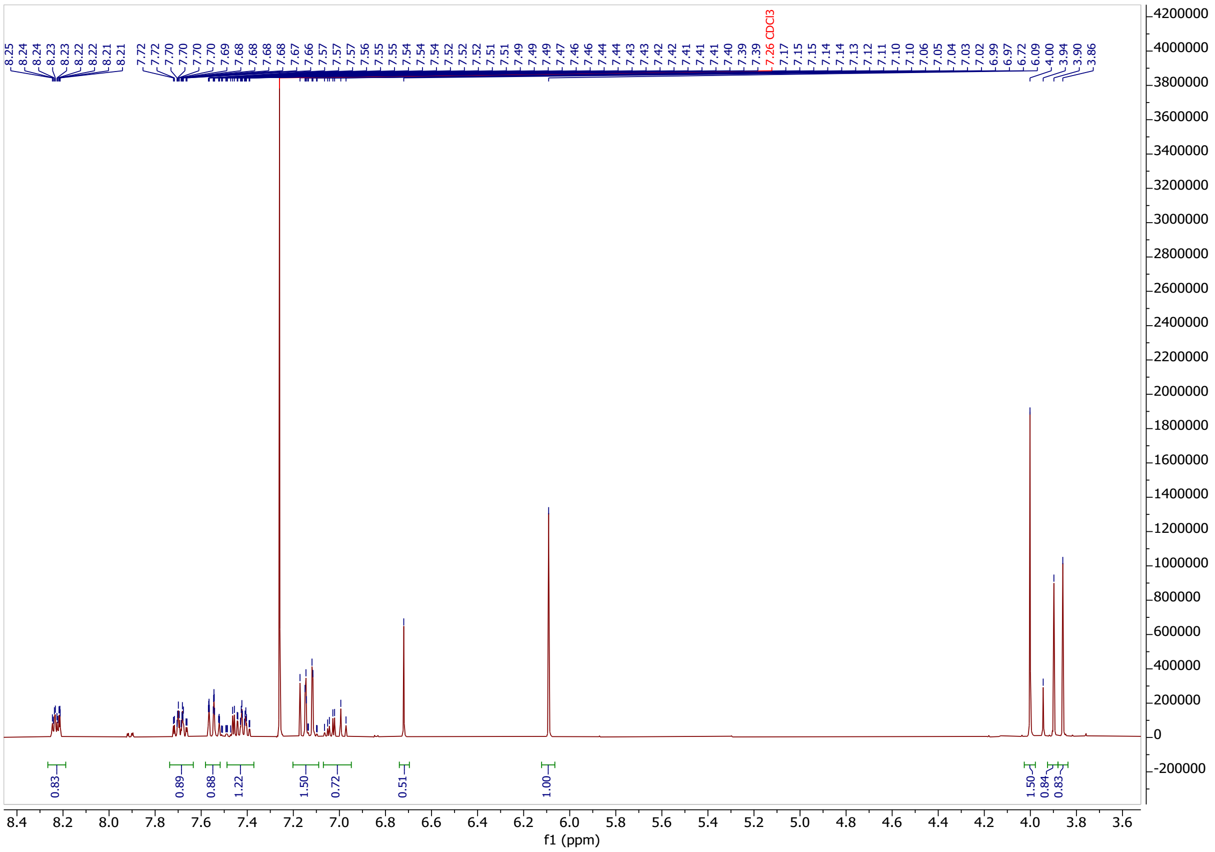

Figure SI-4. ^{1}H NMR of a mixture of 2',5'-dimethoxyflavone (**4**) and 3'-methoxy-4',5'-methylendioxyflavone (**6**) in $CDCl_3$

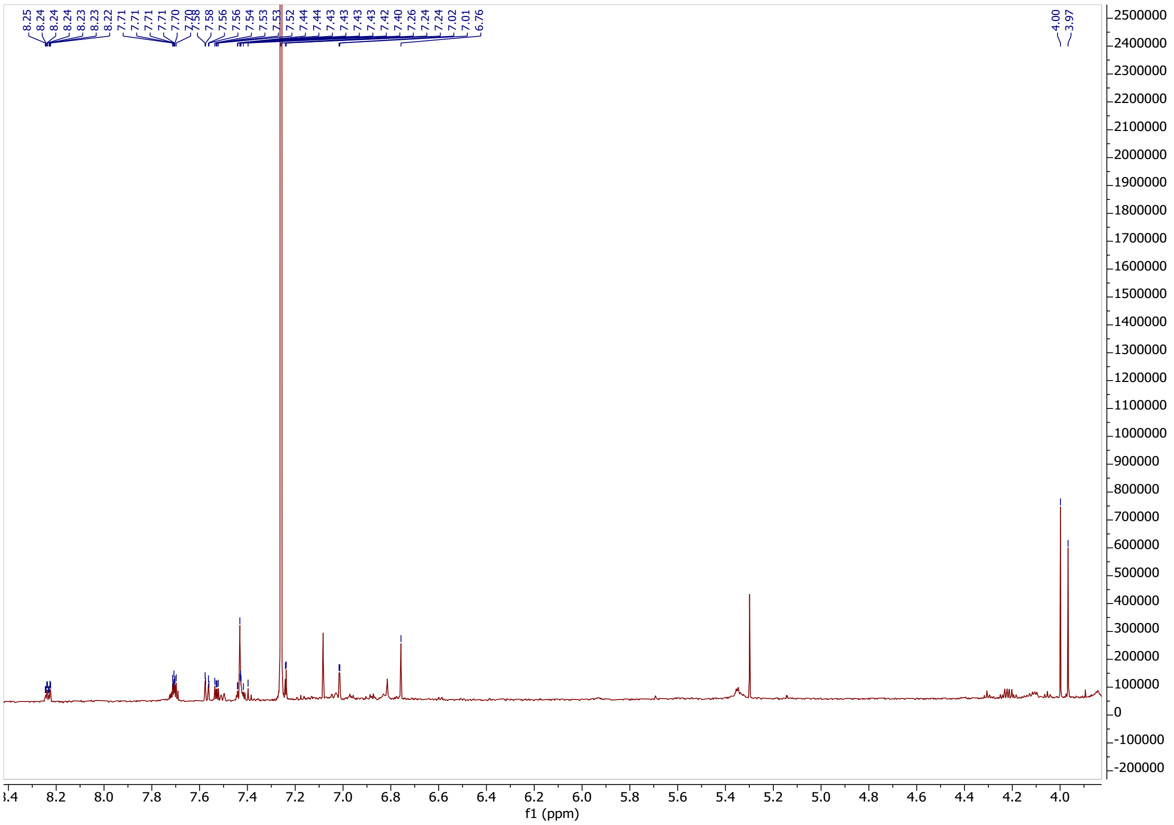

Figure SI-5. ^{1}H NMR of 3'-hydroxy- 4',5'-dimethoxyflavone (**5**) in $CDCl_3$

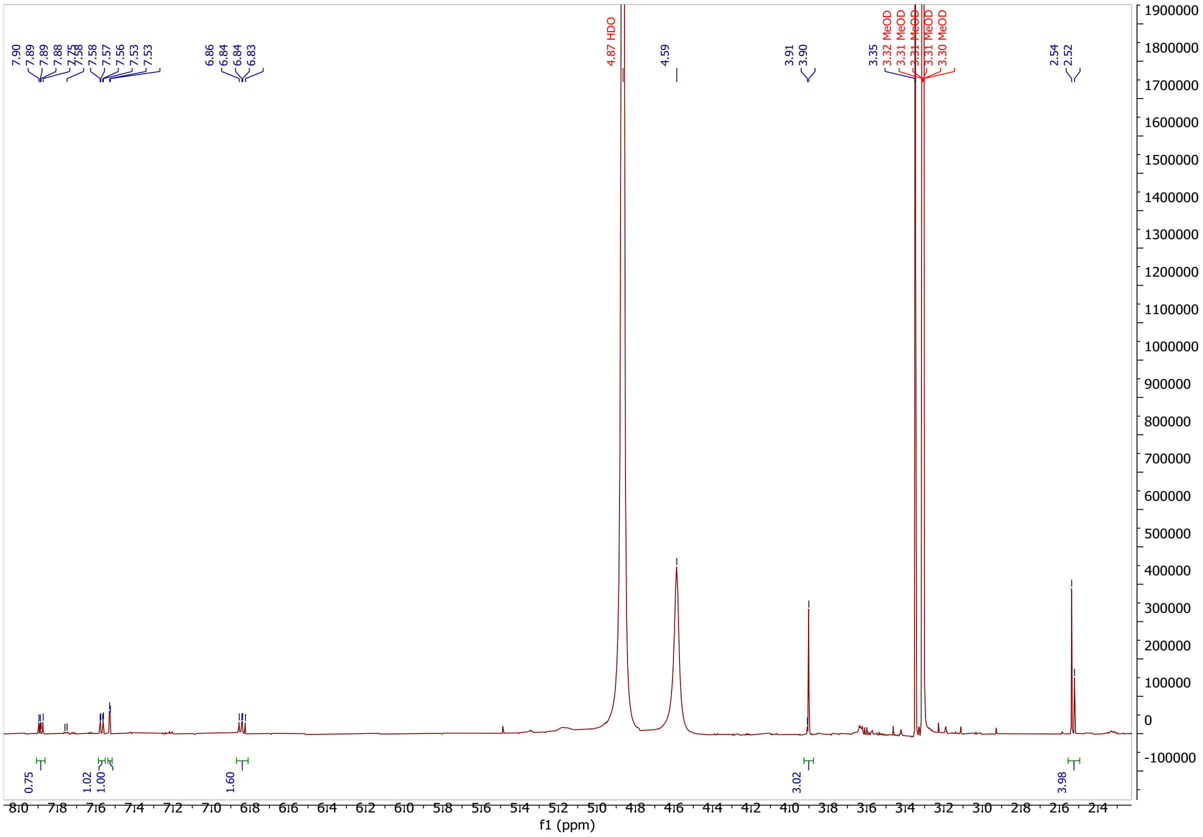

Figure SI-6. ^{1}H NMR of a mixture of 4-hydroxyacetophenone (**7**) and 4-hydroxy-3-methoxyacetophenone (**8**) in CD_3OD

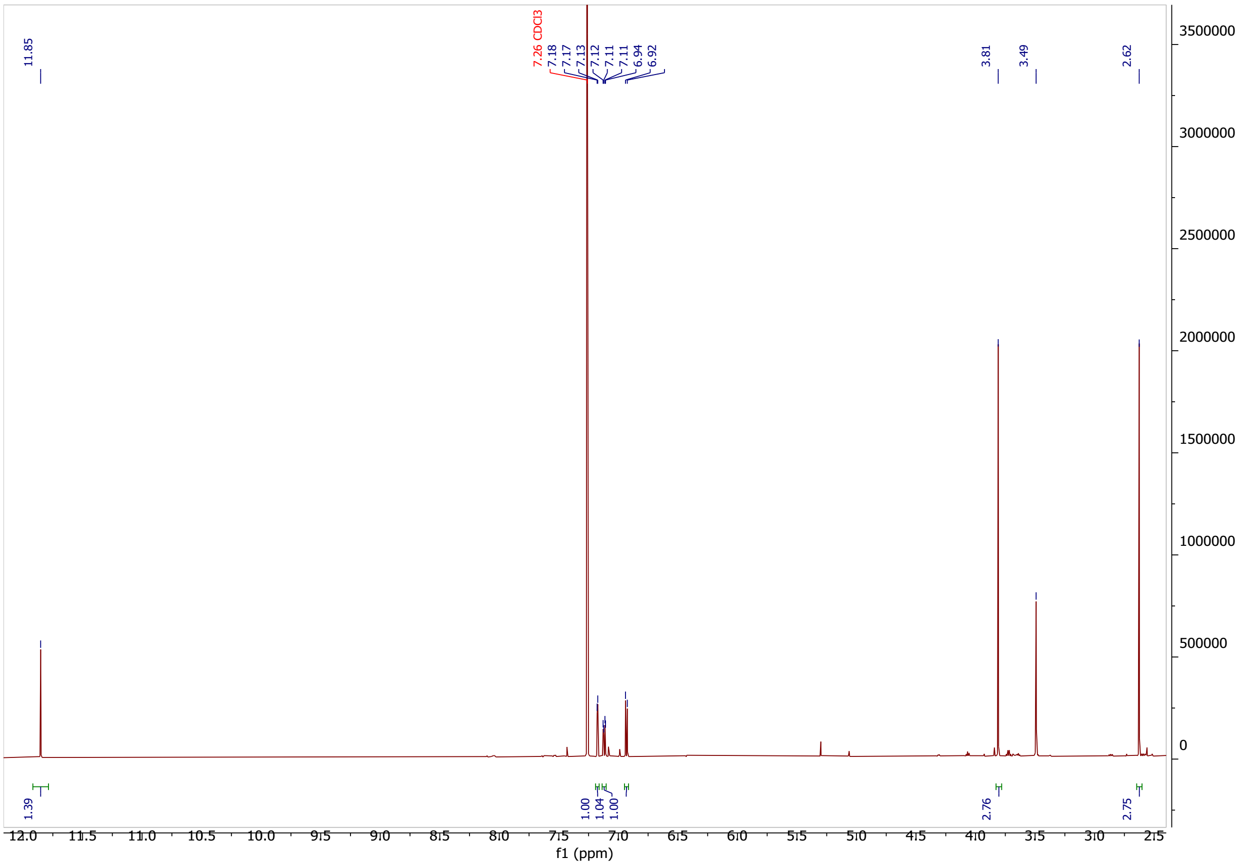

Figure SI-7. ^{1}H NMR of 2-hydroxy-4-methoxyacetophenone (paeonol) (**9**) in $CDCl_3$

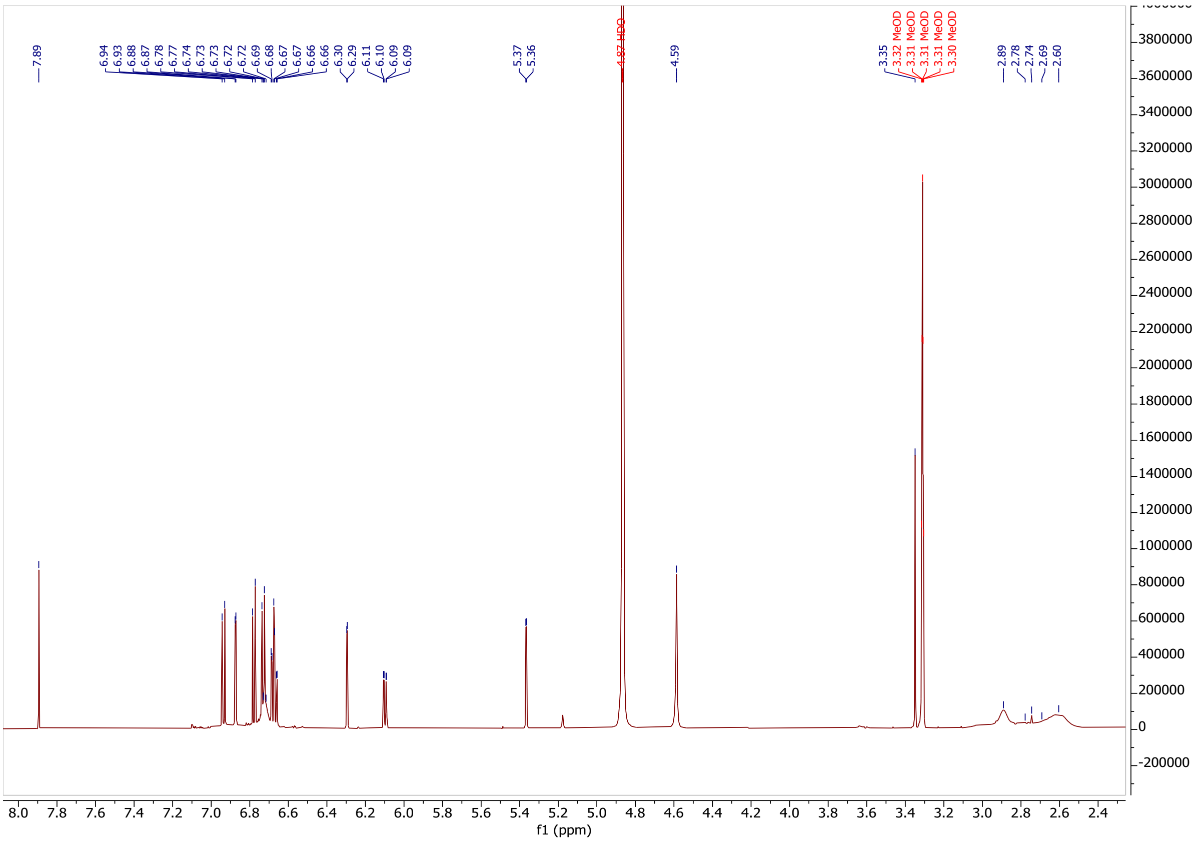

Figure SI-8. ^{1}H NMR of riccardin C (**10**) in CD_3OD

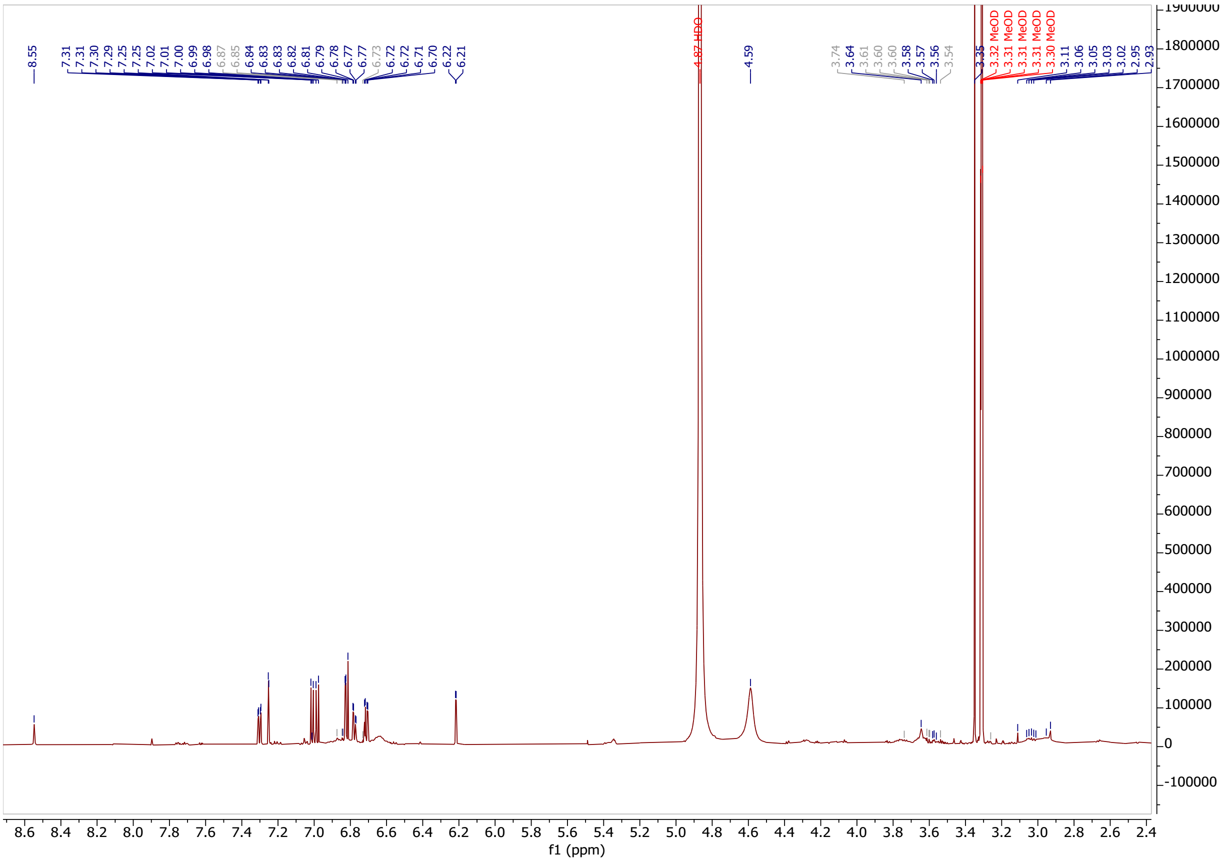

Figure SI-9. ^{1}H NMR of 8'-oxoriccardin C (**11**) in CD_3OD

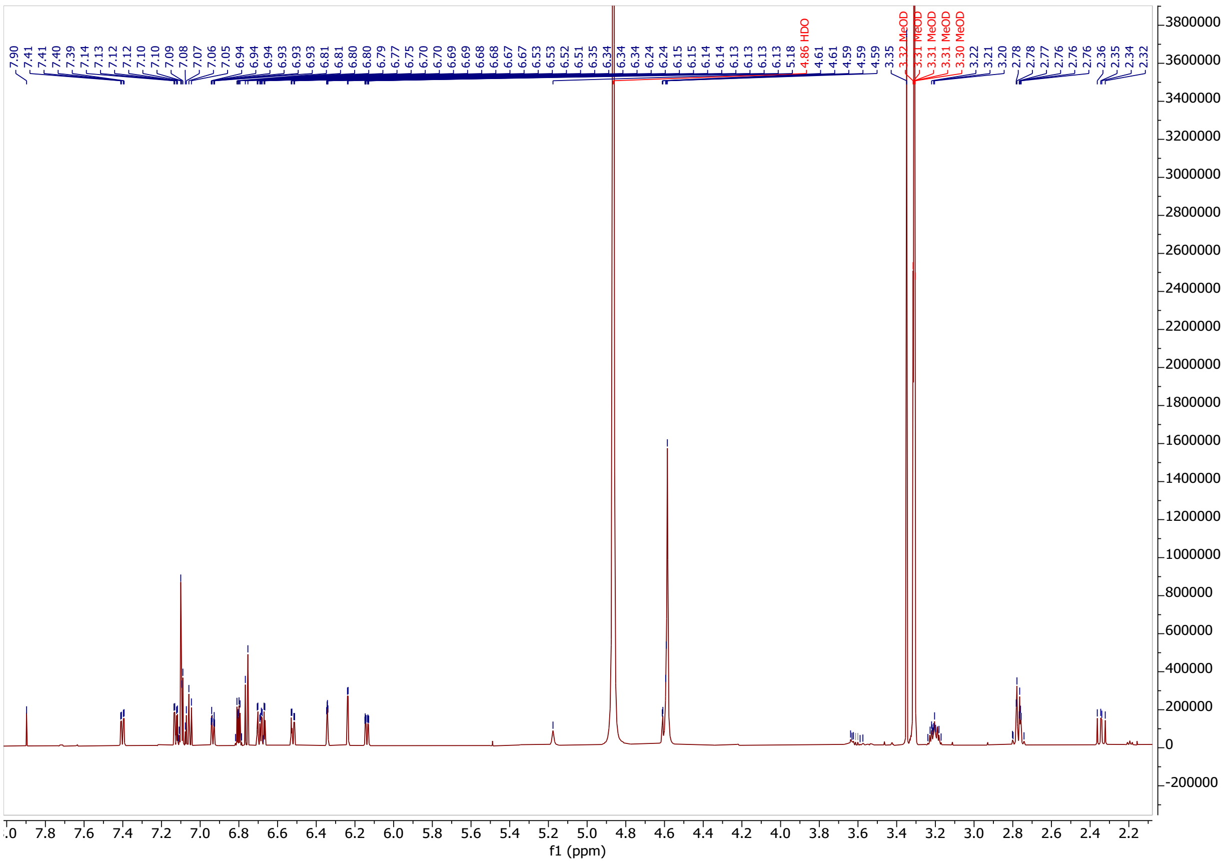

Figure SI-10. ^{1}H NMR of 8'-hydroxyisomarchantin C (**12**) in CD_3OD

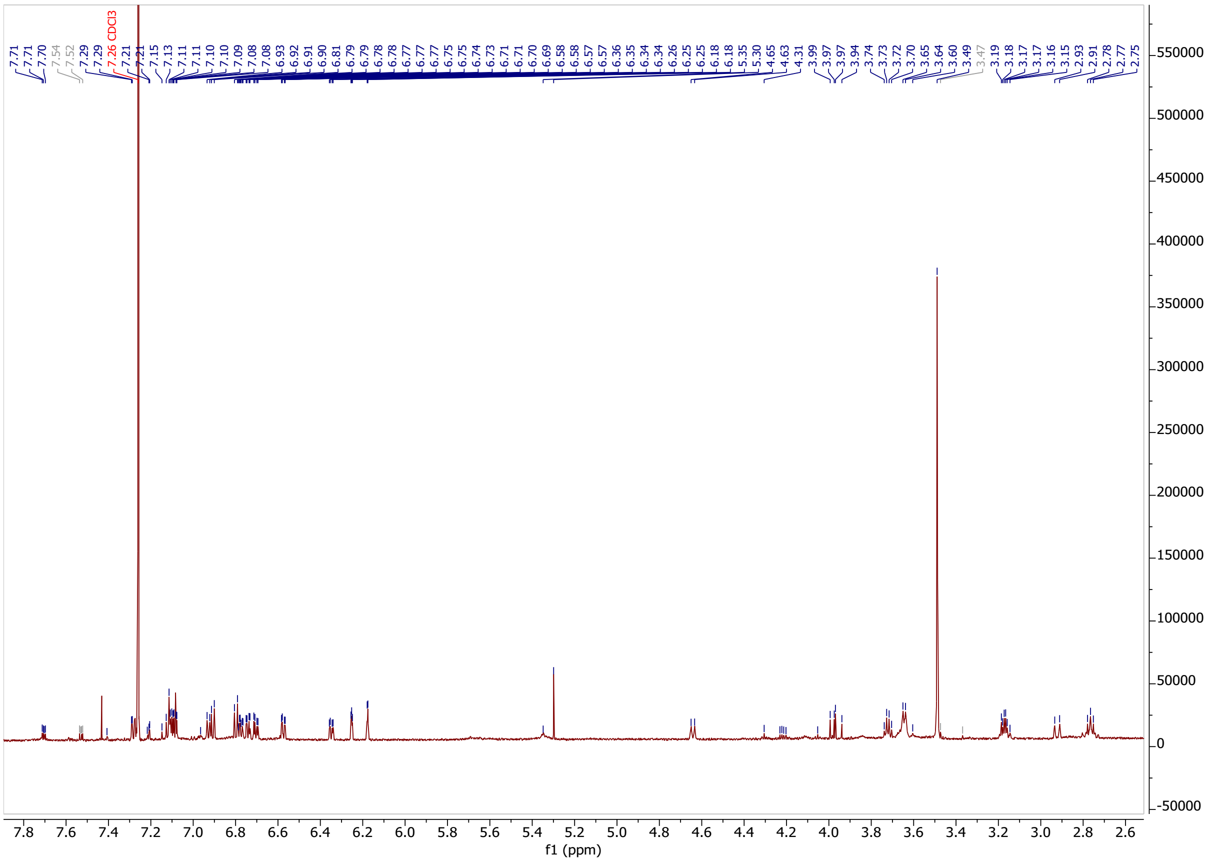

Figure SI-11. ^{1}H NMR of 8'-hydroxydihydroptyhantol A (**13**) in $CDCl_3$

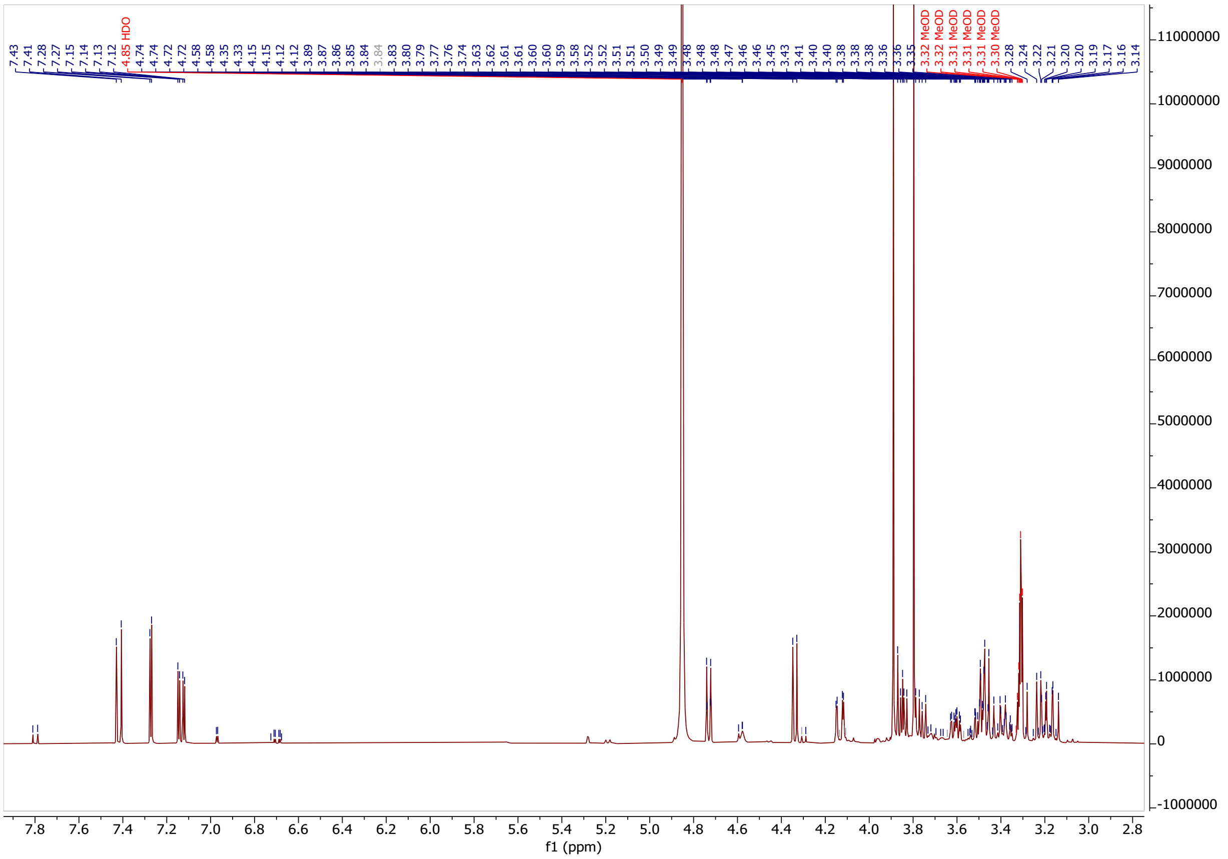

Figure SI-12. ^{1}H NMR of a mixture of primulaverin (**14**) and primeverin (**15**) in CD_3OD

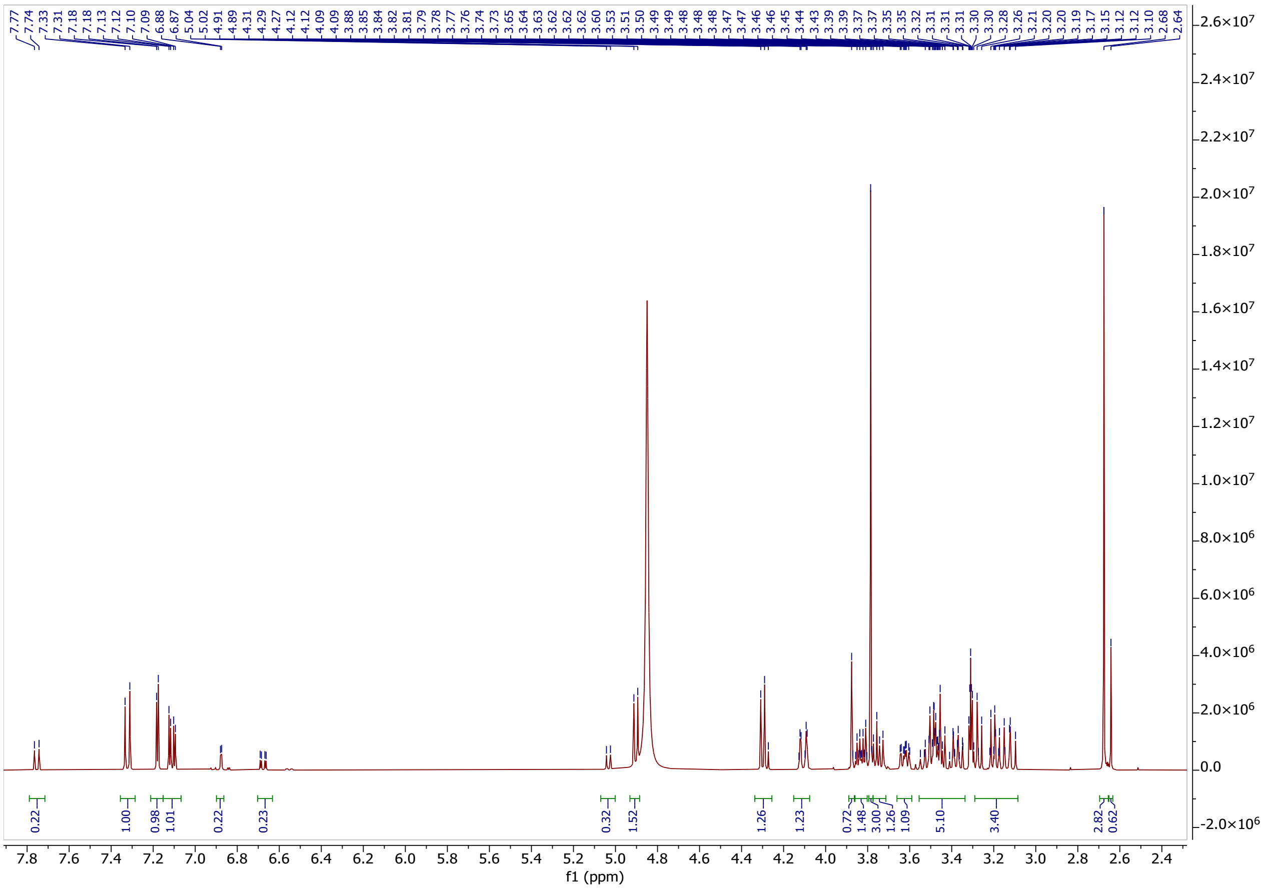

Figure SI-13. ^{1}H NMR of a mixture of 2-primeverosyl-5-methoxy-acetophenone (**16**) and paeonolide (**17**) in CD_3OD
