# Supplementary material for: Phytochemical Characterization and Anti-Biofilm Activity of Primula veris L. Roots
Source: Molecules. 2025 Apr 10;30(8):1702. doi: 10.3390/molecules30081702 (PMC12029431; doi:10.3390/molecules30081702)
Supplement: Supplementary file 1 [file molecules-30-01702-s001.zip › molecules-3525829-supplementary/Supplementary_II.pdf]

Phytochemical Characterization and Anti-Biofilm Activity of *Primula veris* L. Roots

Antoaneta Trendafilova [1,*], Desislava Raykova [1], Viktoria Ivanova [1], Miroslav Novakovic [2], Paraskev Nedialkov [3], Tsvetelina Paunova-Krasteva [4], Ralitsa Veleva [5] and Tanya Topouzova-Hristova [5]

1. Institute of Organic Chemistry with Centre of Phytochemistry, Bulgarian Academy of Sciences, 1113 Sofia, Bulgaria; desislava.raykova@orgchm.bas.bg (D.R.); viktoria.genova@orgchm.bas.bg (V.I.)
2. Institute of Chemistry, Technology and Metallurgy, National Institute of the Republic of Serbia, University of Belgrade, 11000 Belgrade, Serbia; mironov@chem.bg.ac.rs
3. Pharmacognosy Department, Faculty of Pharmacy, Medical University of Sofia, 1000 Sofia, Bulgaria; pnedialkov@pharmfac.mu-sofia.bg
4. Stephan Angeloff Institute of Microbiology, Bulgarian Academy of Sciences, 1113 Sofia, Bulgaria; pauny@abv.bg
5. Department of Cellular and Developmental Biology, Faculty of Biology, Sofia University St Kliment Ohridski, 1164 Sofia, Bulgaria; ralitsa_veleva@biofac.uni-sofia.bg (R.V.); topouzova@biofac.uni-sofia.bg (T.T.-H.)

* Correspondence: antoaneta.trendafilova@orgchm.bas.bg

SUPPLEMENTARY PART II

Spectral data of primulasaponin I (18) and new saponins 19-21

Content:

Figure SII-1. Stacked ^{1}H NMR spectra of compounds **18-21** in CD_3OD

Figure SII-2. ^{1}H NMR spectrum of primulasaponin I (**18**) in CD_3OD

Figure SII-3. ^{13}C NMR spectrum of primulasaponin I (**18**) in CD_3OD

Figure SII-4. ^{13}C DEPT-135 NMR spectrum of primulasaponin I (**18**) in CD_3OD

Figure SII-5. COSY spectrum of primulasaponin I (**18**) in CD_3OD

Figure SII-6. HSQC spectrum of primulasaponin I (**18**) in CD_3OD

Figure SII-7. HMBC spectrum of primulasaponin I (**18**) in CD_3OD

Figure SII-8. ROESY spectrum of primulasaponin I (**18**) in CD_3OD

Figure SII-9. HRMS spectrum of primulasaponin I (**18**) in negative ionization mode

Figure SII-10. ^{1}H NMR spectrum of primulasaponin III (**19**) in CD_3OD

Figure SII-11. ^{13}C NMR spectrum of primulasaponin III (**19**) in CD_3OD

Figure SII-12. ^{13}C DEPT-135 NMR spectrum of primulasaponin III (**19**) in CD_3OD

Figure SII-13. COSY spectrum of primulasaponin III (**19**) in CD_3OD

Figure SII-14. HSQC spectrum of primulasaponin III (**19**) in CD_3OD

Figure SII-15. HMBC spectrum of primulasaponin III (**19**) in CD_3OD

Figure SII-16. ROESY spectrum of primulasaponin III (**19**) in CD_3OD

Figure SII-17. HRMS spectrum of primulasaponin III (**19**) in negative ionization mode

Figure SII-18. IR (ATR) spectrum of primulasaponin III (**19**)

Figure SII-19. ^1H NMR spectrum of primulasaponin IV (**20**) in CD_3OD

Figure SII-20. ^{13}C NMR spectrum of primulasaponin IV (**20**) in CD_3OD

Figure SII-21. ^{13}C DEPT-135 NMR spectrum of primulasaponin IV (**20**) in CD_3OD

Figure SII-22. COSY spectrum of primulasaponin IV (**20**) in CD_3OD

Figure SII-23. HSQC spectrum of primulasaponin IV (**20**) in CD_3OD

Figure SII-24. HMBC spectrum of primulasaponin IV (**20**) in CD_3OD

Figure SII-25. ROESY spectrum of primulasaponin IV (**20**) in CD_3OD

Figure SII-26. HRMS spectrum of primulasaponin IV (**20**) in negative ionization mode

Figure SII-27. IR (ATR) spectrum of primulasaponin IV (**20**)

Figure SII-28. ^1H NMR spectrum of primulasaponin V (**21**) in CD_3OD

Figure SII-29. ^{13}C NMR spectrum of primulasaponin V (**21**) in CD_3OD

Figure SII-30. ^{13}C DEPT-135 NMR spectrum of primulasaponin V (**21**) in CD_3OD

Figure SII-31. COSY spectrum of primulasaponin V (**21**) in CD_3OD

Figure SII-32. HSQC spectrum of primulasaponin V (**21**) in CD_3OD

Figure SII-33. HMBC spectrum of primulasaponin V (**21**) in CD_3OD

Figure SII-34. ROESY spectrum of primulasaponin V (**21**) in CD_3OD

Figure SII-35. HRMS spectrum of primulasaponin V (**21**) in negative ionization mode

Figure SII-36. IR (ATR) spectrum of primulasaponin V (**21**)

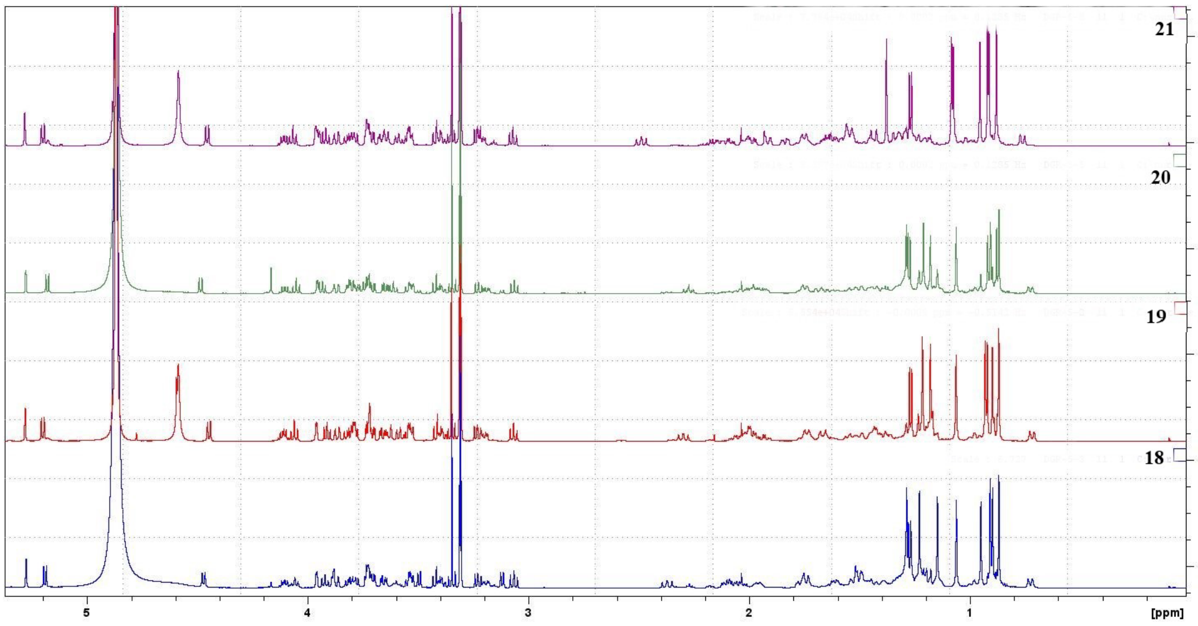

Figure SII-1. Stacked ^{1}H NMR spectra of compounds **18-21** in CD_3OD

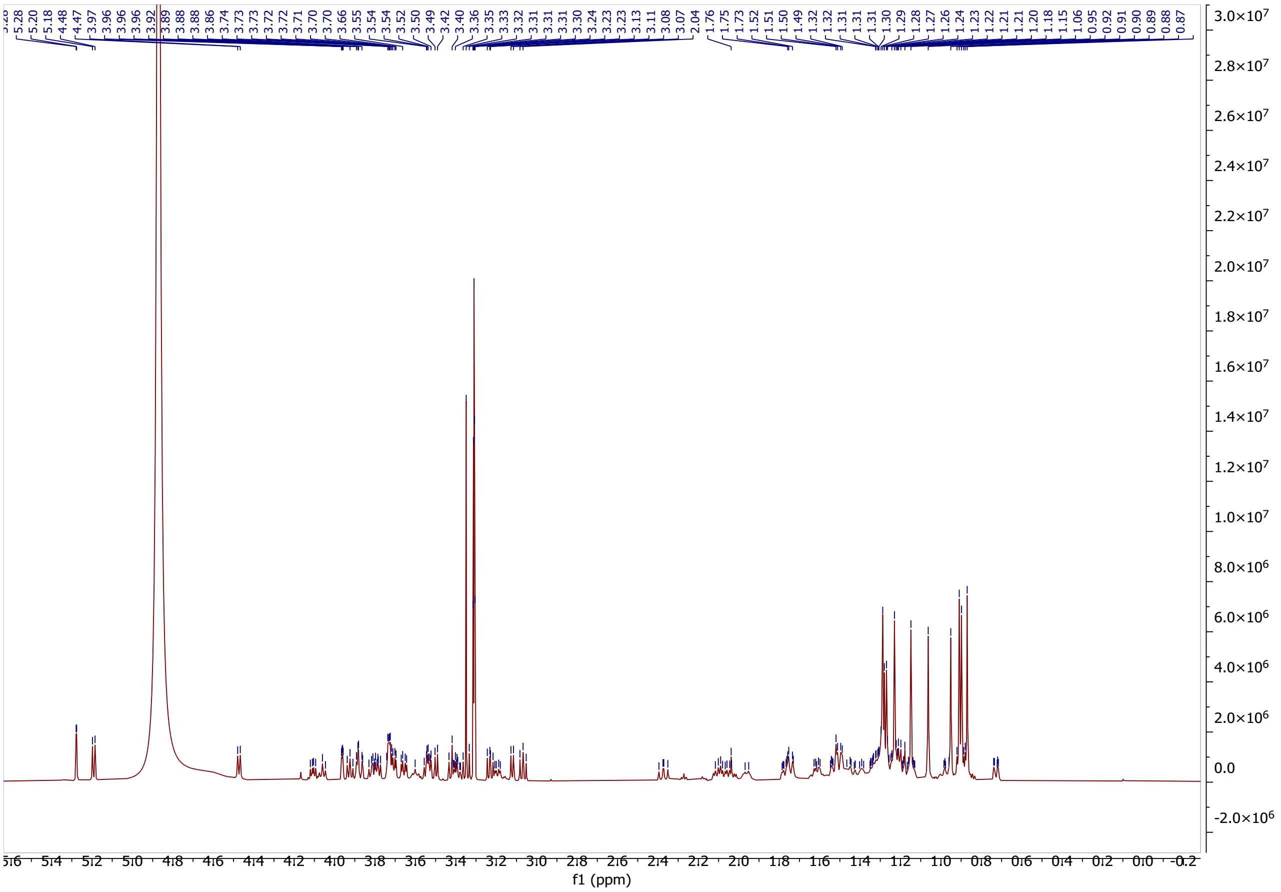

Figure SII-2. ^{1}H NMR spectrum of primulasaponin I (**18**) in CD_3OD

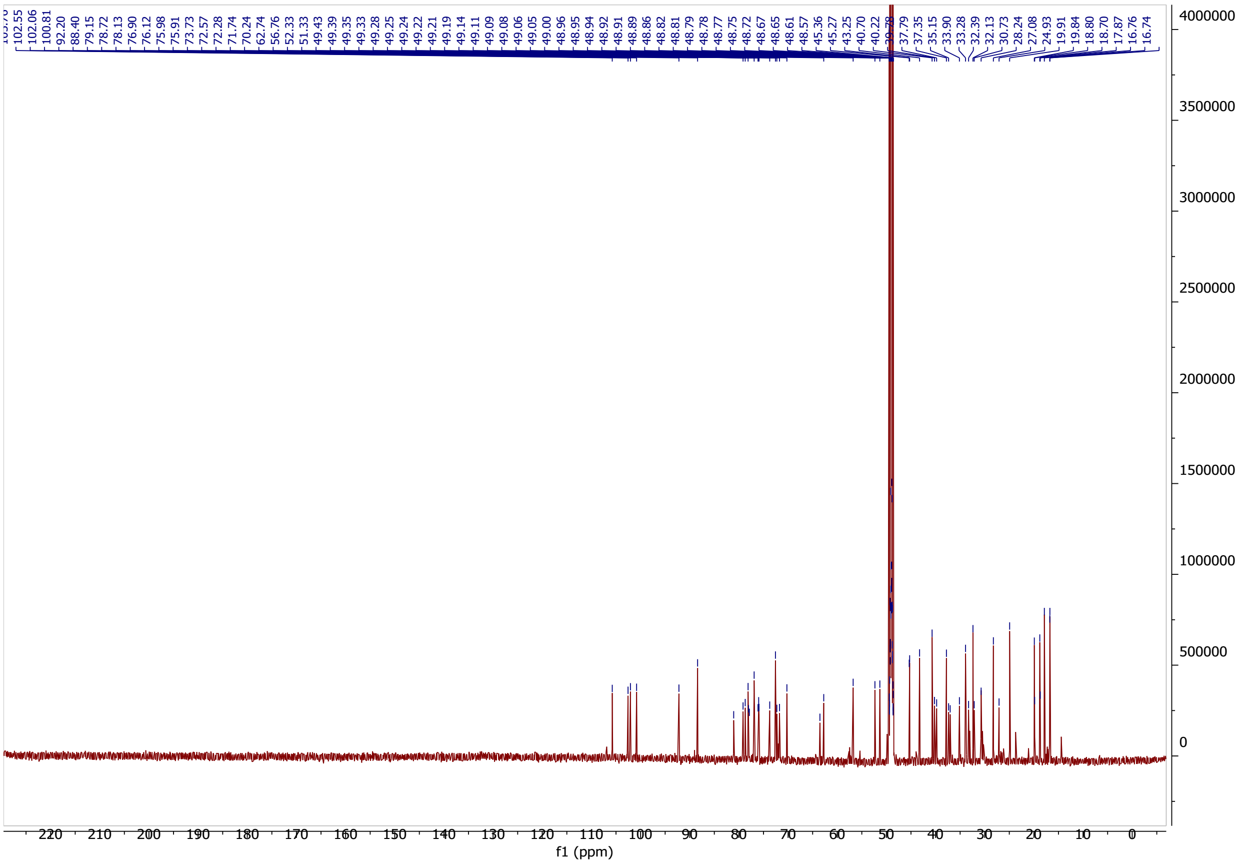

Figure SII-3. ^{13}C NMR spectrum of primulasaponin I (**18**) in CD_3OD

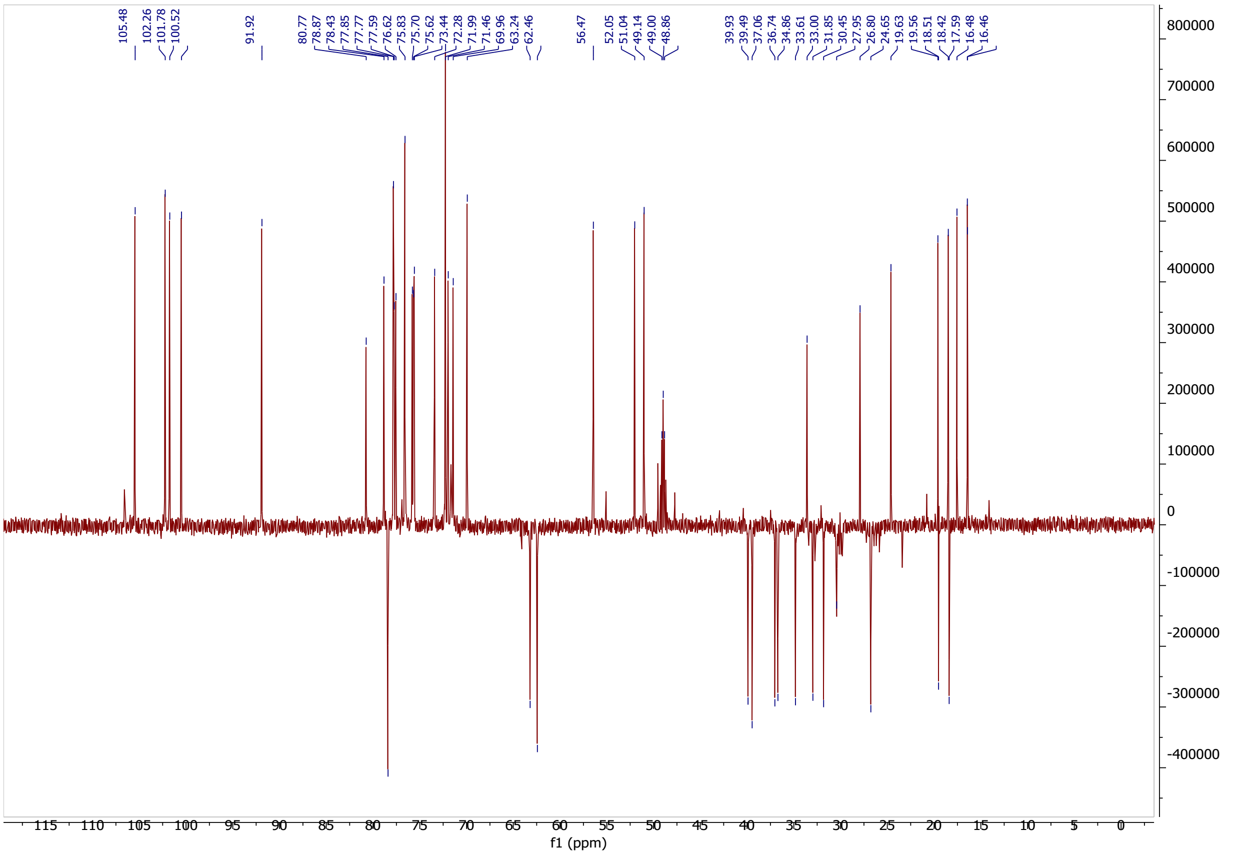

Figure SII-4. ^{13}C DEPT-135 NMR spectrum of primulasaponin I (**18**) in CD_3OD

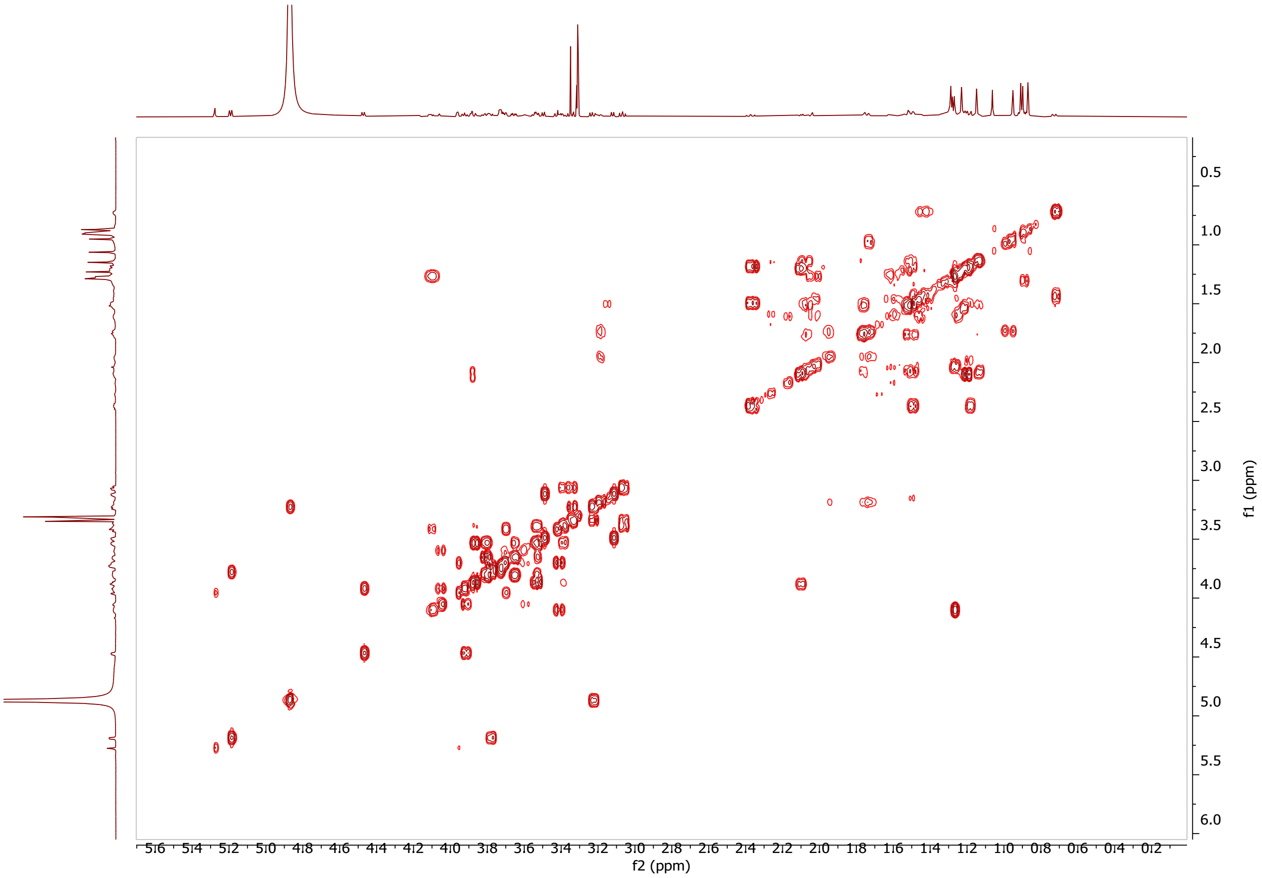

Figure SII-5. COSY spectrum of primulasaponin I (**18**) in CD_3OD

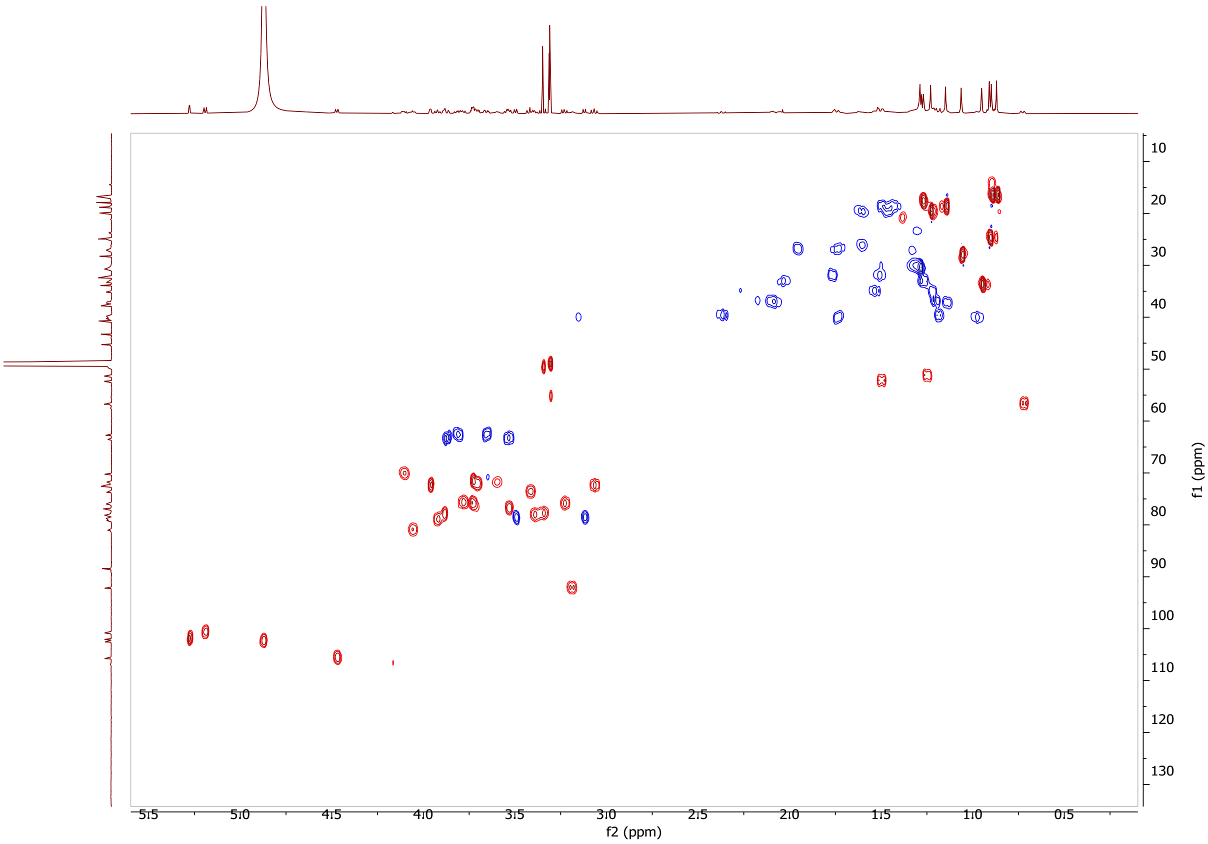

Figure SII-6. HSQC spectrum of primulasaponin I (**18**) in CD_3OD

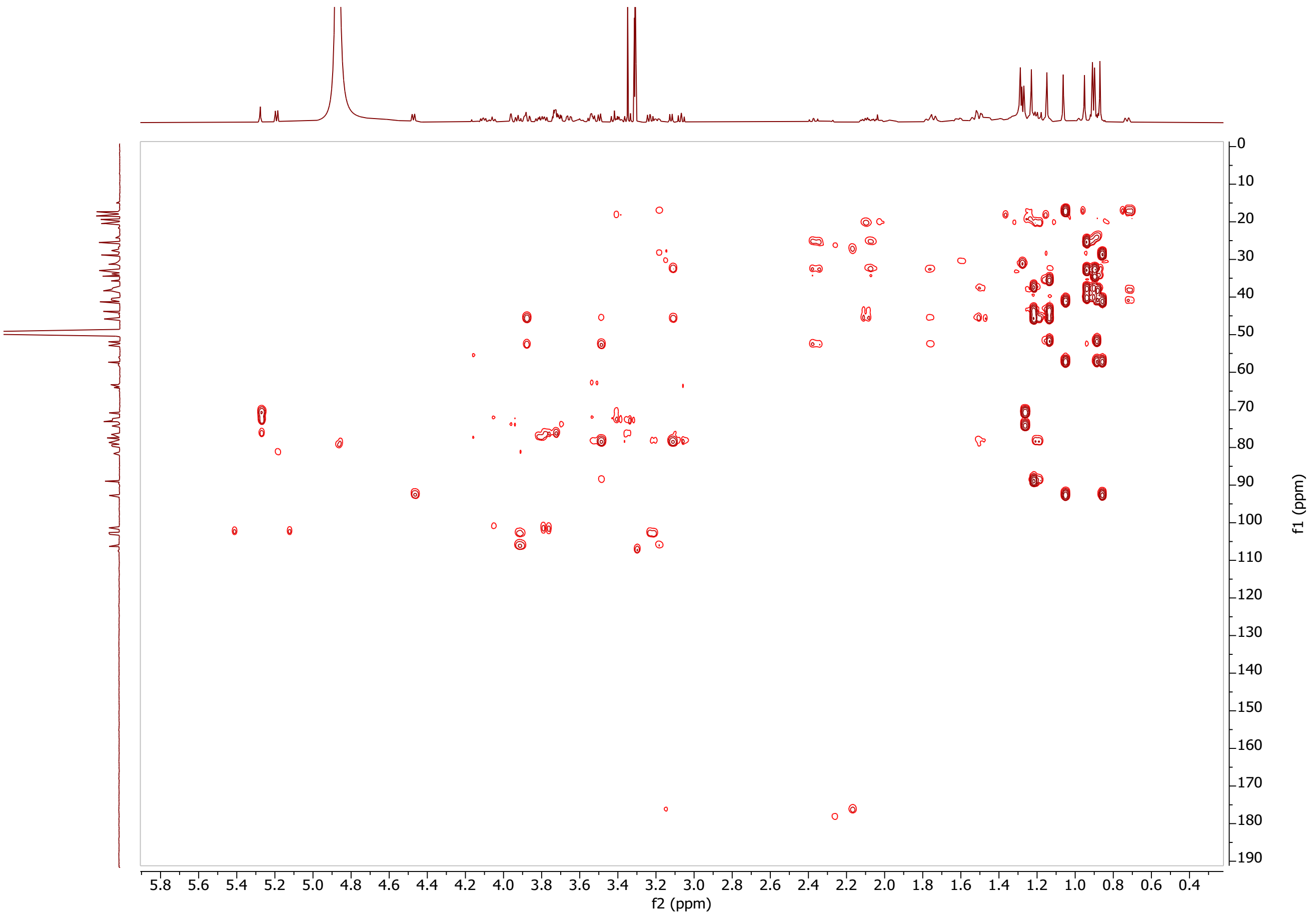

Figure SII-7. HMBC spectrum of primulasaponin I (**18**) in CD_3OD

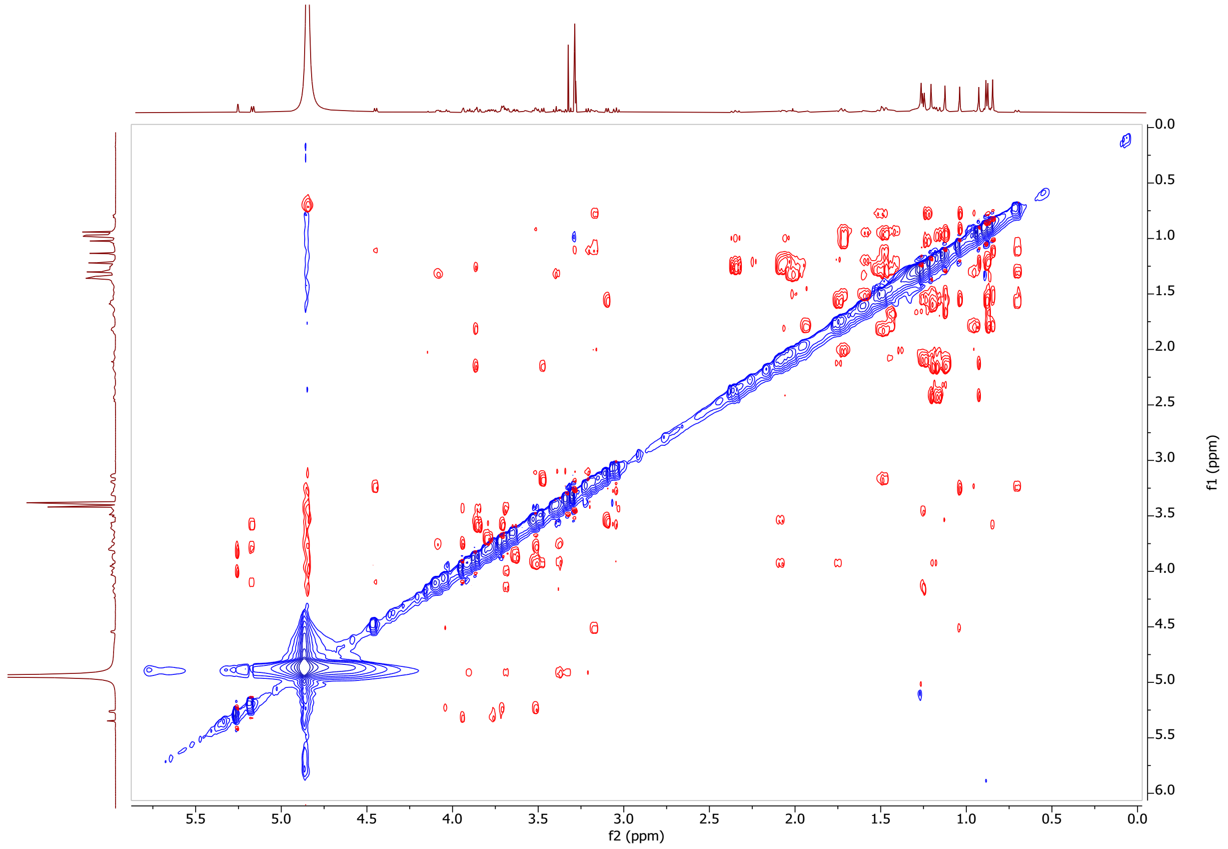

Figure SII-8. ROESY spectrum of primulasaponin I (**18**) in CD_3OD

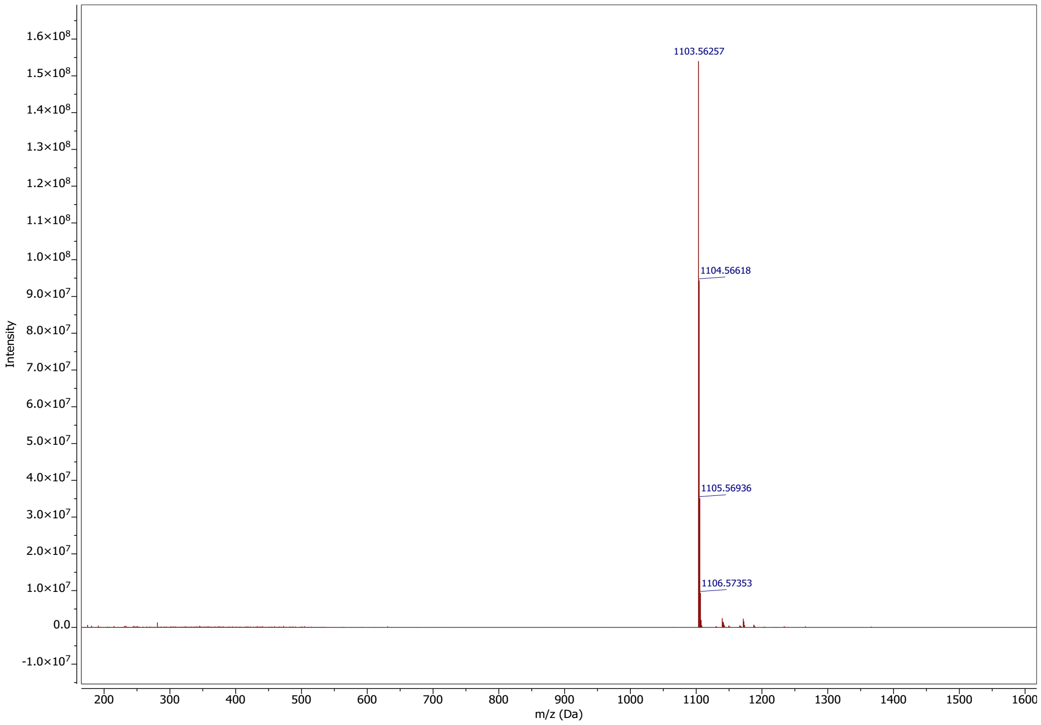

Formula	Target Mass	Theor. Mass	Error (mDa)	Error (ppm)
$C_{54}H_{87}O_{23}$	1103.56257	1103.56436	-1.79	-1.62

Figure SII-9. HRMS spectrum of primulasaponin III (**18**) in negative ionization mode

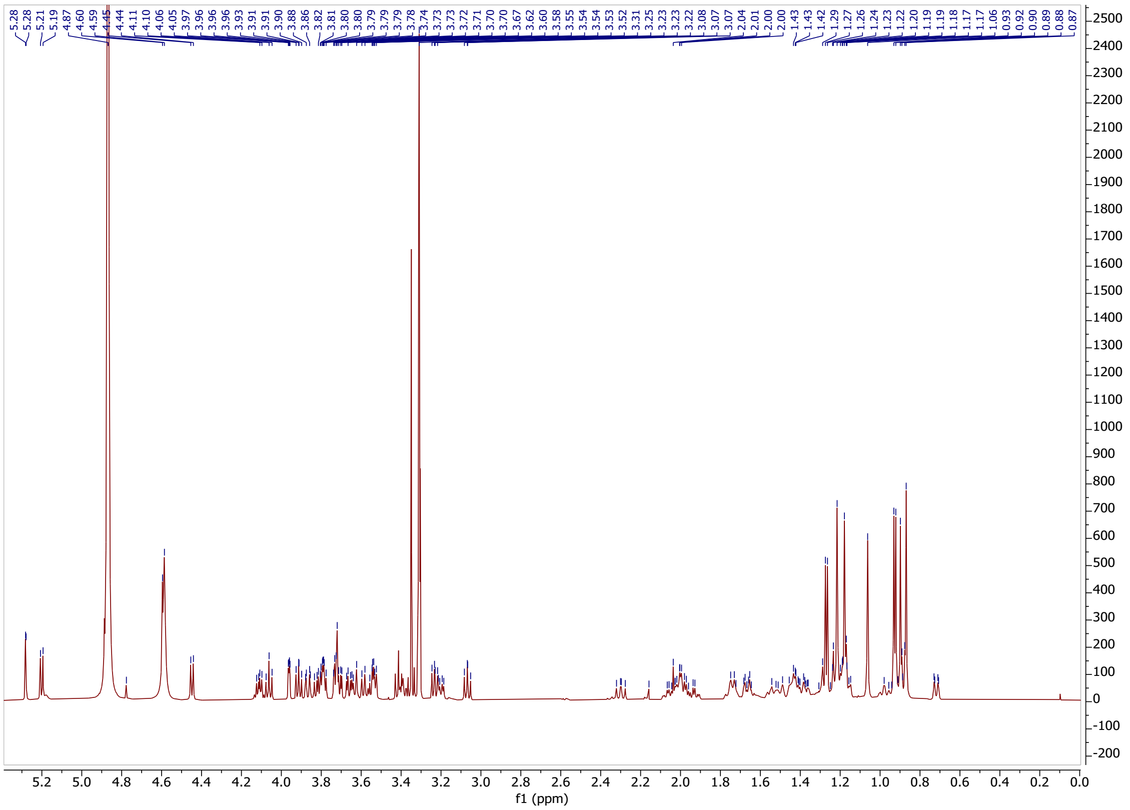

Figure SII-10. ^{1}H NMR spectrum of primulasaponin III (**19**) in CD_3OD

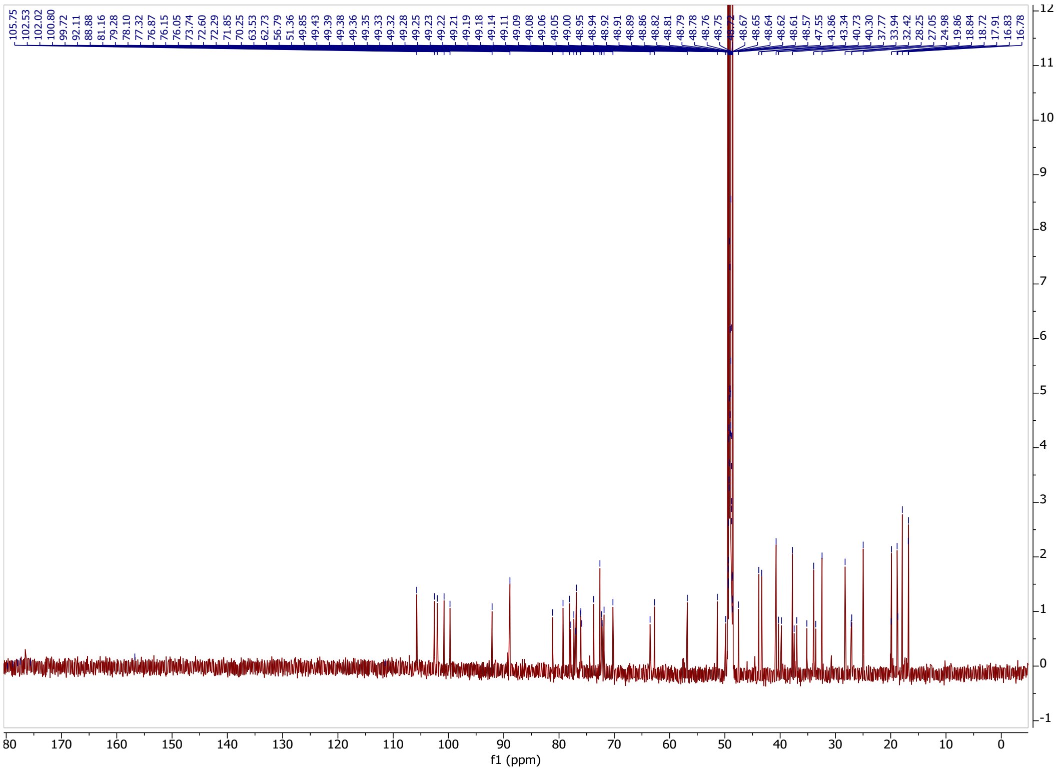

Figure SII-11. ^{13}C NMR spectrum of primulasaponin III (**19**) in CD_3OD

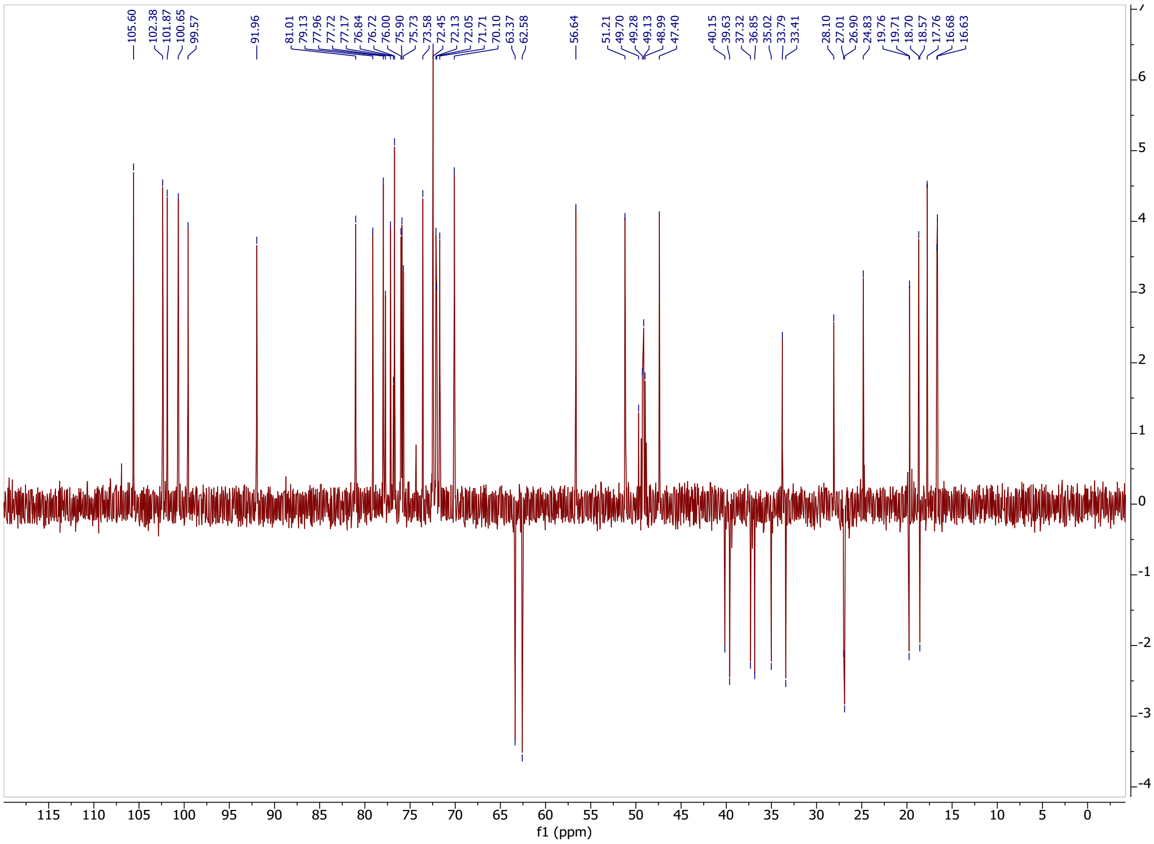

Figure SII-12. ^{13}C DEPT-135 NMR spectrum of primulasaponin III (**19**) in CD_3OD

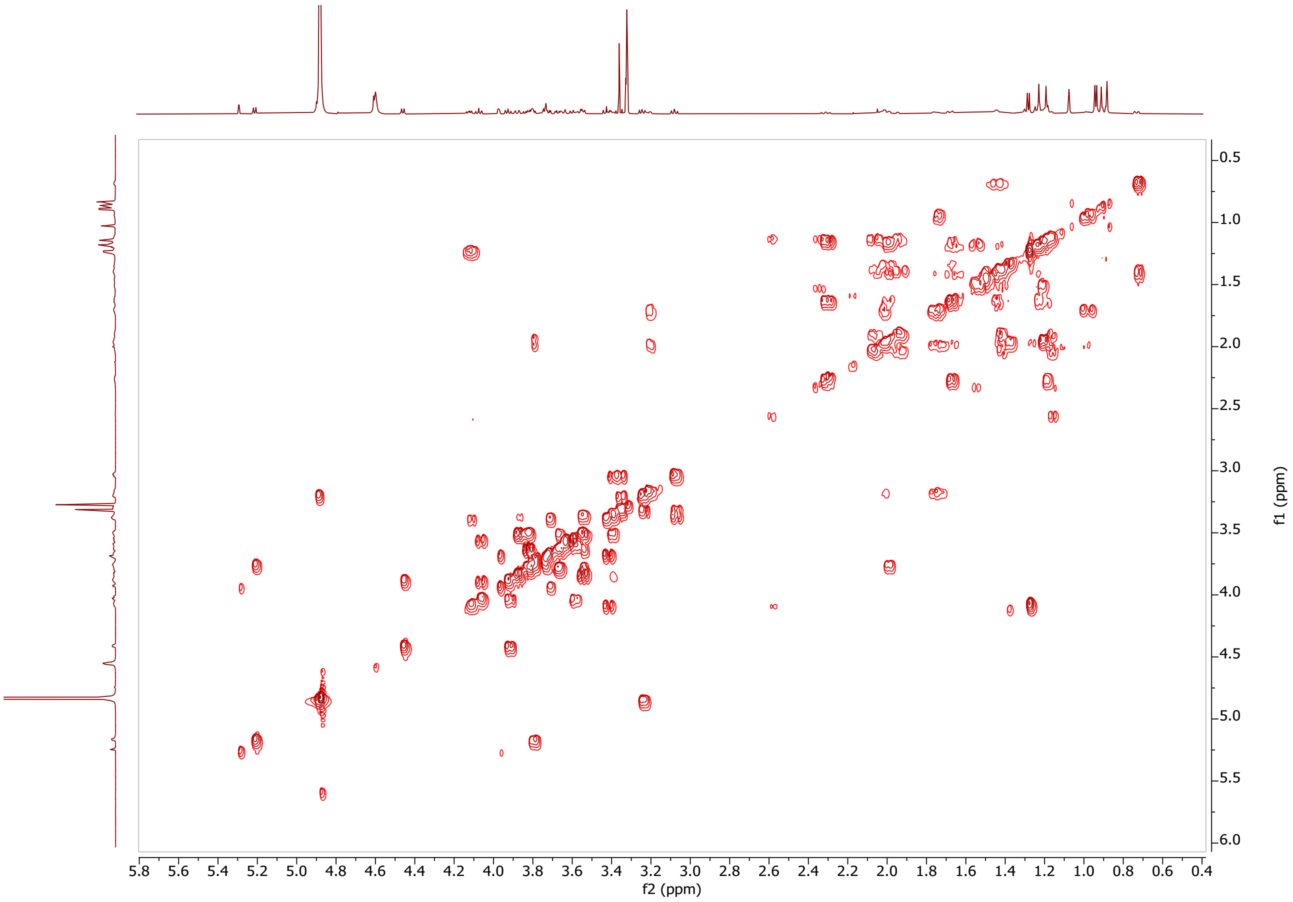

Figure SII-13. COSY spectrum of primulasaponin III (**19**) in CD_3OD

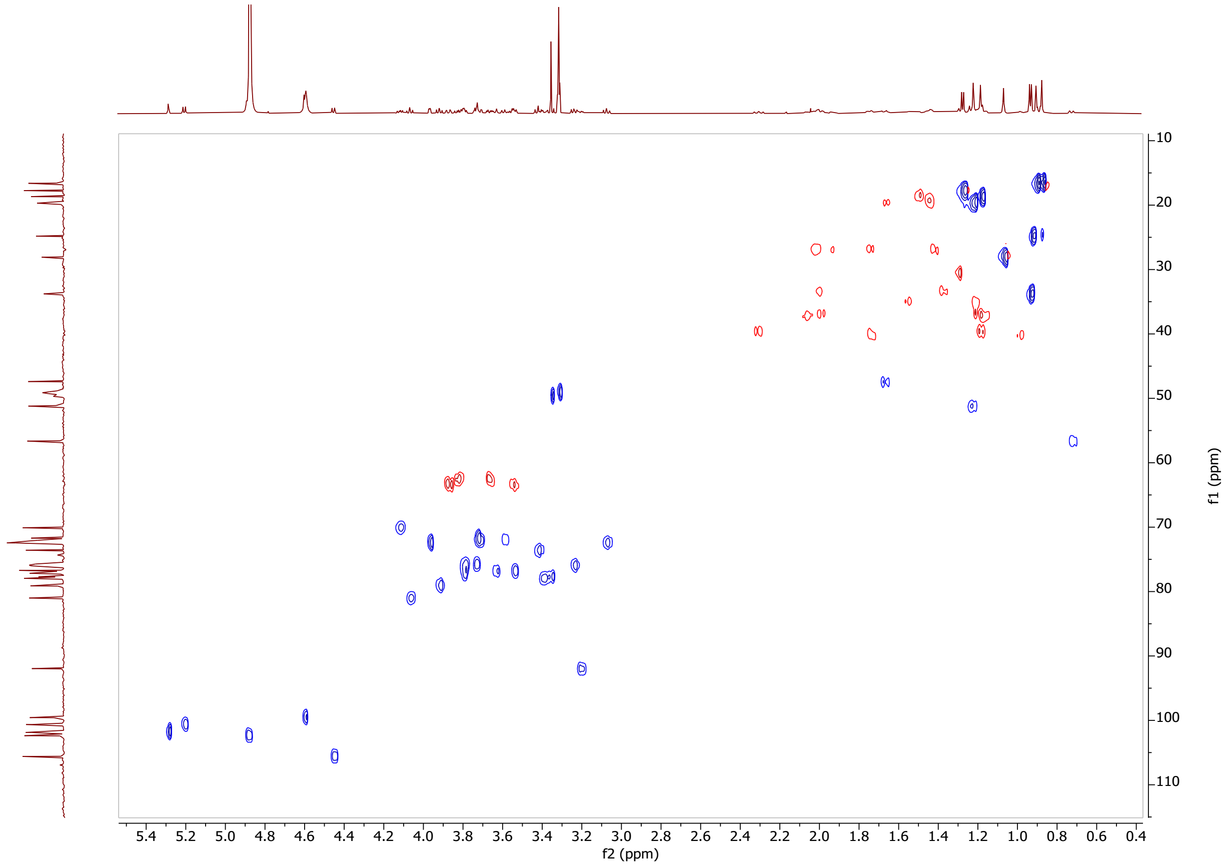

Figure SII-14. HSQC spectrum of primulasaponin III (**19**) in CD_3OD

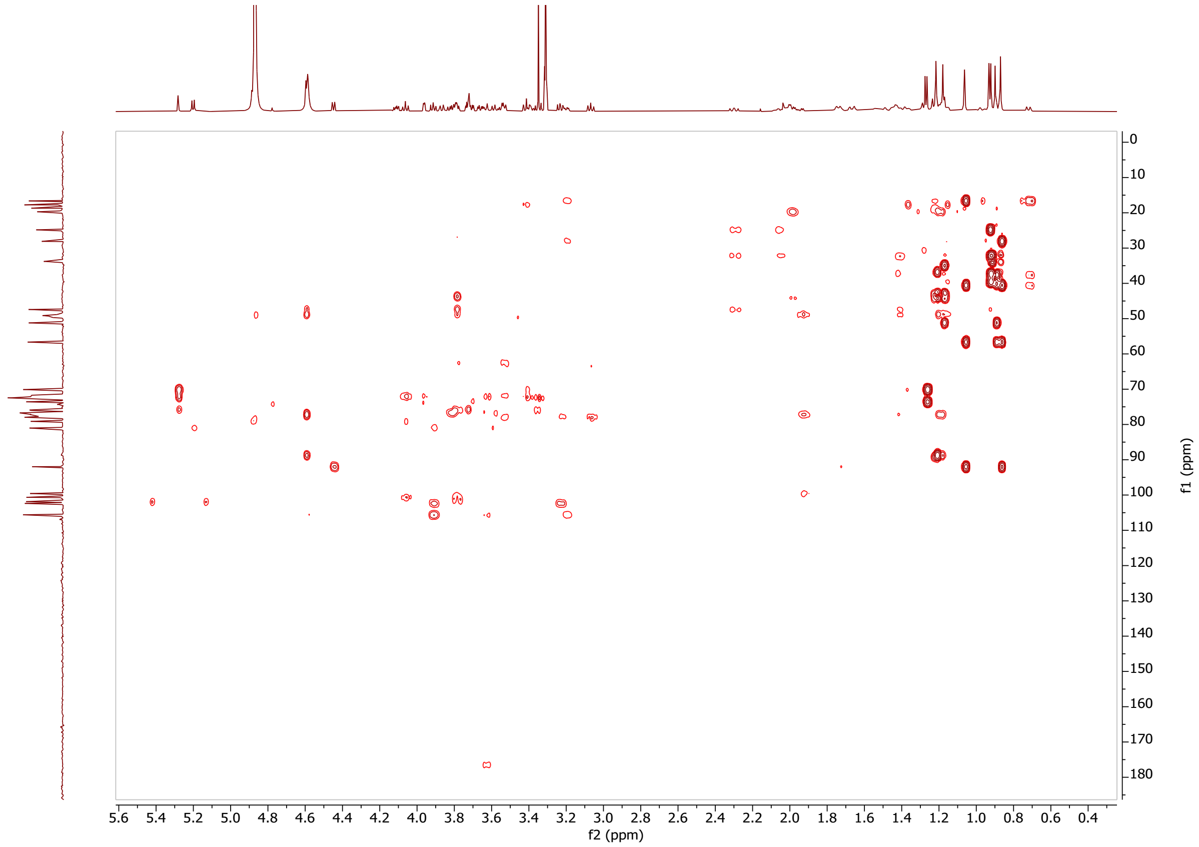

Figure SII-15. HMBC spectrum of primulasaponin III (**19**) in CD_3OD

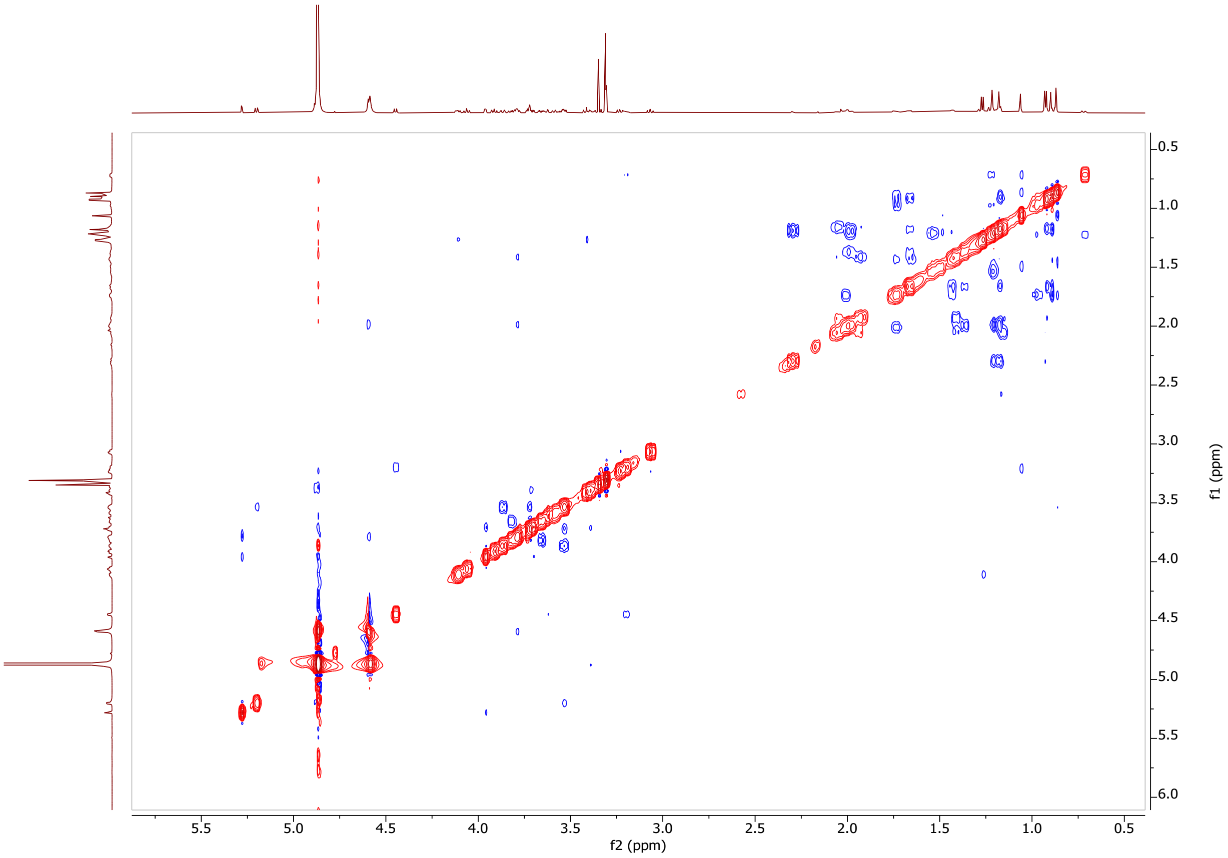

Figure SII-16. ROESY spectrum of primulasaponin III (**19**) in CD_3OD

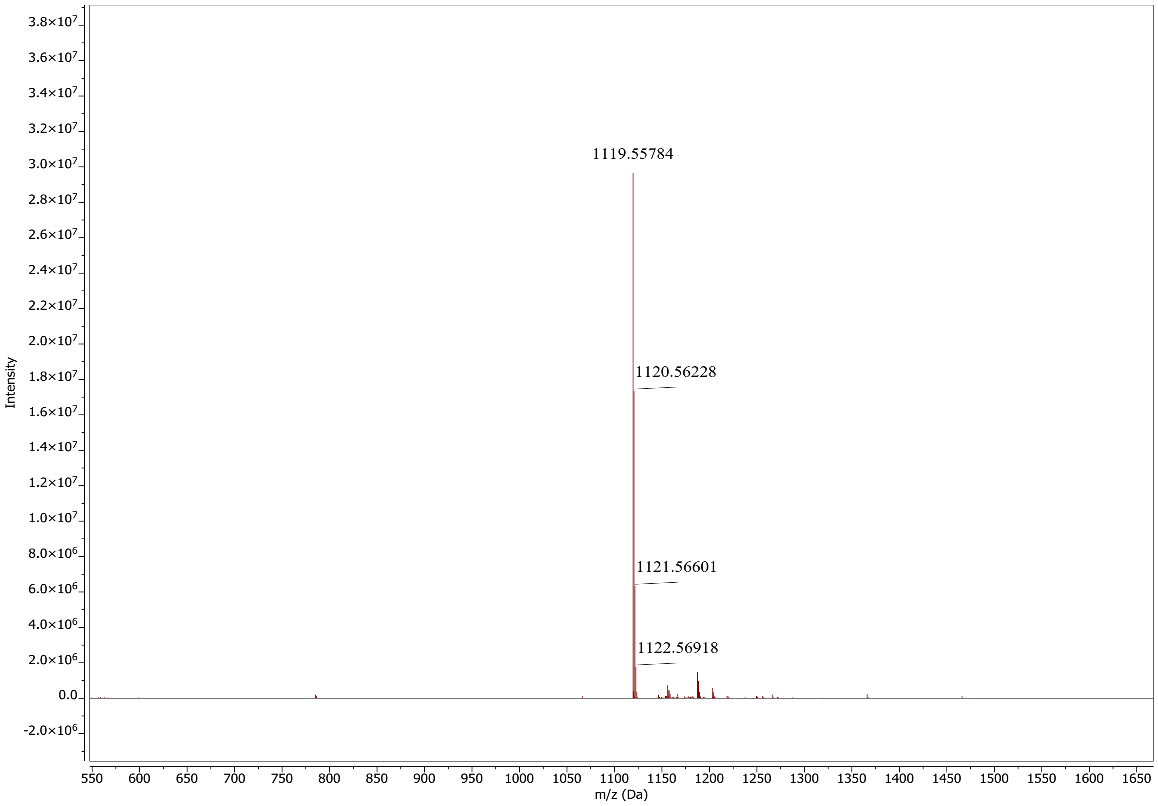

Formula	Target Mass	Theor. Mass	Error (mDa)	Error (ppm)
$C_{54}H_{87}O_{24}$	1119.55784	1119.55928	-1.44	-1.28

Figure SII-17. HRMS spectrum of primulasaponin III (**19**) in negative ionization mode

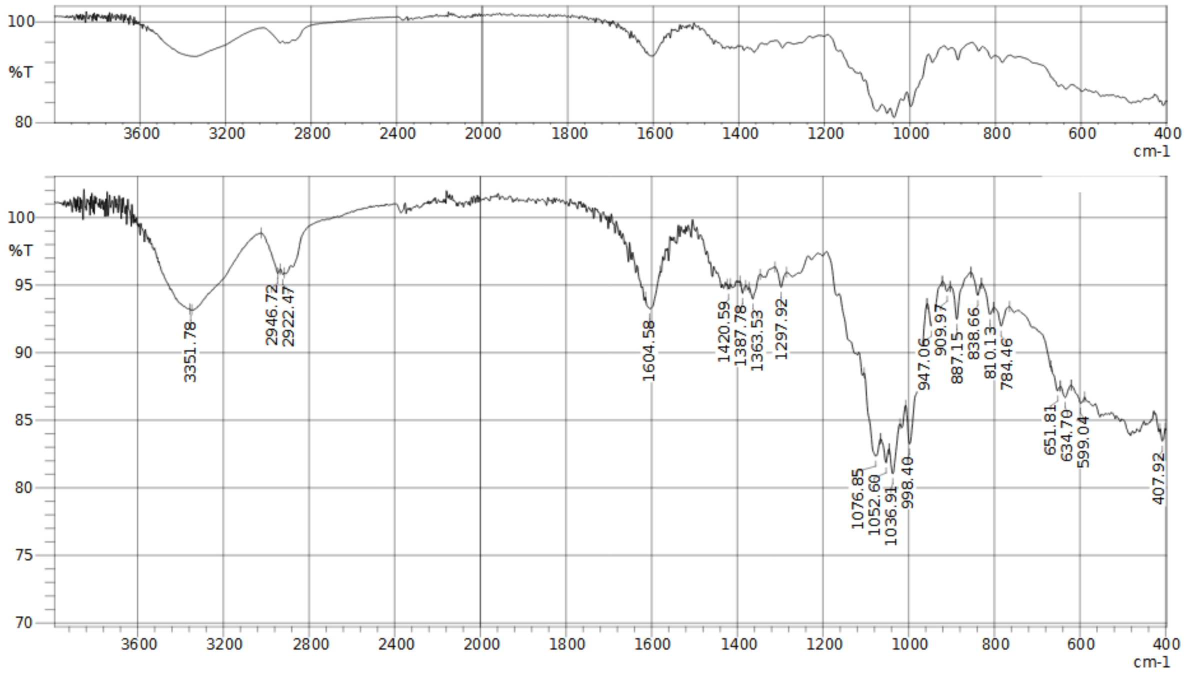

Figure SII-18. IR (ATR) spectrum of primulasaponin III (**19**)

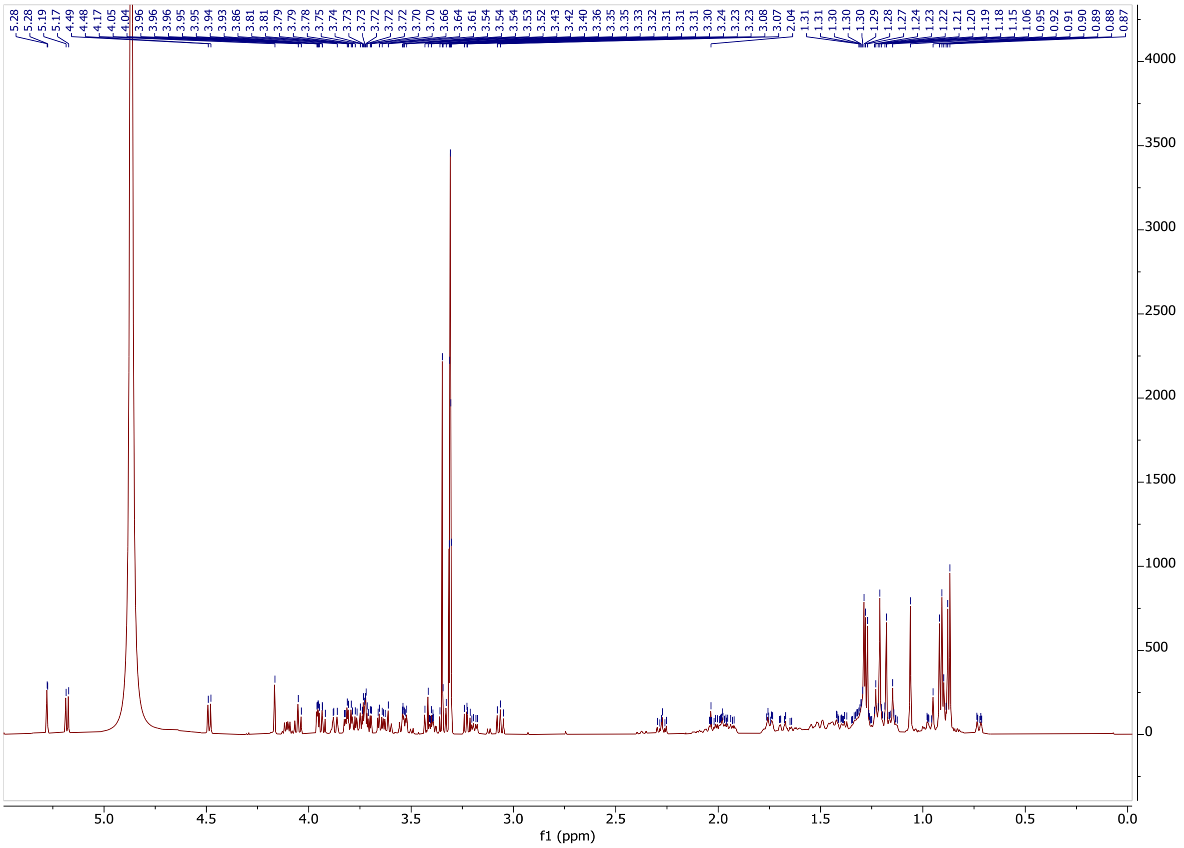

Figure SII-19. ^{1}H NMR spectrum of primulasaponin IV (**20**) in CD_3OD

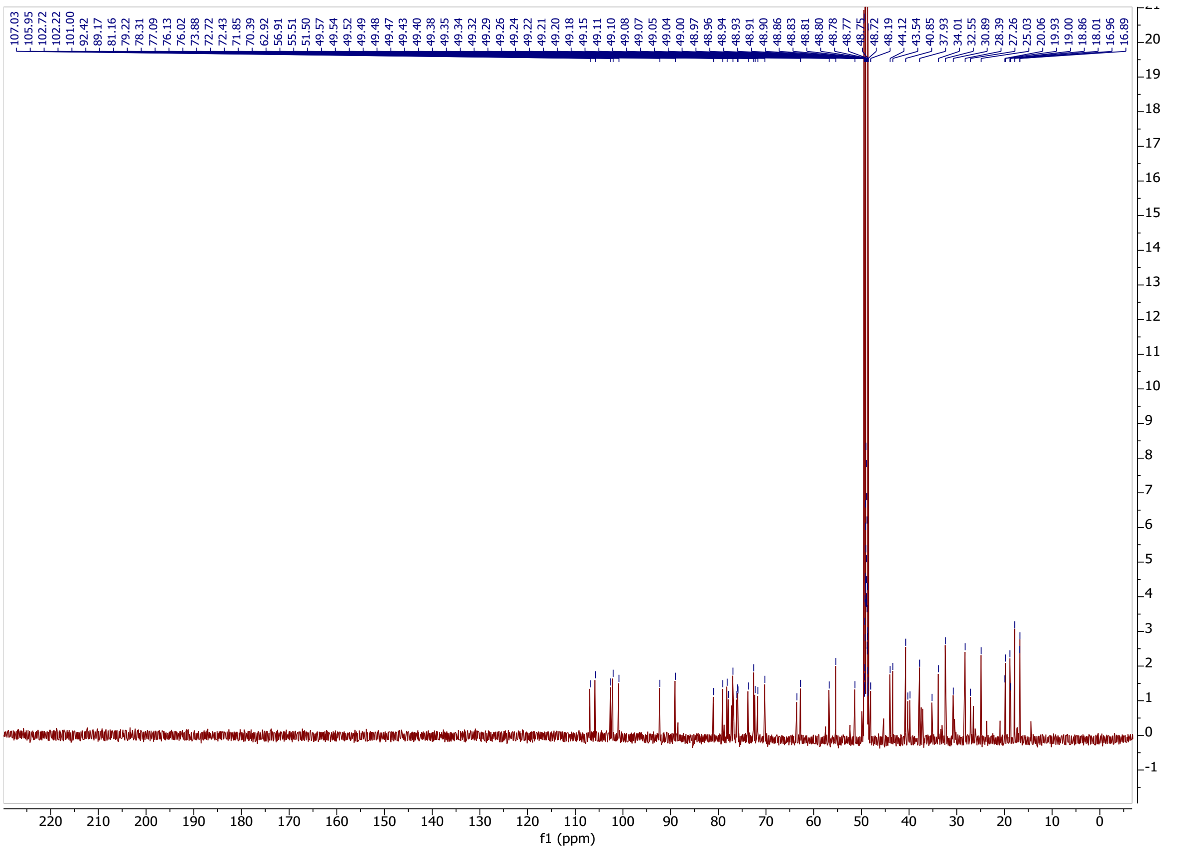

Figure SII-20. ^{13}C NMR spectrum of primulasaponin IV (**20**) in CD_3OD

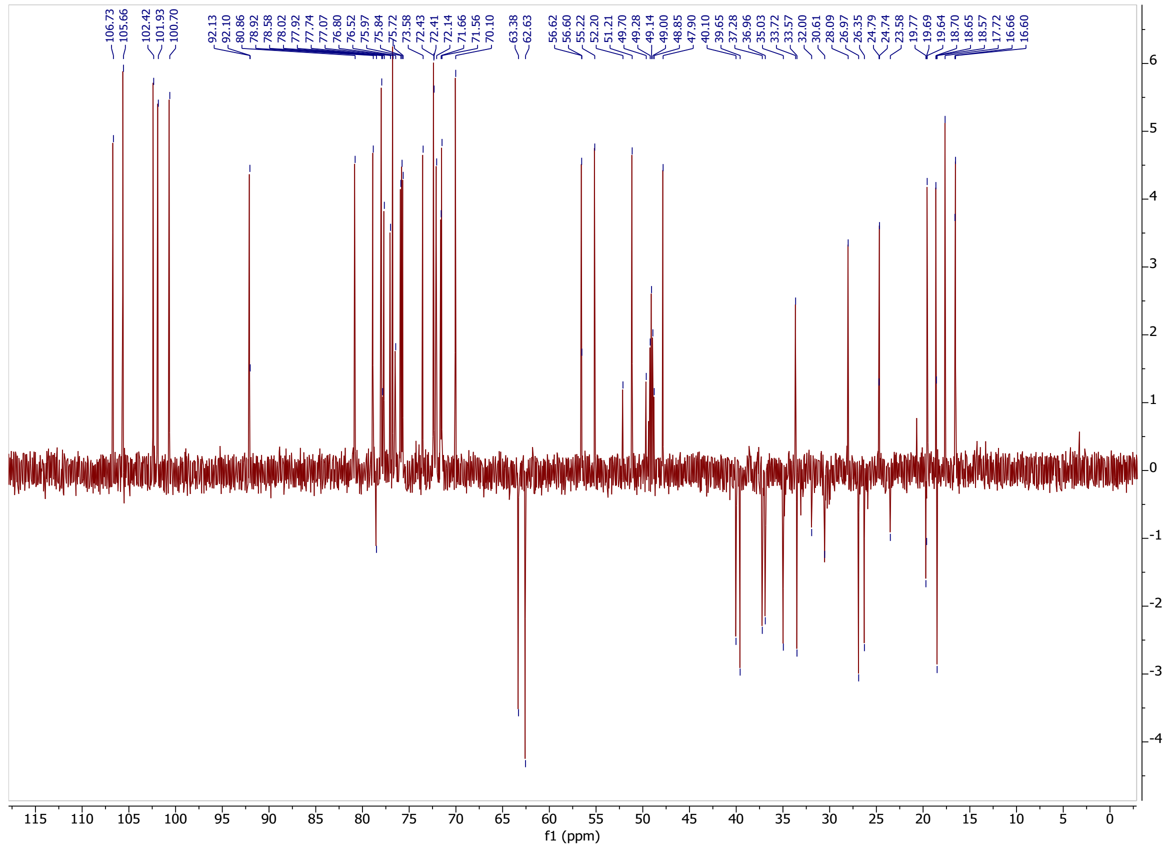

Figure SII-21. ^{13}C DEPT-135 NMR spectrum of primulasaponin IV (**20**) in CD_3OD

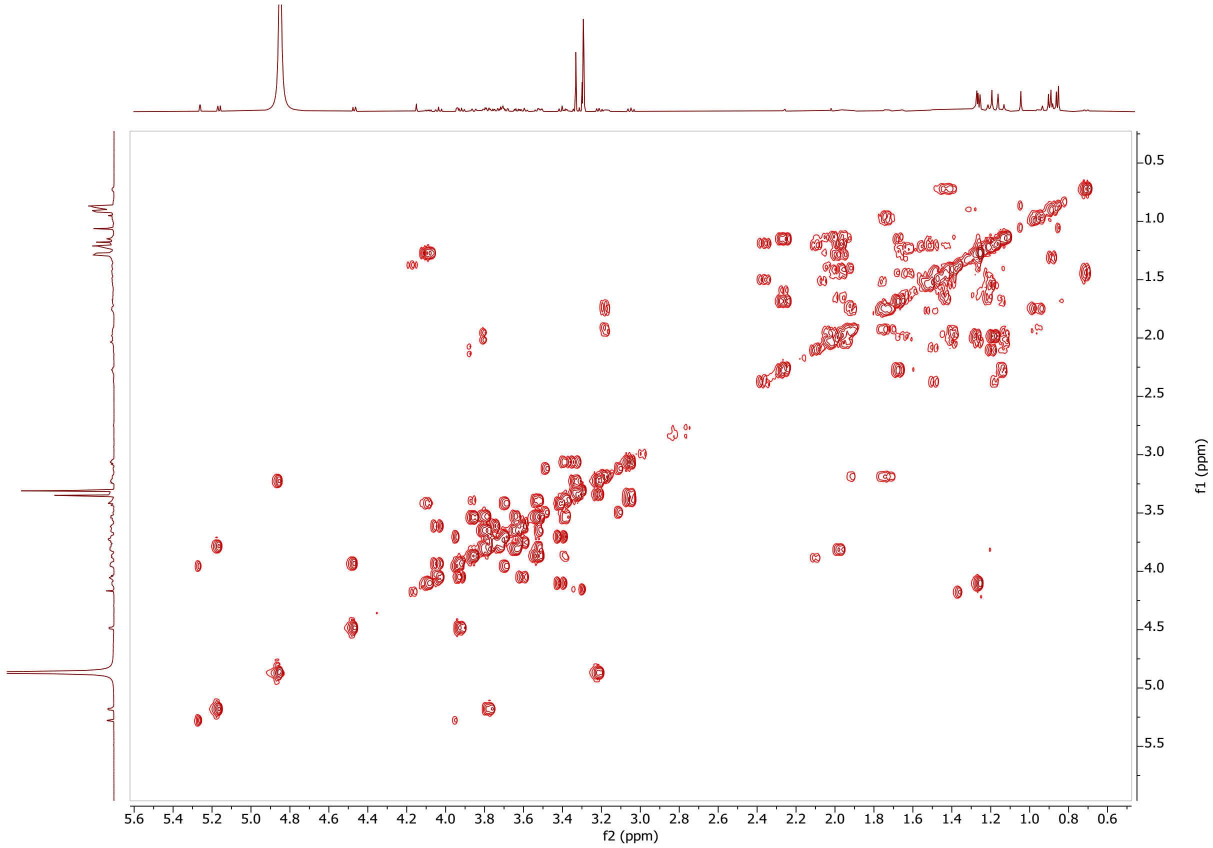

Figure SII-22. COSY spectrum of primulasaponin IV (**20**) in CD_3OD

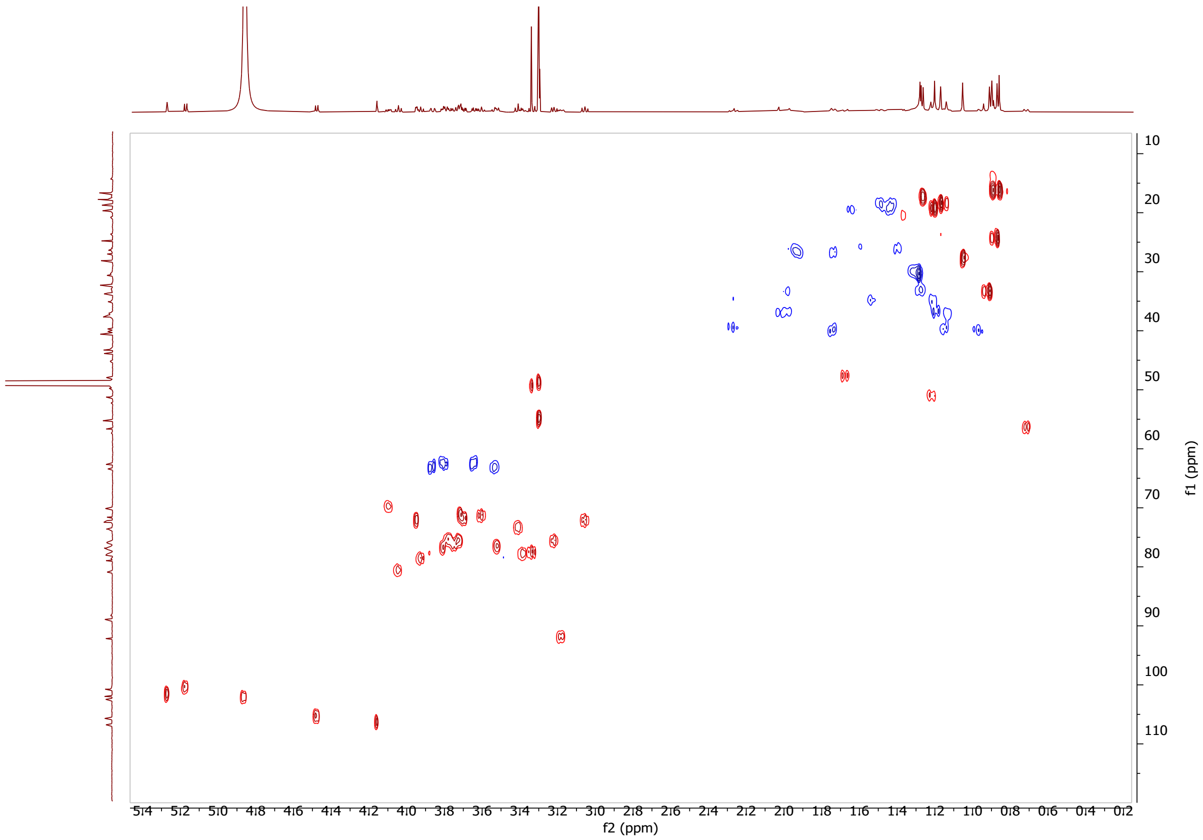

Figure SII-23. HSQC spectrum of primulasaponin IV (**20**) in CD_3OD

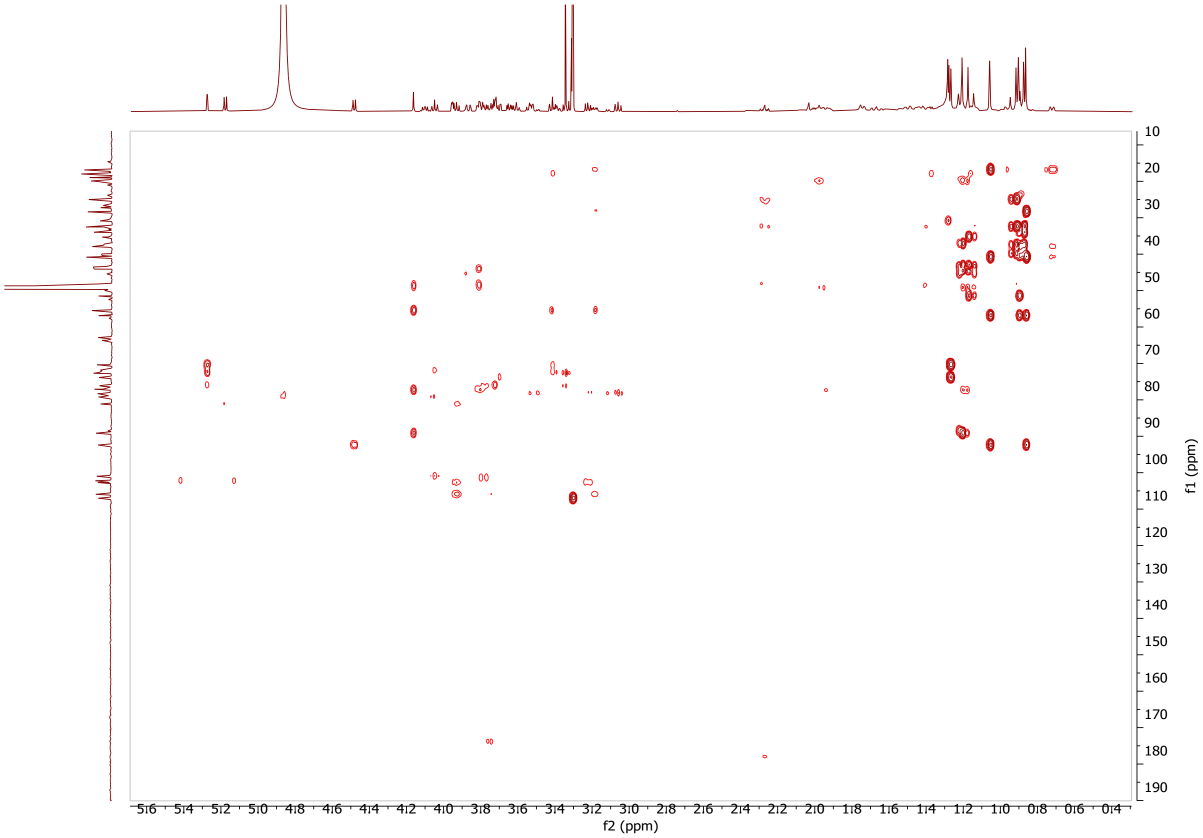

Figure SII-24. HMBC spectrum of primulasaponin IV (**20**) in CD_3OD

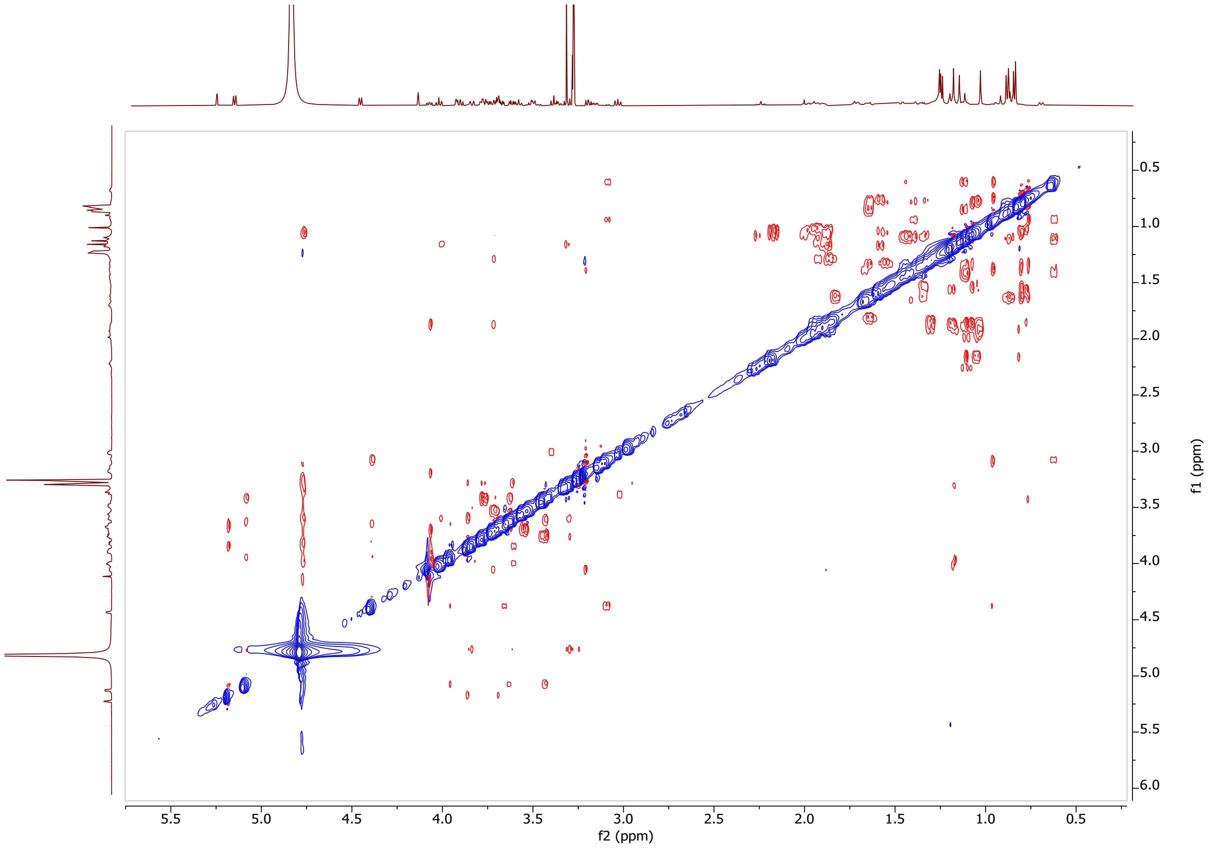

Figure SII-25. ROESY spectrum of primulasaponin IV (**20**) in CD_3OD

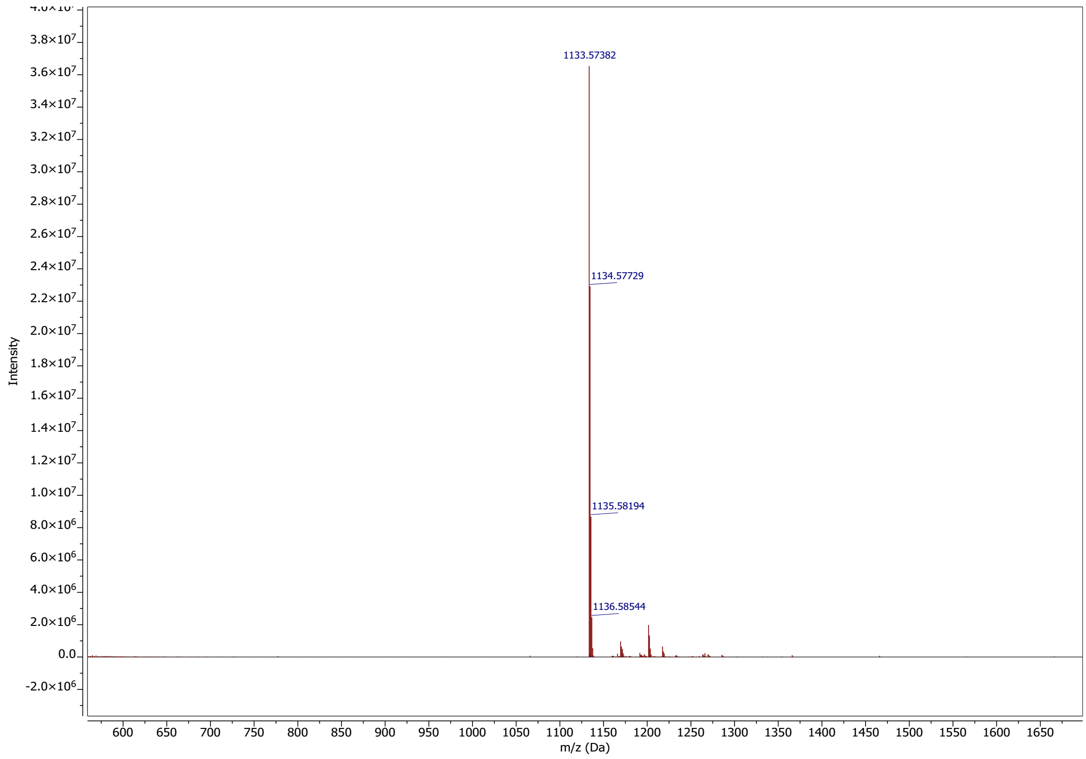

Formula	Target Mass	Theor. Mass	Error (mDa)	Error (ppm)
$C_{55}H_{89}O_{24}$	1133.57382	1133.57493	-1.11	-0.98

Figure SII-26. HRMS spectrum of primulasaponin IV (**20**) in negative ionization mode

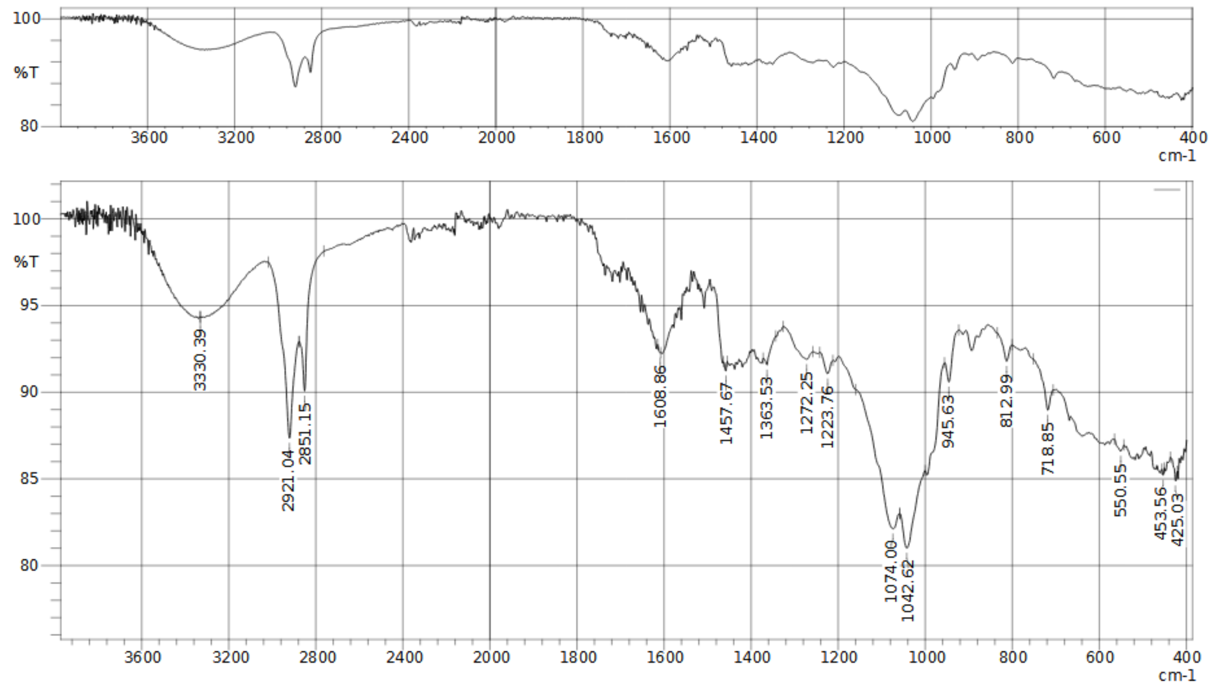

Figure SII-27. IR (ATR) spectrum of primulasaponin IV (**20**)

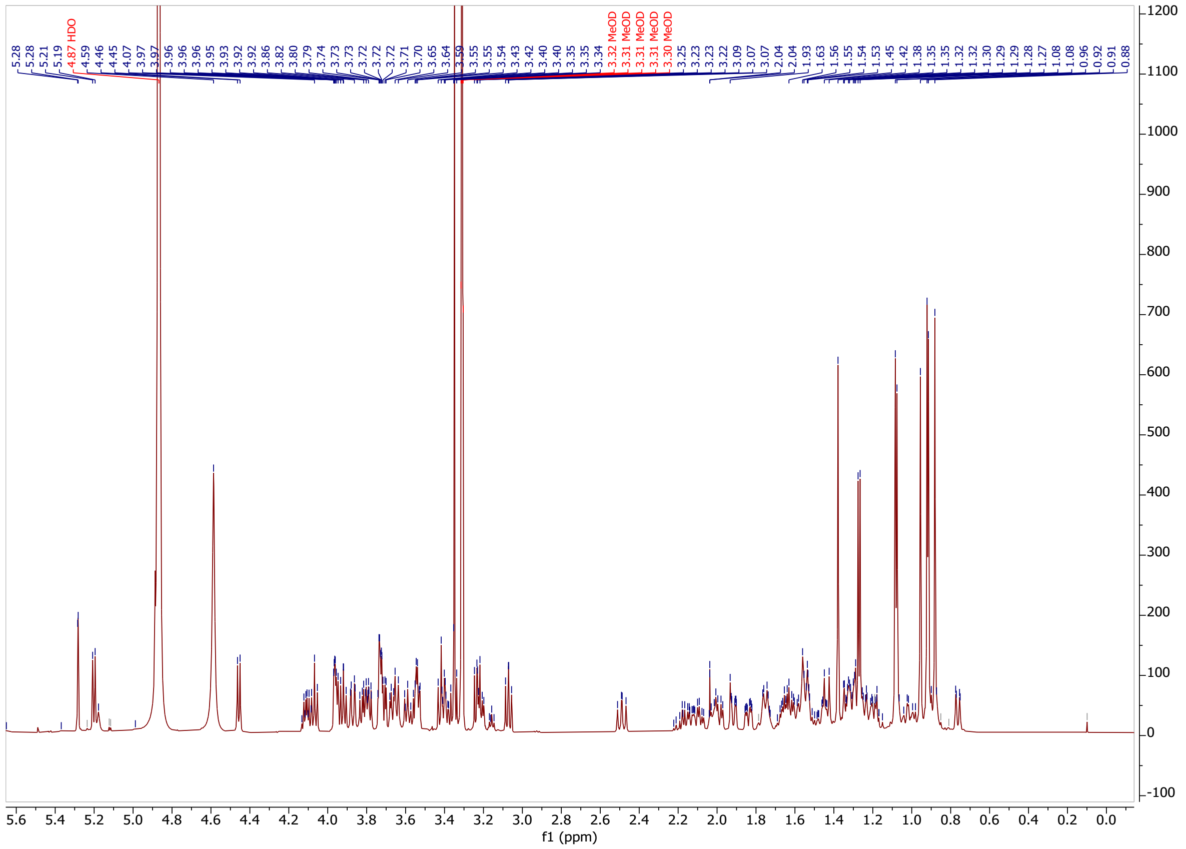

Figure SII-28. ^{1}H NMR spectrum of primulasaponin V (**21**) in CD_3OD

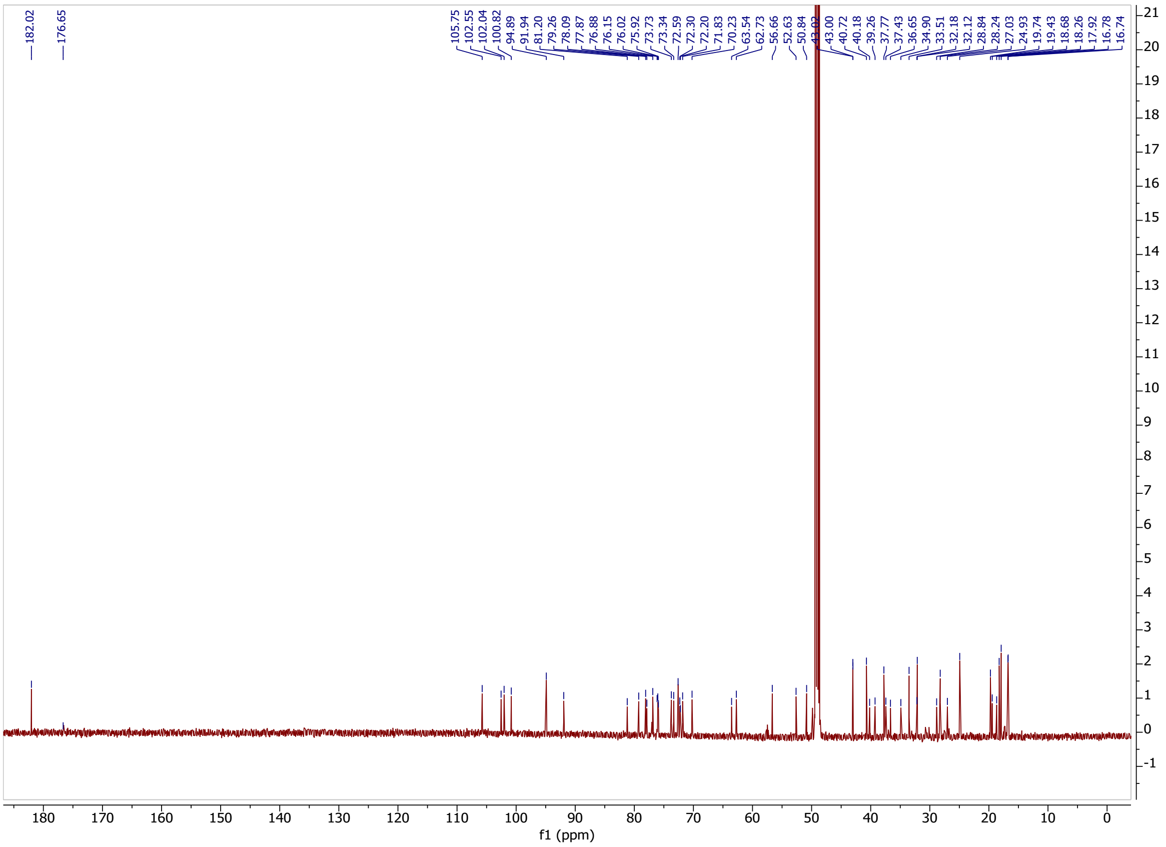

Figure SII-29. ^{13}C NMR spectrum of primulasaponin V (**21**) in CD_3OD

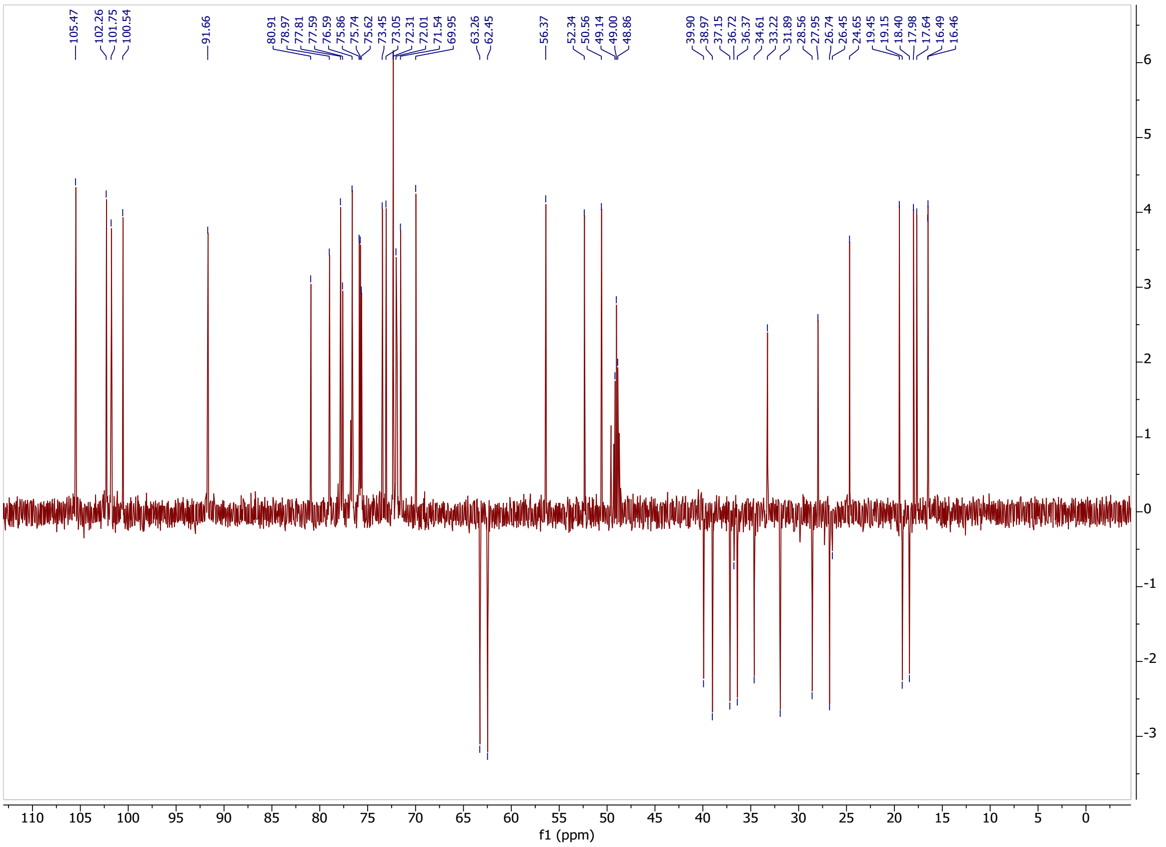

Figure SII-30. ^{13}C DEPT-135 NMR spectrum of primulasaponin V (**21**) in CD_3OD

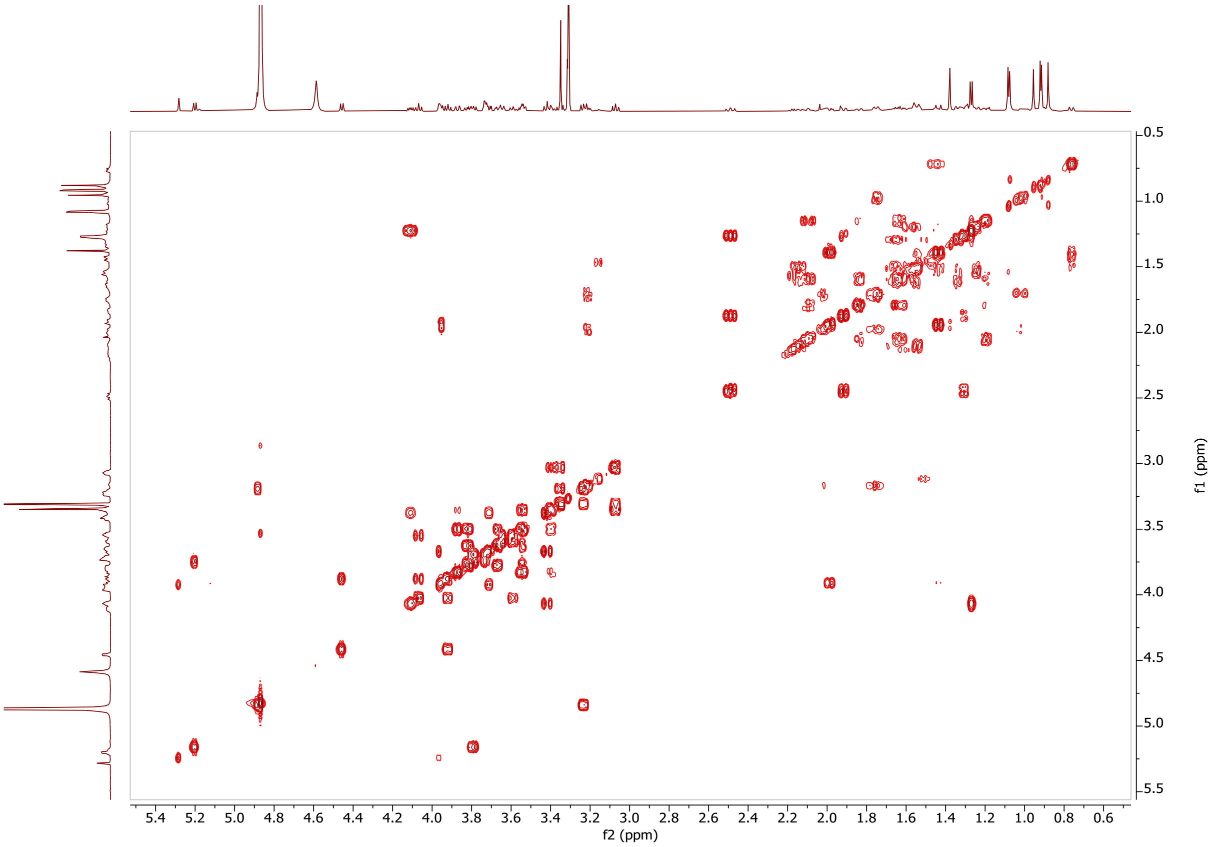

Figure SII-31. COSY spectrum of primulasaponin V (**21**) in CD_3OD

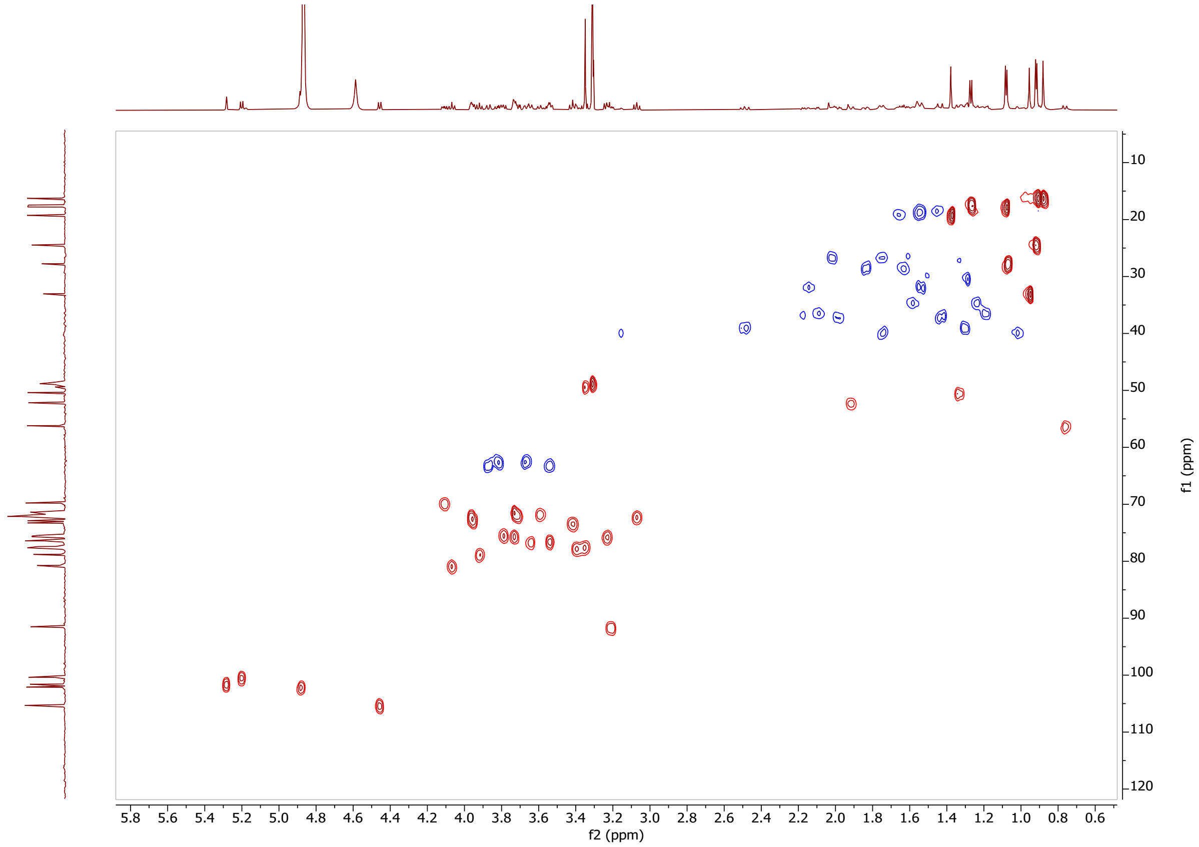

Figure SII-32. HSQC spectrum of primulasaponin V (**21**) in CD_3OD

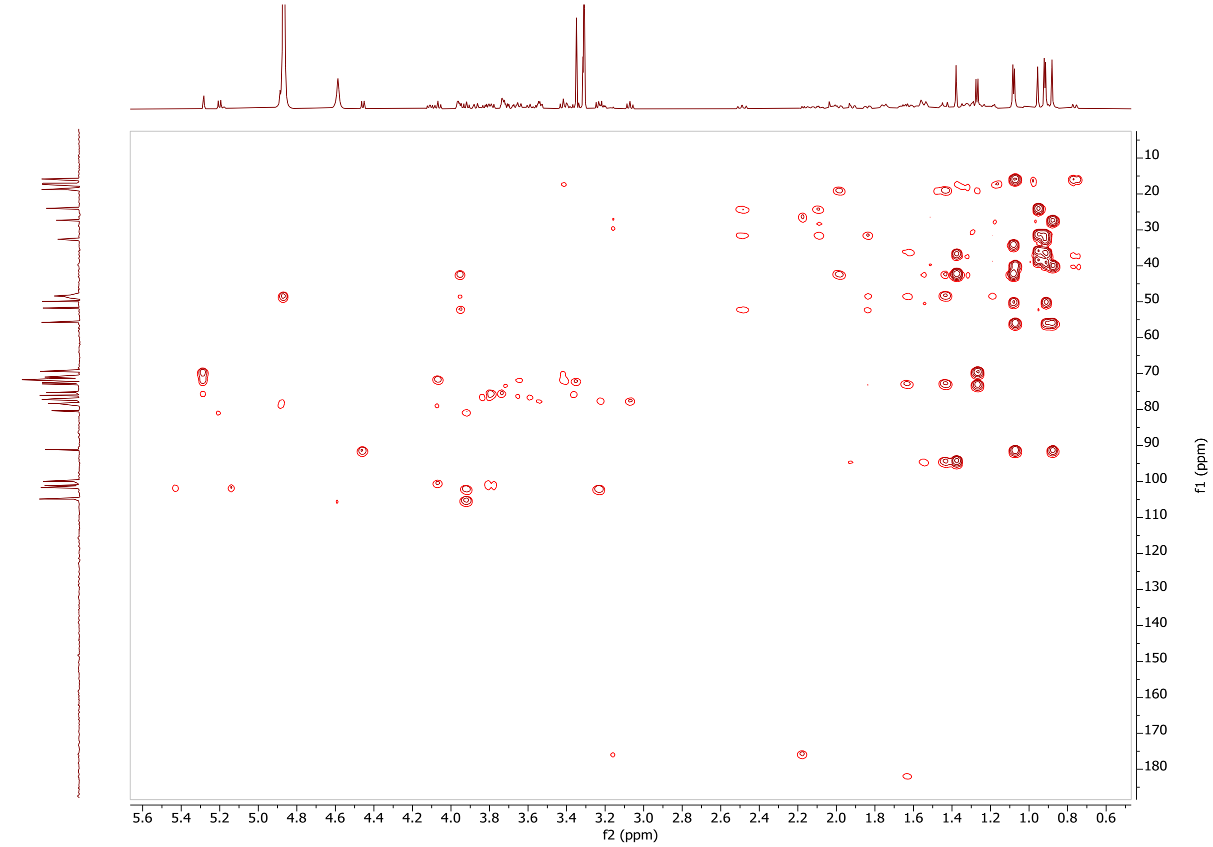

Figure SII-33. HMBC spectrum of primulasaponin V (**21**) in CD_3OD

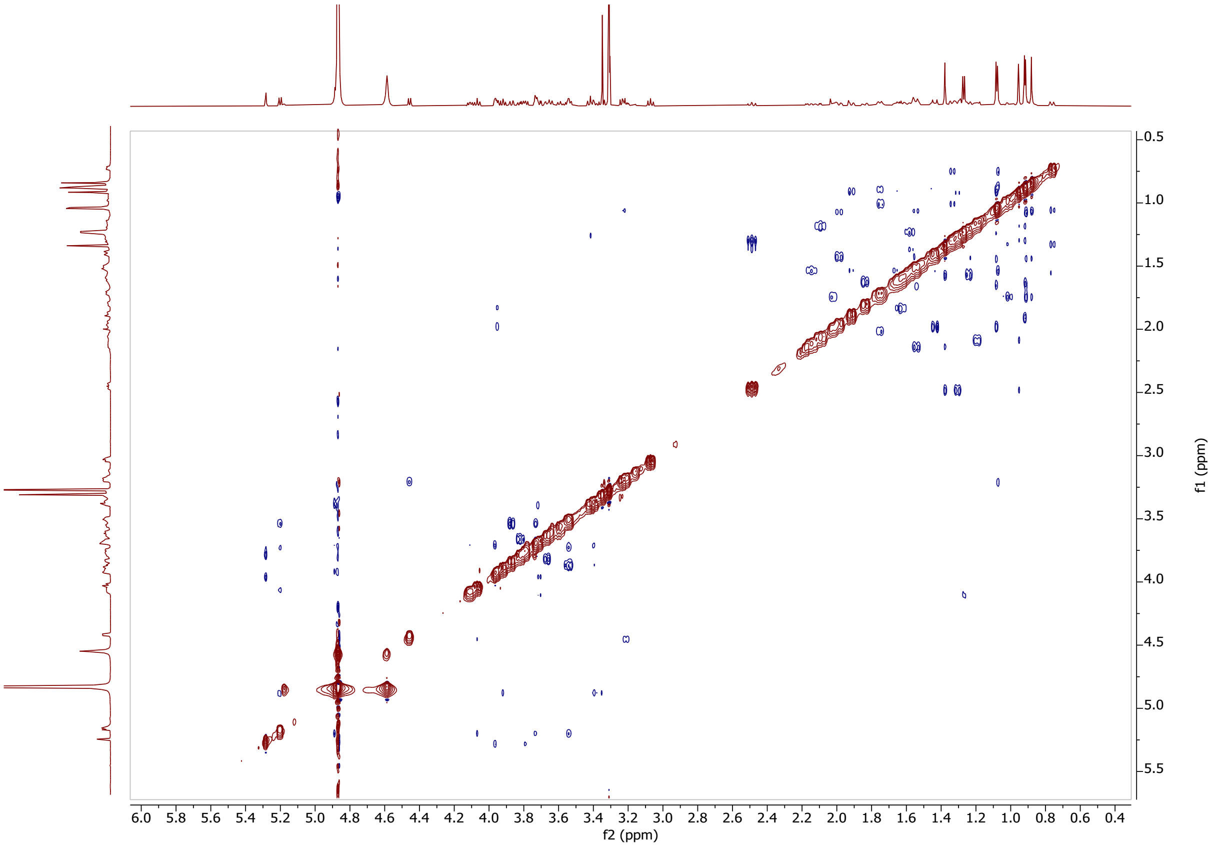

Figure SII-34. ROESY spectrum of primulasaponin V (**21**) in CD_3OD

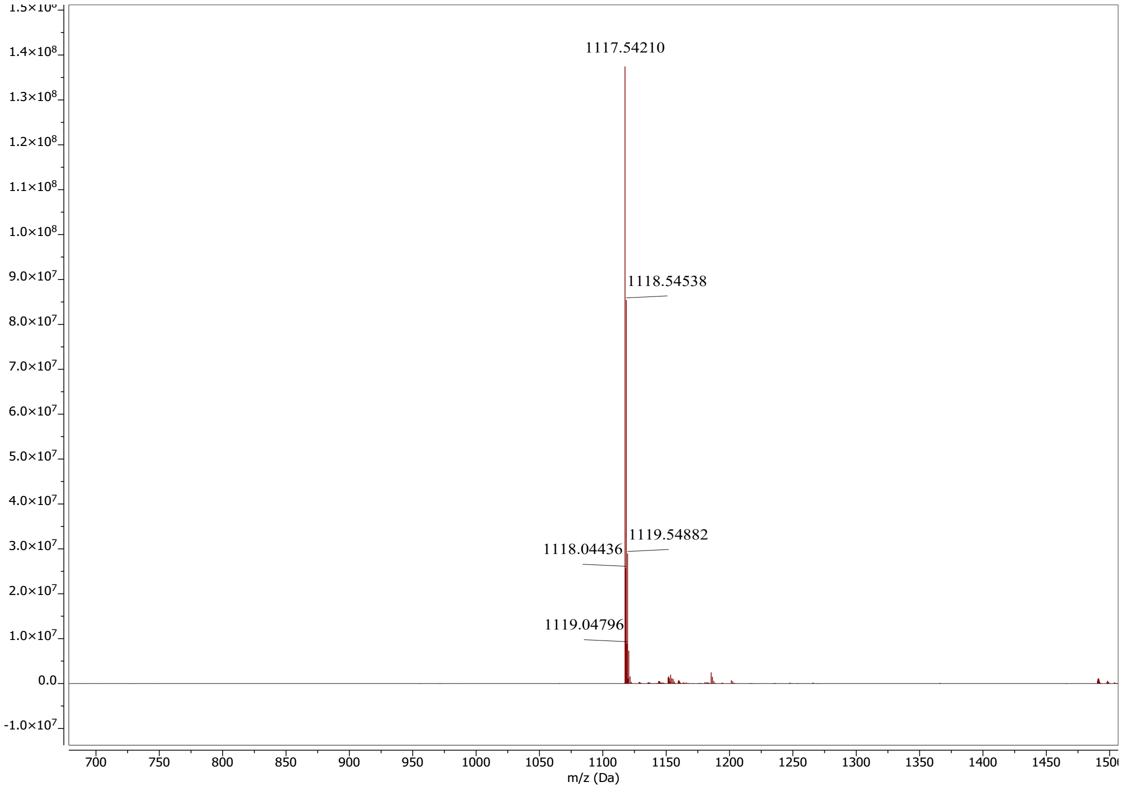

Formula	Target Mass	Theor. Mass	Error (mDa)	Error (ppm)
$C_{54}H_{85}O_{24}$	1117.54210	1133.54363	-1.53	-1.37

Figure SII-35. HRMS spectrum of primulasaponin V (**21**) in negative ionization mode

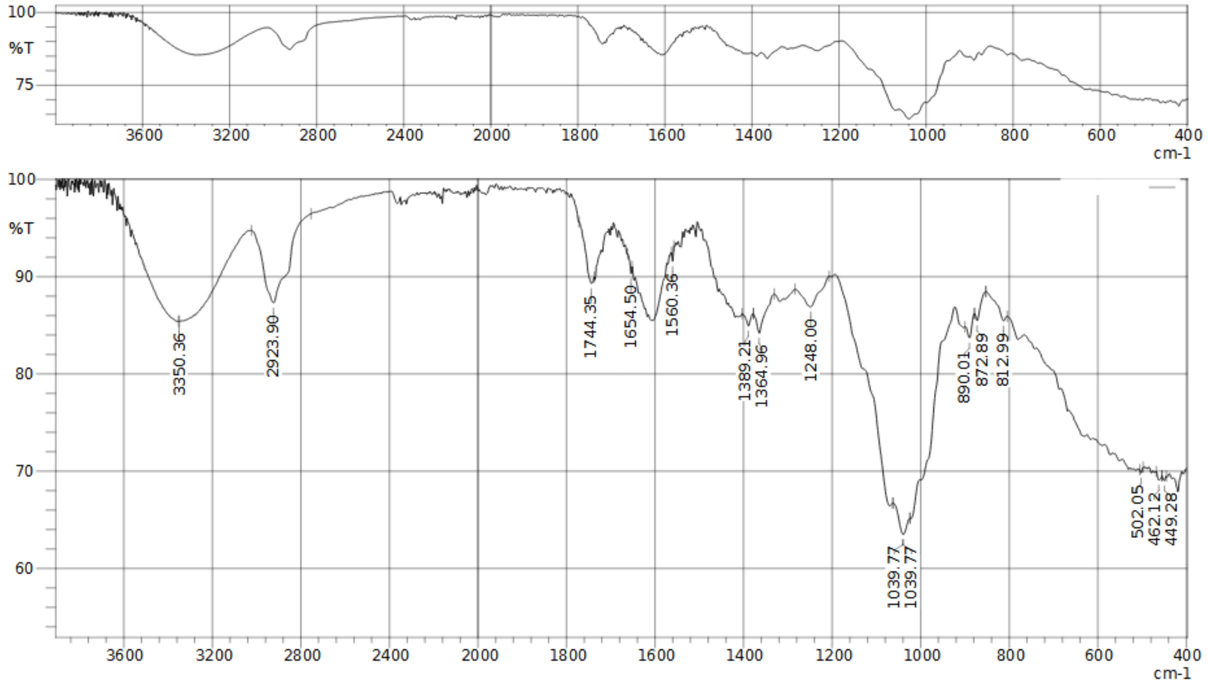

Figure SII-36. IR (ATR) spectrum of primulasaponin V (**21**)
